# The cost-effectiveness of predictive algorithm guided primary antidepressant treatment: economic evaluation of the multinational PReDicT randomised controlled trial

**DOI:** 10.1192/bjo.2026.11021

**Published:** 2026-04-13

**Authors:** Nataša Perić, Susanne Mayer, Timea Helter, Jürgen Deckert, Philip Gorwood, Victor Perez, Andreas Reif, Henricus G. Ruhe, Dick J. Veltman, Anneke van Schaik, Richard Keith Morriss, Amy Beckenstrom, Gerard R. Dawson, Colin T. Dourish, Rebecca Dias, Jonathan Kingslake, Michael Browning, Judit Simon

**Affiliations:** Department of Health Economics, Center for Public Health, Medical University of Vienna, Vienna, Austria; Department of Psychiatry, Psychosomatics and Psychotherapy, Center of Mental Health, University Hospital of Würzburg, Würzburg, Germany; Institute of Psychiatry and Neuroscience of Paris, INSERM U1266, Université Paris Cité, Paris, France; GHU Paris Psychiatry & Neurosciences, Sainte-Anne Hospital, Paris, France; Hospital del Mar Medical Research Institute (IMIM), Barcelona, Spain; Biomedical Research Networking Center in Mental Health, Madrid, Spain; Department of Psychiatry, Psychosomatic Medicine and Psychotherapy, University Hospital Frankfurt – Goethe University, Frankfurt am Main, Germany; Department of Psychiatry, Radboud UMC, Nijmegen, The Netherlands; Donders Institute for Brain, Cognition and Behavior, Radboud University, Nijmegen, The Netherlands; Department of Psychiatry, Amsterdam Neuroscience, Amsterdam UMC, Amsterdam, The Netherlands; Department of Psychiatry, Amsterdam Public Health Research Institute, Amsterdam UMC, Amsterdam, The Netherlands; Division of Psychiatry and Applied Psychology, University of Nottingham, Nottingham, UK; P1vital Ltd, Wallingford, UK; P1vital Products Limited, Wallingford, UK; Department of Psychiatry, University of Oxford, Oxford, UK; Oxford Health NHS Foundation Trust, Warneford Hospital, Oxford, UK

**Keywords:** Depression, primary health care, health economics, quality of life, precision medicine

## Abstract

**Background:**

The PReDicT study showed that predictive algorithm-guided antidepressant treatment reduces anxiety and improves functioning in patients with depression.

**Aims:**

To estimate the costs, outcomes and cost-effectiveness of the PReDicT test compared with treatment as usual (TAU) for primary depression care in five European countries.

**Method:**

Within-trial economic analysis was conducted over 24 weeks from the health/social care and societal perspectives alongside the PReDicT trial (NCT02790970) in France, Germany, The Netherlands, Spain, and the UK, according to Consolidated Health Economic Evaluation Reporting Standards guidelines. We calculated quality-adjusted life-years (QALYs) based on the EQ-5D-5L, capability-weighted life-years based on the Oxford Capabilities Questionnaire – Mental Health (OxCAP-MH) (Germany and UK only), and costs for 2018 (€). Multiple imputation for missing data, multivariable regression for cost and outcome differences, and bootstrapping and sensitivity analyses for uncertainty were conducted.

**Results:**

There were significant outcome improvements (EQ-5D-5L PRedicT: +0.139; TAU: +0.140) and societal cost reductions (PRedicT: −€2589; TAU: −€2602) in both groups (*N* = 913) between the before and during trial periods. In the UK and Germany (*n* = 619), the PReDicT group showed significant additional capability well-being gains (OxCAP-MH: +2.127, *p* = 0.021). Cost-effectiveness probabilities ranged from 46 to 59% at trial level, but exceeded 80% in the UK. Results remained stable across different sensitivity analyses, with societal cost-effectiveness improved for those (self-)employed.

**Conclusions:**

We observed potentially meaningful health and economic benefits of closely monitored antidepressant treatment, as implemented in both treatment and control arms of the PReDicT trial. The PReDicT test itself had some added benefits in improved capabilities and productivity, however, with great uncertainty and country-level variations in cost-effectiveness.

Depressive disorders are among the most prevalent mental health conditions, posing a major public health concern because of associated morbidity and mortality.^1^ They impede usual functioning, cause negative thoughts, are associated with major excess physical health burden and have an overall detrimental impact on quality of life.^2^ In Europe, the socioeconomic burden of depressive disorders was estimated at 1% of the national income (gross domestic product) or over €118 billion, making it one of the costliest brain disorders.^3^ Reduced productivity owing to higher absenteeism and lower productivity at work significantly contributes to the overall societal burden.^4^ There are also major effects on family members and caregivers, with considerable spillover costs and significant effects on their own health and well-being.^5^ Recent evidence indicates the additional value of prevention and improved management of depression in terms of reduced non-mental healthcare costs as well.^6,7^

Beside psychological–behavioural therapies, antidepressant medication is recommended as a first-line treatment for moderate to severe cases of depression.^8^ However, almost two-thirds of individuals fail to respond to initial antidepressant treatment, causing cycles of different treatments and long delays before reaching therapeutic success.^9^ One way to shorten this delay is by using personalised approaches where the most effective therapy is selected based on patient characteristics with additional intensive monitoring of treatment response.^10,11^ The PReDicT test, a digital predictive algorithm, was developed to guide antidepressant treatment selection by early detection of non-response. It is based on behavioural tests of affective cognition (a facial recognition task of emotional expressions) and subjective symptoms of depression to predict response after a week of antidepressant treatment, with an accuracy of 60% based on early self-reported symptom change.^12^ A previous modelling study from England suggested that such monitoring of negative emotional bias in primary care for personalised antidepressant treatment is likely to be a cost-effective option.^13^

The clinical effectiveness of the PReDicT test was compared with treatment as usual (TAU) in the two-arm, multi-site, multinational, open-label, randomised controlled PReDicT trial (2016**–**2019), conducted in primary care settings for depression in five European countries (France, Germany, The Netherlands, Spain and the UK).^14^ Although response rates for depressive symptoms at week 8 were similar between the PReDicT (55.9%) and TAU (51.8%) groups, the PReDicT group showed significantly greater reduction of anxiety at week 8 (measured using the Generalised Anxiety Disorder-7 (GAD-7) questionnaire) and greater improvement in functional outcome at week 24.^15^ The PReDicT test was found to be highly acceptable to patients, particularly those who benefited in terms of information and increased confidence in treatment and/or their doctor.^16^

## Aims

This study assessed the cost, quality-of-life impacts and cost-effectiveness of the PReDicT test compared with TAU as part of a within-trial economic evaluation conducted prospectively alongside the PReDicT trial in five European countries.

## Method

### Study design and patients

The PReDicT trial was conducted across 90 primary depression care sites in France, Germany, The Netherlands, Spain and the UK, between 2016 and 2019. It was registered with ClinicalTrials.gov (identifier NCT02790970). The study complied with all relevant ethical guidelines, the Helsinki Declaration and obtained written patient consent. Approval was granted by the relevant ethics committees in all countries: France (Ile-de-France MDPT-RIAL/MM/2016-AO1054-47), Germany (Universität Würzburg 117/16-sc; Goethe Universität Frankfurt 34/17B), The Netherlands (METC VUmc 2016.294), Spain (CEIC Parc de Salut Mar 2016/6795/I) and the UK (NRES 16/NE/0095).

Antidepressant medication-free patients aged between 18 and 70 years were included if they were considered for treatment with a selective serotonin reuptake inhibitor for the management of a depressive episode. Because of its longer half-life, treatment with fluoxetine was excluded. Exclusion criteria were current treatment with an antidepressant, a previous history of mania or a presentation that required immediate referral to a separate service (e.g. significant suicidal intent requiring enhanced care). After randomisation, all patients completed the PReDicT test before starting antidepressant treatment at baseline (week 0) and a week after (week 1) via an electronic Patient Reported Outcomes (ePRO) system, to quantify early treatment-induced change in negative emotional bias. When the PReDicT test indicated non-response to the antidepressant, the prescribing physician was informed and advised to alter the patient’s antidepressant treatment – either by dose escalation, switching to another compound or augmentation – in accordance with local prescribing guidelines. The PReDicT test did not recommend a specific type of treatment change. Only PReDicT group participants with indicated non-response at week 1 repeated the PReDicT test at week 2 and had their test results shared again with the prescribing physicians. The PReDicT test was not repeated afterwards and all patients were treated according to local guidelines. Self-reported clinical outcome measurements (weekly until week 8, and then monthly) were collected via the ePRO system for up to a maximum of 48 weeks. Patients in the PReDicT group were able to see their test results and self-monitor their depression symptoms (Quick Inventory of Depressive Symptomatology, 16-item self-report version (QIDS-SR-16)) from week 4 onward. Further details of the study protocol, the clinical effectiveness and patient acceptability results are described elsewhere.^12,14,16^ Health economic data, including health-related quality-of-life (HRQoL) instruments, were collected at baseline and every 4 weeks afterwards via ePRO.

This within-trial health economic analysis was conducted over a 24-week follow-up period from the healthcare, health and social care, and societal perspectives, in line with the different national health technology assessment (HTA) requirements based on an intention-to-treat sample. The 24-week time horizon was chosen to align with the clinical analysis and ensure robustness of the health economic results, given the substantial decline in data completeness at later follow-up. Results are reported according to the Consolidated Health Economic Evaluation Reporting Standards checklist^17^ (Supplementary Table 1 available at https://doi.org/10.1192/bjo.2026.11021).

### Outcomes

The primary economic analysis was an incremental cost-utility analysis with quality-adjusted life-years (QALYs) based on the EQ-5D-5L. The EQ-5D-5L is a standardised, generic, self-reported HRQoL instrument recommended by most HTA agencies, including the National Institute for Health and Care Excellence.^18^ Country-specific utility scores were derived using preference-based nationally validated EQ-5D-5L value sets,^19–21^ with 3L cross-walk values for France and the UK.^22^ For trial-level analysis, we harmonised estimates using the German 5L tariffs to ensure consistent health state valuation across countries, whereas country-specific tariffs were applied in country-level analyses. QALYs were calculated over 6 months by means of linear interpolation between time points and the area under the curve calculation method. The EuroQol Visual Analogue Scale (EQ-VAS) measurements were also used. In a secondary economic analysis, broader capability-weighted life-years (CWLYs) were calculated using the standardised scores of the Oxford Capabilities Questionnaire – Mental Health (OxCAP-MH) capability well-being instrument, a mental health-oriented, self-reported outcome measure that captures broader aspects of well-being beyond HRQoL, and has shown good psychometric characteristics across different mental health disorders.^23,24^ OxCAP-MH data were collected only in Germany and the UK, because of instrument availability.

### Resource use and costs

Resource use data were obtained using the online version of the self-reported Health Economics Questionnaire (HEQ) in national languages via ePRO, with each assessment covering the period since the previous completion, consistent with the data collection schedule described above. The HEQ measures health and social care resource use (including prescribed medications), informal care and productivity losses,^25^ with detailed resource categories reported in Supplementary Table 6. Data on prescribed medications and time spent by healthcare professionals related to the PReDicT test were additionally extracted from ePRO and electronic case report forms. Intervention costs included healthcare professionals’ time for test delivery and test result review.

Mean costs per patient were calculated by multiplying resource use frequencies with relevant unit costs in Euros (€), expressed in 2018 values to align with the period of observed resource use information. In all country-level cost calculations, country-specific unit cost information from official prices, tariffs and reimbursement lists were used. Psychiatric medications were costed by multiplying daily doses with average milligram prices from national drug catalogues (Supplementary Table 2). Trial-level costs were based on a unique set of unit costs for all countries based on UK unit costs converted to Euros using the average official European Commission accounting rate for 2018,^26^ with a few additional unit costs taken directly in Euros for country-specific medications (Supplementary Table 3).

Informal care was valued using the proxy good method. Patients’ reduced household productivity was costed based on the reported level of hindrance multiplied by the average household activity time (3.15 h/day) in Europe,^27^ and valued proportionally to the average national gross hourly wage.^28^ For study participants in (self-)employment, days absent from work (absenteeism) were valued by multiplying sick leave days with the average national gross hourly wage, following the human capital approach. Days with reduced productivity while at work (presenteeism) were valued by multiplying self-reported hindered workdays by (1 **–** self-rated efficiency), then valued using the national average gross hourly wage according to the Institute of Medical Technology Assessment Productivity Cost Questionnaire’s Osterhaus method.^28^

Different HTA jurisdictions in Europe require different primary analytical perspectives for economic evaluations: (a) healthcare – Germany; (b) health and social care – UK; and (c) societal – France, The Netherlands and Spain. Therefore, costs and cost-effectiveness results were calculated and reported for all three analytical perspectives (Table 2). The health care perspective included health care costs only, the health and social care perspective included health and social care costs, and the societal perspective encompassed health and social care costs as well as informal care, household productivity losses, and productivity losses from paid work (including absenteeism and presenteeism).

### Health economic analyses

Missing outcome and cost data were imputed using multiple imputation by chained equations under the assumption that these data were missing at random with treatment group, age, gender, country and baseline values (EQ-5D for HRQoL, OxCAP-MH index for capability or costs) as predictors.^29^ The number of imputations sets was matched to the percentage of incomplete cases.^30^ The imputation of missing lost productivity costs was based on last value carried forward.

Baseline comparisons across groups were investigated using *t*-test or chi-squared test, with results reported as means with standard deviations or mean differences with 95% confidence intervals. Before-trial costs extrapolated to 24 weeks were compared with the 24-week trial period to estimate the overall cost impact of starting active primary antidepressant treatment with or without the PReDicT test. Mean incremental cost-effectiveness estimates were calculated by dividing the difference in costs with the difference in outcomes (QALY or CWLY) for the PReDicT group compared with TAU group. Multilevel mixed-effects linear regressions were used to compare differences in effects, and generalised linear models (gamma family with log link) were used for costs adjusted for treatment group, with two-sided *p*-values <0.05 considered as statistically significant. All analyses were carried out in Stata/MP version 15.1 and Microsoft Excel 365 ProPlus.

Cost-effectiveness results were expressed as incremental cost-effectiveness ratios (ICERs) and reported also as net monetary benefits (NMBs) for the full imputed data-set, using willingness-to-pay (WTP) thresholds of €34 000/QALY (corresponding to the UK’s upper £30 000/QALY threshold as of 2018) and €50 000/QALY (a commonly cited upper benchmark in European health economic evaluations).^31^ Between-group cost differences used in the cost-effectiveness analyses were based on costs accrued during the trial period. To determine uncertainty and the 95% confidence intervals of the ICERs, non-parametric bootstrapping was used. Joint distribution of the mean incremental costs and effects was illustrated on the cost-effectiveness plane consisting of four quadrants. In the south-east quadrant, PReDicT is more effective and less expensive than TAU (dominant); in the north-west quadrant, PReDicT is less effective and more expensive than TAU (dominated); in the south-west quadrant, PReDicT is less effective and less expensive than TAU; and in the north-east quadrant, PReDicT is more effective and more expensive than TAU. The probability of PReDicT being cost-effective compared with TAU depending on the society’s maximum WTP for a QALY gained was represented by cost-effectiveness acceptability curves based on the NMB approach.^32,33^

### Sensitivity analyses

Sensitivity analyses were conducted to explore the robustness of the findings across outcomes, sample compositions and cost assumptions. For outcomes, quality-of-life valuation was varied by replacing the German EQ-5D-5L tariff with the UK EQ-5D-3L cross-walk tariff at the trial level. The assumption of linear change over time was tested by modelling quality-of-life changes at the beginning of each follow-up period.

Scenario analyses explored the impact of different sample compositions. A complete-case analysis (*n* = 534) evaluated the effect of imputation by including only participants with full data at baseline, week 8 and week 24. A second scenario analysis focused on participants who reported (self-)employment during the trial (*n* = 638), to assess productivity-related effects. A third analysis included only those who completed the OxCAP-MH capabilities measure (*n* = 619), enabling evaluation of capability outcomes.

Cost-related uncertainty was addressed through alternative valuation methods and handling of outliers. Informal care was valued using an opportunity cost approach based on the average national gross hourly wage. Extreme cost outliers were identified within each group and cost category using exploratory diagnostics (including boxplots) and a conservative threshold (values exceeding three standard deviations from the mean), and were replaced with group means within each category. As an additional sensitivity analysis, costs were inflated from year 2018 to 2024, using the UK NHS Cost Inflation Index,^34^ and cost-effectiveness results were re-estimated with bootstrapping.

## Results

### Participants

A total of 913 antidepressant medication-free adults diagnosed with depression were randomised to either the PReDicT group (*n* = 460) or the TAU group (*n* = 453), with varying sample sizes across countries: France (*n* = 76), Germany (*n* = 130), The Netherlands (*n* = 54), Spain (*n* = 164), UK (*n* = 489). Table 1 presents the baseline characteristics of the study sample, which were similar between the two groups. Overall, the sample included twice as many women as men, with a mean age of 40 years, a mean length of depression of over 4 years and a baseline QIDS-SR-16 score of 15, indicating moderate to severe depression. More than half of the sample had a family history of depression and 70% (*n* = 638) were in (self-)employment at some point during the trial. The groups were well matched on baseline scores of the EQ-5D-5L, EQ-VAS or OxCAP-MH scales.

**Table 1:** Health economic analysis sample characteristics (*N* = 913)

Characteristic	PReDicT (*n* = 460)	Treatment as usual (*n* = 453)
Country, *n* (%)		
** **France	39 (8)	37 (8)
** **Germany	63 (15)	67 (14)
** **The Netherlands	28 (6)	26 (6)
** **Spain	82 (18)	82 (18)
** **UK	248 (53)	241 (54)
Mean age at baseline, years (s.d.)	38.70 (13.53)	39.21 (14.00)
Female gender, *n* (%)	285 (62)	282 (62)
Ethnicity^a^, *n* (%)		
** **White	374 (81)	374 (83)
** **Racially minoritised groups^b^	86 (19)	79 (17)
(Self-)Employed during trial, *n* (%)		
** **Yes	327 (71)	311 (69)
** **No	133 (29)	142 (31)
Working age (18–65 years)	123 (92)	132 (93)
Past retirement age (>65 years)	10 (8)	10 (7)
Mean length of education at baseline, years (s.d.)	14.14 (3.66)	14.10 (3.48)
Family history of depression, *n* (%)		
** **No	225 (49)	215 (47)
** **Yes	235 (51)	238 (53)
Mean length of depression at baseline, years (s.d.)	4.29 (7.32)	4.65 (7.49)
Mean baseline QIDS-SR-16 (s.d.)	15.67 (4.53)	15.65 (4.19)
Mean baseline MADRS (s.d.)	28.04 (7.45)	28.07 (7.08)
Mean baseline GAD-7 (s.d.)	13.59 (4.92)	13.77 (4.85)
Mean baseline SAS-SR (s.d.)	63.06 (11.16)	62.88 (11.28)
Mean baseline EQ-5D-5L index (s.d.)^c^	0.707 (0.23)	0.707 (0.20)
Mean baseline EQ-VAS (s.d.)	51.39 (20.26)	51.52 (19.94)
Mean baseline OxCAP-MH score (s.d.)^d^	60.37 (14.02)	60.16 (12.22)

QIDS-SR-16, Quick Inventory of Depressive Symptoms, 16-item self-report version (score range 0–27); MADRS, Montgomery–Åsberg Depression Rating Scale (score range 0–60); GAD-7, Generalised Anxiety Disorder Assessment, seven-item version (score range 0–21); SAS-SR, Social Adjustment Scale, self-report screener form, T-score (note a higher score indicates greater impairment, score range 38–90); EQ-VAS, EuroQol Visual Analogue Scale; OxCAP-MH, Oxford Capabilities Questionnaire – Mental Health.

a. A local ethical requirement prevented the collection of data on ethnicity from patients in France.

b. Includes American Indian or Alaska Native, Asian, Black or African American, Native Hawaiian or Other Pacific Islander, Other.

c. Based on Ludwig et al^19^.

d. Data were only collected in the UK and Germany: PReDicT (*n* = 311) and treatment as usual (*n* = 308).

### Outcome results

**Fig. 1:**
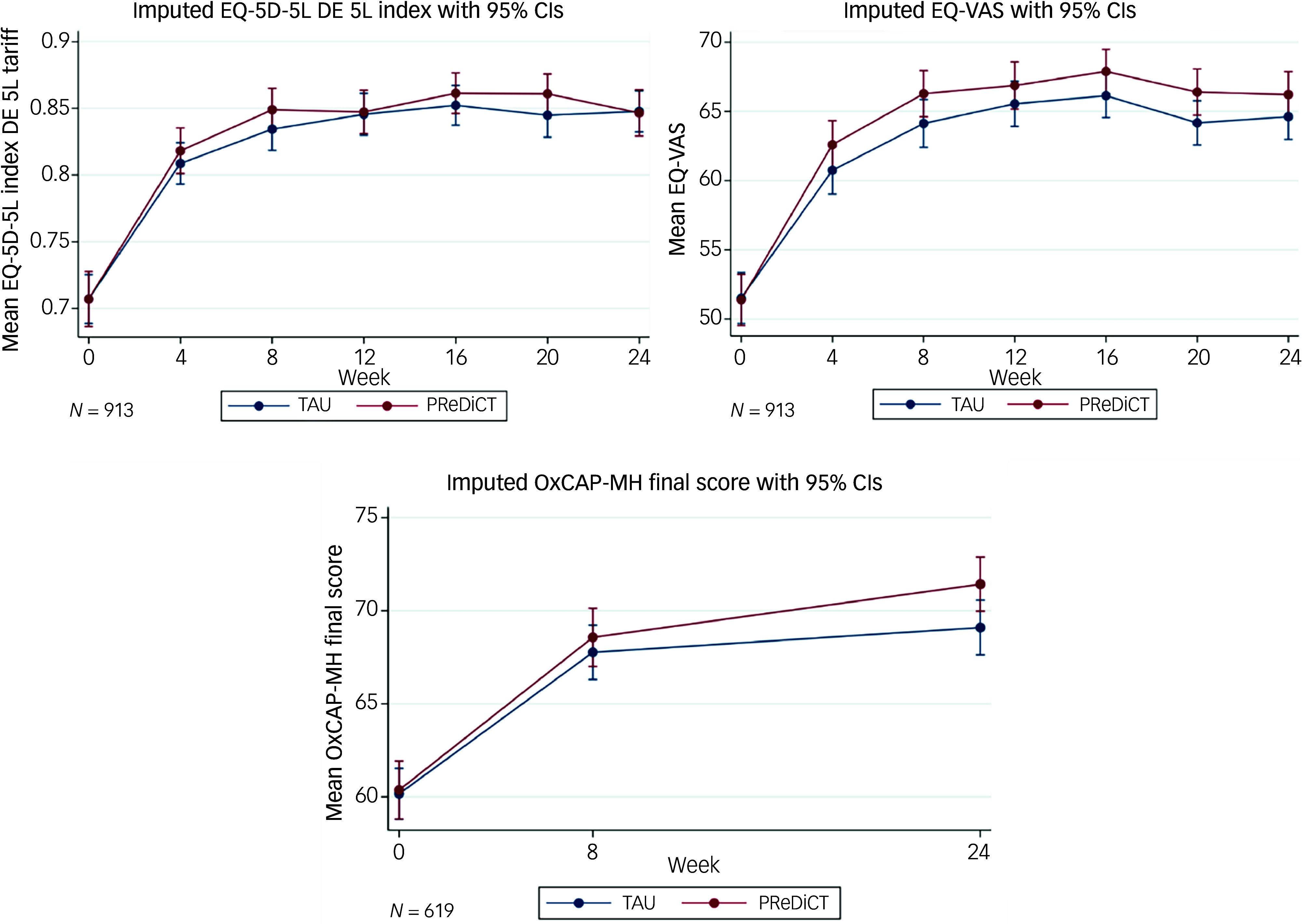
EQ-5D-5L index, EQ-VAS and OxCAP-MH scores (*n* = 913/619). EQ-5D-5L DE 5L, German EQ-5D-5L value set; EQ-VAS, EuroQol Visual Analogue Scale; OxCAP-MH, Oxford Capabilities Questionnaire – Mental Health; TAU, treatment as usual.

Average response rates to the different health economics questionnaires were 100% at baseline, declining to 84**–**86% at week 8 (OxCAP-MH: 84%, HEQ: 85%, EQ-5D: 86%) and 53–59% at week 24 (OxCAP-MH: 53%, EQ-5D: 59%, HEQ: 59%). Missingness at 48 weeks exceeded 50% across all key instruments (HEQ: 59%, EQ-5D: 59%, OxCAP-MH: 65%), with substantial variation between countries. Both groups showed significant improvements in all health economic outcomes compared with baseline. The EQ-5D-5L index increased by +0.139 (95% CI 0.120–0.159) for PReDicT and +0.140 (95% CI 0.123–0.158) for TAU, whereas OxCAP-MH scores rose by +11.056 (95% CI 9.724–12.389) for PReDicT and +8.929 (95% CI 7.713–10.146) for TAU (Fig. 1). Although there was no significant difference in EQ-5D-5L utility index between the PReDicT and the TAU groups at 24 weeks, large country variations occurred (France: **−**0.069, *p* = 0.12; Germany: +0.0323, *p* = 0.38) (Supplementary Fig. 1, Supplementary Table 5). At 24 weeks, the PReDicT group showed significantly greater improvement in capability well-being compared with the TAU group (OxCAP-MH scores: +2.127; 95% CI 0.323–3.931; *p* = 0.02; Fig. 1), corresponding to a 24% additional gain based on data from the UK and Germany (Supplementary Tables 4 and 5). There were no other significant group differences in the investigated outcomes.

### Resource use and cost results

Observed resource utilisation of health and social care services are reported in Supplementary Table 6. Total costs for each resource type are reported in Table 2. Over 24 weeks, the mean cost of the PReDicT intervention was €93 per patient. Mental healthcare costs tended to be higher in the PReDicT group (+€368, *p* = 0.08), with country-level results ranging from −€24 (The Netherlands) to +€878 (Germany); whereas non-mental healthcare costs were significantly lower in the PReDicT group (**−**€157, *p* = 0.03), with country reductions ranging from **−**€346 (France) to **−**€52 (Germany). There were no significant cost differences between the groups from any of the investigated perspectives. Mean total costs were €1009 (PReDicT) versus €799 (TAU) from the healthcare perspective, €1035 versus €826 from the health and social care perspective, and €6042 versus €5943 from the societal perspective.

**Table 2:** Mean costs per participant (€, year 2018, *N* = 913)

Cost category	During trial (week 0 to week 24)							Before trial (extrapolated for 24 weeks)						
	PReDicT (*n* = 460)		Treatment as usual (*n* = 453)					PReDicT (*n* = 460)		Treatment as usual (*n* = 453)				
	Mean	s.d.	Mean	s.d.	Mean difference	95% LCL	95% UCL	Mean	s.d.	Mean	s.d.	Mean difference	95% LCL	95% UCL
(A) Intervention (PReDicT test) costs	93.18	244.54	0.00	0.00	93.18	115.73	70.63	0.00	0.00	0.00	0.00	0.00	0.00	0.00
(B) Total medication costs	36.32	133.36	40.55	131.89	−4.23	−21.58	13.11	11.05	78.48	9.11	56.50	1.94	−6.82	10.70
Antidepressants (BNF chapter 04.03)	15.36	33.52	23.65	106.19	−8.30*	−17.42	0.83	0.32	3.04	0.64	6.65	−0.32	−0.98	0.34
Other mental health (BNF chapter 04.01/02/04)	12.71	97.02	8.31	44.67	4.40	−4.80	13.59	2.11	15.92	1.97	17.84	0.14	−2.06	2.35
Other nervous system (BNF chapter 04.05-11)	8.25	72.16	8.59	56.70	−0.33	−8.81	8.14	8.63	77.03	6.51	52.89	2.12	−6.34	10.58
(C) Mental healthcare	699.27	3388.28	420.69	2345.85	278.59	−111.60	668.77	1127.22	5961.14	903.15	2490.45	224.07	−335.51	783.64
Mental health community care	148.75	595.86	87.83	323.56	60.92**	0.02	121.82	554.44	1262.09	562.35	1327.04	−7.91	−175.86	160.04
Mental health out-patient care	83.12	665.02	94.64	596.34	−11.52	−94.85	71.81	100.47	394.69	156.67	763.57	−56.20	−131.84	19.44
Mental health in-patient care	467.40	3096.23	238.21	1973.12	229.18	−130.64	589.01	472.31	5816.54	184.14	1781.65	288.18	−226.04	802.40
A + B + C	828.77	3436.54	461.24	2361.27	367.53*	−36.66	771.73	1138.27	5961.22	912.27	2492.50	226.01	−333.70	785.72
(D) Non-mental healthcare	180.60	617.47	337.31	1559.91	−156.71**	−299.61	−13.80	789.69	2449.59	695.59	2065.62	94.09	−199.14	387.33
Primary care	16.10	55.86	13.55	39.57	2.55	−3.64	8.74	155.84	209.94	165.93	218.71	−10.09	−37.94	17.76
Non-mental health community care	17.04	98.66	22.04	97.61	−5.01	−18.21	8.20	161.64	758.37	81.75	358.99	79.89**	4.53	155.25
Non-mental health out-patient care	49.26	256.96	70.70	312.01	−21.44	−59.71	16.83	218.61	562.21	254.85	913.34	−36.24	−132.26	59.78
Non-mental health in-patient care	98.20	488.27	231.01	1461.65	−132.81**	−264.09	−1.53	253.59	1984.26	193.05	1621.03	60.54	−176.44	297.52
Perspective 1: A + B + C + D (healthcare)	1009.37	3512.19	798.54	3185.88	210.83	−230.53	652.18	1927.96	6491.86	1607.86	3544.63	320.10	−335.97	976.17
(E) Social care	25.94	129.71	27.17	230.23	−1.23	−25.16	22.71	36.43	294.62	45.98	416.74	−9.55	−55.79	36.70
Perspective 2: A + B + C + D + E (health and social care)	1035.31	3530.02	825.71	3207.87	209.60	−233.83	653.03	1964.39	6510.75	1653.84	3563.09	310.55	−348.62	969.72
(F) Indirect costs	5006.36	6172.54	5116.77	6295.73	−110.41	−919.67	698.84	6665.89	7489.37	6890.52	9190.81	−224.63	−1308.88	859.62
Informal care	391.60	1498.23	410.76	1928.98	−19.16	−242.02	203.70	634.08	2223.73	787.10	6178.10	−153.02	−715.71	409.67
Transport expenses	3.80	35.92	1.53	6.31	2.26	−0.48	5.00	14.45	58.57	12.68	38.80	1.77	−4.57	8.11
Lost household productivity	2130.42	1839.73	2132.10	1851.10	−1.68	−241.09	237.74	2587.14	1820.76	2407.47	1781.33	179.67	−54.25	413.59
Lost productivity	2480.54	5084.51	2572.38	4878.29	−91.84	−739.43	555.76	3430.22	6157.83	3683.27	5908.91	−253.05	−1039.79	533.69
Absenteeism	1988.09	4563.59	2114.74	4518.76	−126.65	−717.41	464.11	2173.36	4952.98	2278.42	4980.21	−105.05	−750.26	540.15
Presenteeism	492.45	1607.56	457.63	1483.06	34.81	−165.74	235.37	1256.85	2618.34	1404.85	2726.78	−148.00	−496.40	200.41
Perspective 3: A + B + C + D + E + F (societal)	6041.67	7712.42	5942.48	7381.13	99.19	−880.30	1078.67	8630.28	10 676.21	8544.35	10 000.77	85.92	−1256.01	1427.86

LCL, lower confidence level; UCL, upper confidence level; BNF, British National Formulary.

Mean difference is mean(PReDicT) – mean(treatment as usual). GBP prices converted to EUR using average monthly exchange rate GBP/EUR of 1.13, retrieved from https://ec.europa.eu/budget/graphs/inforeuro.html (2018).

*p < 0.1; **p < 0.05.

At trial level, the mean number of antidepressant medication taken was similar between the groups (PReDicT: 1.17, TAU: 1.20) with considerable country-level variations (range: France PReDicT 1.03, TAU 1.11; Germany PReDicT 1.40, TAU 1.37) (Supplementary Table 7a). Over 80% of the participants used only one type of antidepressant during the trial period (Supplementary Table 7b). The PReDicT group tended to have fewer antidepressant medication days, resulting in lower antidepressant costs (**−**€8, *p* = 0.08), but with major country variations (range: from **−**€31 in Germany to +€8 in The Netherlands) (Table 2, Supplementary Tables 8 **–** 12).

On the other hand, compared with the before-trial period, both groups showed significant per-patient cost reductions during the trial (health and social care perspective: PReDicT **−**€929, TAU **−**€828; societal perspective: PReDicT **−**€2,589, TAU **−**€2,602) (Table 3).

**Table 3:** Mean cost differences per participant between the before- and during-trial periods (€, year 2018, *N* = 913)

Cost category	Before trial versus during trial costs							
	PReDicT (*n* = 460)				Treatment as usual (*n* = 453)			
	Mean difference		95% LCL	95% UCL	Mean difference		95% LCL	95% UCL
(A) Intervention (PReDicT test) costs	93.18	***	115.73	70.63	0.00		0.00	0.00
(B) Total medication costs	25.26	***	14.76	35.77	31.44	***	20.26	42.61
Antidepressants (BNF chapter 04.03)	15.04	***	11.97	18.11	23.01	***	13.22	32.81
Other mental health (BNF chapter 04.01/02/04)	10.60	**	1.83	19.37	6.34	**	2.59	10.10
Other nervous system (BNF chapter 04.05-11)	−0.37		−2.44	1.70	2.08	*	−0.24	4.41
(C) Mental healthcare	−427.95		−1044.25	188.35	−482.47	**	−771.10	−193.84
Mental health community care	−405.69	***	−520.57	−290.80	−474.52	***	−596.65	−352.38
Mental health out-patient care	−17.35		−83.89	49.20	−62.03	*	−135.79	11.73
Mental health in-patient care	−4.92		−603.44	593.60	54.07		−179.11	287.26
A + B + C	−309.50		−927.13	308.12	−451.03	***	−739.57	−162.49
(D) Non-mental healthcare	−609.09	***	−834.76	−383.41	−358.28	***	−576.83	−139.73
Primary care	−139.74	***	−158.18	−121.31	−152.38	***	−172.48	−132.28
Non-mental health community care	−144.60	***	−213.72	−75.49	−59.71	***	−92.82	−26.60
Non-mental health out-patient care	−169.35	***	−224.67	−114.03	−184.15	***	−270.66	−97.64
Non-mental health in-patient care	−155.39		−339.41	28.63	37.96		−156.68	232.61
Perspective 1: A + B + C + D (healthcare)	−918.59	***	−1571.19	−265.99	−809.31	***	−1217.94	−400.69
(E) Social care	−10.49		−37.78	16.81	−18.81		−62.57	24.96
Perspective 2: A + B + C + D + E (health and social care)	−929.08	***	−1583.26	−274.90	−828.12	***	−1240.00	−416.25
(F) Indirect costs	−1659.53	***	−2209.51	−1109.56	−1773.75	***	−2572.21	−975.29
Informal care	−242.48	**	−444.10	−40.86	−376.34		−961.46	208.78
Transport expenses	−10.65	***	−15.28	−6.03	−11.14	***	−14.77	−7.52
Lost household productivity	−456.72	***	−596.85	−316.59	−275.37	***	−416.99	−133.76
Lost productivity	−949.68	***	−1395.03	−504.32	−1110.89	***	−1574.73	−647.05
Absenteeism	−185.27	***	−575.84	205.30	−163.67		−586.75	259.40
Presenteeism	−764.41	***	−975.97	−552.85	−947.22	***	−1163.88	−730.56
Perspective 3: A + B + C + D + E + F (societal)	−2588.61	***	−3464.90	−1712.31	−2601.87	***	−3529.85	−1673.89

LCL, lower confidence level; UCL, upper confidence level; BNF, British National Formulary.

Mean difference is mean(during trial costs) – mean(before trial costs).

*p < 0.1; **p < 0.05; ***p < 0.01.

### Cost-effectiveness results

Table 4 summarises the main cost-effectiveness results. We found no significant differences in either QALYs or CWLYs between the PReDicT and TAU groups over the 24-week analysis period.

**Table 4:** Cost-effectiveness of PReDicT from different analytical perspectives

Outcome	*n*	Cost difference^a^ (95% CI)	Outcome difference^a^ (95% CI)	ICER (95% CI)	CE plane, %			
					NE	SE	SW	NW
1: Healthcare perspective								
** **HRQoL (EQ-5D-5L)	913	€210.83 (−€230.53 to €652.18)	0.0039 (−0.0051 to 0.0128)	€54 630/QALY (−€700 060/QALY to €438 894/QALY)	45	24	9	22
** **Capability (OxCAP-MH)	619	€376.73 (−€122.25 to €875.71)	0.0056 (−0.0034 to 0.0146)	€67 266/CWLY (−€385 130/CWLY to €683 589/CWLY)	84	5	1	10
2: Health and social care perspective								
** **HRQoL (EQ-5D-5L)	913	€209.60 (−€233.83 to €653.03)	0.0039 (−0.0051 to 0.0128)	€54 312/QALY (−€744 165/QALY to €480 743/QALY)	42	24	11	23
** **Capability (OxCAP-MH)	619	€392.51 (−€108.79 to €893.81)	0.0056 (−0.0034 to 0.0146)	€70 083/CWLY (−€624 161/CWLY to €737 264/CWLY)	86	4	0	10
3: Societal perspective								
** **HRQoL (EQ-5D-5L)	913	€99.19 (−€880.30 to €1078.67)	0.0039 (−0.0051 to 0.0128)	€25 701/QALY (−€1 193 736/QALY to €1 005 554/QALY)	31	40	10	19
** **Capability (OxCAP-MH)	619	€658.9 (−€463.85 to €1781.65)	0.0056 (−0.0034 to 0.0146)	€117 648/CWLY (−€1 248 784/CWLY to €1 226 899/CWLY)	75	14	1	10

ICER, incremental cost-effectiveness ratio; CE, cost-effective; NE, north-east quadrant; SE, south-east quadrant; SW, south-west quadrant; NW, north-west quadrant; HRQoL, health-related quality of life; QALYs, quality-adjusted life-years; OxCAP-MH, Oxford Capabilities Questionnaire – Mental Health; CWLY, capability-weighted life-years.

a. Difference is mean(PReDicT) - mean(treatment as usual); multiple imputation included treatment group, age, gender, baseline values (EQ-5D for HRQoL, OxCAP-MH index for capability, or baseline cost) as predictors; differences were estimated using regression models with treatment group as the explanatory variable.

From the healthcare perspective, the mean ICER was €54 630/QALY; from the health and social care perspective, the mean ICER was €54 312/QALY; and from the societal perspective, the mean ICER was €25 701/QALY. CWLY-based estimates were higher for all three perspectives.

**Fig. 2:**
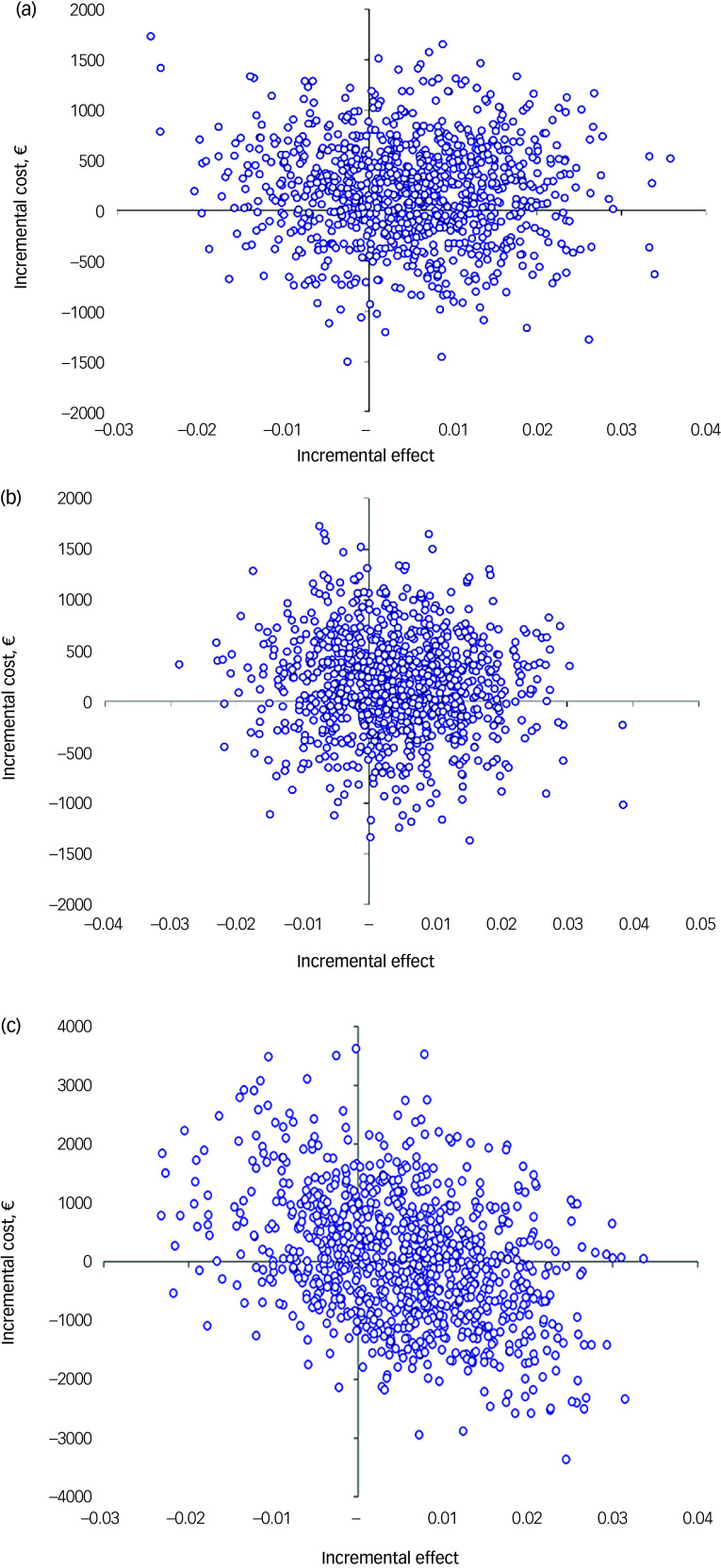
Cost-effectiveness uncertainty by analytical perspective (€, year 2018, *N* = 913). (a) Healthcare perspective (1), (b) health and social care perspective (2), (c) societal perspective (3).

Cost-effectiveness estimates were distributed across all four quadrants of the cost-effectiveness plane, indicating major uncertainty in the results both for QALYs and CWLYs from all perspectives (Fig. 2). For QALYs, 45% (healthcare perspective), 42% (health and social care perspective) and 31% (societal perspective) of the simulations fell in the north-east quadrant, indicating better outcomes with higher costs, whereas for CWLY-based estimates, the same proportions were 84, 86 and 75%, respectively (Table 4).

**Fig. 3:**
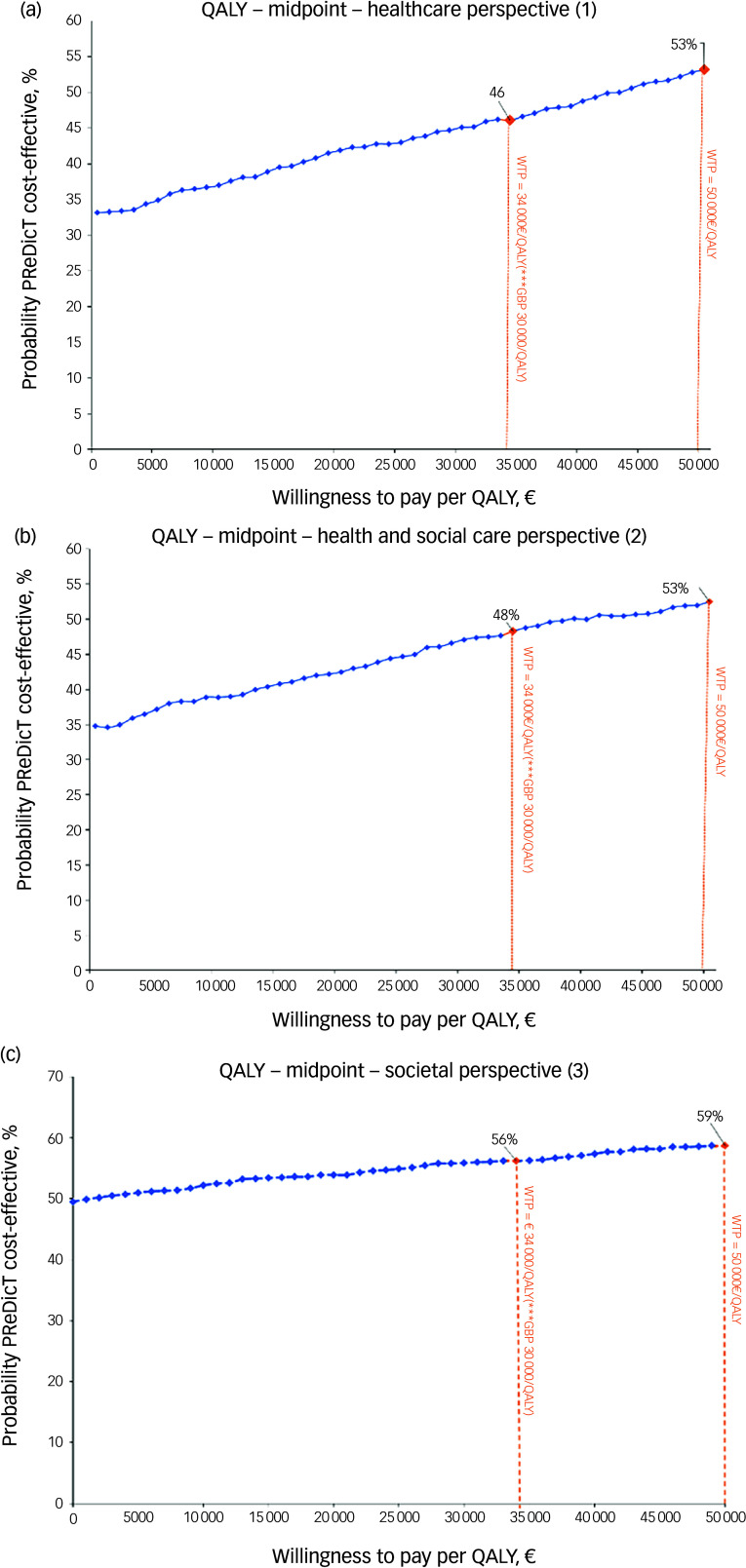
Cost-effectiveness acceptability curves by analytical perspective (€, year 2018, *N* = 913). (a) healthcare perspective (1), (b) health and social care perspective (2), (c) societal perspective (3). QALY, quality-adjusted life-year; WTP, willingness to pay.

At a tentative ‘European’ WTP threshold of €50 000/QALY, the probability of PReDicT being cost-effective at the trial level was 53% with a NMB of €43 from the healthcare perspective; 53% with a NMB of €27 from the health and social care perspective; and 59% with a NMB of €237 from the societal perspective (Fig. 3, Supplementary Table 13). At a UK threshold of £30 000/QALY (€34 000) for the UK subsample, these probabilities were 82% with a NMB of €227, 81% with a NMB of €216, and 71% with a NMB of €388, respectively (Supplementary Table 14, Supplementary Fig. 2).

### Sensitivity analysis results

Sensitivity analyses supported the robustness of the main findings across outcomes, sample compositions and cost assumptions. Testing alternative HRQoL valuation methods, using the UK 3L cross-walk tariff instead of the German 5L tariff at trial level, did not meaningfully alter the results (Supplementary Table 4). Similarly, modelling HRQoL changes at the beginning rather than the midpoint of each time period had no significant impact (Supplementary Tables 15 and 16).

Scenario analyses based on different sample compositions showed consistent findings. The complete-case analysis including only participants with full data at all time points showed no significant differences between groups over 24 weeks (Supplementary Tables 17 and 18). In the employed subgroup (*n* = 638), results from the societal perspective indicated a non-significant cost reduction of **−**€106 (95% CI **−**€1474 to €1261) for PReDicT compared with TAU (Supplementary Table 19). Most simulations fell within the north-east (32%) and south-east (57%) quadrants (Supplementary Table 20). At a WTP threshold of €50 000, the probability of PReDicT being cost-effective increased to 71%, compared with 59% in the fully imputed analysis (Supplementary Table 21). Cost results for the OxCAP-MH sample (*n* = 619) were consistent with the main analysis (Supplementary Table 22).

Cost-related sensitivity analyses also showed limited impact. Valuing informal care using either the opportunity cost method or the replacement cost approach produced comparable results. Adjusting for outliers indicated a trend toward higher costs in the PReDicT group from the health and social care perspective (+€240.05, *p* = 0.09) because of higher mental healthcare costs (+€245.78, *p* = 0.08), and non-mental healthcare cost-savings were no longer significant (Supplementary Table 23). Inflating costs to year 2024 increased absolute cost estimates (and therefore ICER point estimates) as expected, but without changing the overall interpretation of the cost-effectiveness results (Supplementary Table 24).

## Discussion

This study assessed the costs, HRQoL and capability well-being outcomes and cost-effectiveness associated with personalised antidepressant medication optimised using a digital tool, the PReDicT test, compared with TAU. Cost-effectiveness was assessed across five European countries and three analytical perspectives over a 24-week follow-up period within the PReDicT trial.

Both groups significantly improved in all investigated outcomes at levels beyond those expected from standard care. For example, in the earlier STAR*D trial, many patients continued to experience impaired quality of life despite symptom remission, highlighting the disconnection between symptom reduction and functional recovery.^35^ This suggests that regular monitoring of patients on antidepressant treatment, as implemented in the PReDicT trial, may enhance patient outcomes and reduce costs. Notably, the PReDicT test led to significantly greater improvements in self-rated capability well-being (OxCAP-MH) in the UK and Germany, aligning with significant improvements in social functioning at 24 weeks.^15^ Qualitative findings suggest that receiving feedback encouraged patients to actively self-manage their depression, potentially enhancing these effects.^16^ Structured feedback is a key component of measurement-based depression care, which can improve functional outcomes and may be cost-effective; our findings provide additional support. However, the primary outcome did not differ between groups, and benefits were not consistent across countries, so some improvements may reflect regression to the mean or non-specific trial effects. No significant differences were observed in QALYs or CWLYs.

The PReDicT test did not increase antidepressant or other nervous system medication costs compared with TAU, although costs for other mental health medications were higher. Furthermore, we found that the average intervention cost of €93 per patient was partially offset by reductions in non-mental healthcare costs and some indirect costs, such as productivity losses. These patterns align with established evidence from the UK about the economic benefits of preventing and alleviating depression in working-age adults, particularly through reduced absenteeism,^36^ and recent findings about the excess physical comorbidity burden and related hospital costs of depression.^2,7^

The economic analysis did not provide conclusive evidence about the cost-effectiveness of the PReDicT test. Most simulations fell in the north-east quadrant, representing higher costs and better outcomes. At a ‘European’ threshold of €50 000/QALY, the 53% probability of cost-effectiveness from the health and social care perspective increased only to 59% probability when societal costs were also considered. Although broader perspectives include additional cost components, this does not necessarily increase incremental costs or worsen ICERs. In this study, productivity and informal care costs partially offset health and social care cost differences. This perspective-dependent variability underscores the importance of cross-sectoral reimbursement models for digital mental health technologies. Results remained robust across sensitivity analyses, with societal cost-effectiveness being higher among individuals who were (self-)employed. Because of sample size limitations and the availability of explicit national cost-effectiveness thresholds, we conducted full country-specific cost-effectiveness analysis only for the UK. This showed that the probability of cost-effectiveness from the health and social care perspective was over 80% in the UK. These findings highlight potential country-specific differences in depression care pathways and resource use, emphasising the need for context-tailored market access strategies. Overall, the present evaluation confirms prior suggestions that the PReDicT test’s primary economic benefits may lie in improved functioning, leading to enhanced capabilities and productivity, rather than healthcare cost savings.^13^ A further sensitivity analysis where costs were inflated to 2024 prices increased the ICERs as expected, but did not alter the conclusions regarding cost-effectiveness and related uncertainty.

There were several key strengths and limitations of the economic evaluation. We report cost-effectiveness results for multiple outcomes, perspectives and aggregation levels, also including societal cost information. Despite several cross-country implementation challenges, the PReDicT trial economic evaluation achieved complete baseline data and maintained over 50% follow-up response rates at 24 weeks, complemented by robust missing data handling strategy. Furthermore, we explored different methodological approaches that allowed detailed impact assessment of some underlying country-level variations, which are comprehensively reported. The multinational design itself serves as an important strength, offering valuable insights into how the intervention performed across diverse healthcare systems, providing early evidence on potential variability in real-world implementation.

On the other hand, the multinational nature of the economic analysis posed relevant challenges and limitations. The observed substantial between-country variations in follow-up completion rates, outcomes and costs likely reflect fundamental differences in healthcare systems and depression care practices. These variations likely increased uncertainty in the overall cost-effectiveness results beyond those resulting from the substantial heterogeneity usually present in cost-effectiveness analysis because of individual cost variations, and may limit the generalisability of the overall trial-level estimates.^37^ In addition, trial-level unit costs were drawn from the UK driven by considerations such as country-specific sample size and unit cost information completeness, reflecting a trade-off between internal consistency of trial-level costing and full cross-country price comparability. Third, because of reliance on self-reported economic data, we cannot exclude potential recall bias, particularly for informal care costs and productivity effects. Fourth, as the trial was powered for clinical rather than economic end-points, some analyses had limited precision to detect smaller but potentially meaningful cost differences, and did not have sufficient sample size to make robust inference about country-specific cost-effectiveness results. For this reason, we only report these for the UK. To further mitigate some of these limitations, we implemented extensive sensitivity analyses testing alternative assumptions about cost calculation and missing data, resulting in consistent findings across analytical scenarios. However, missingness in health economic data at 48 weeks was substantial (>50% across instruments and heterogeneous by country), precluding a valid longer-horizon cost-effectiveness analysis. Nevertheless, country-specific care variations could not be adjusted for methodologically. Neither could we account for the methodological differences in country-specific unit cost variations, or use a validated supra-national index for the EQ-5D for fully harmonised trial-level estimates. Finally, although clinicians and patients in the TAU group did not receive any of the PReDicT test or QIDS-SR-16 results, completing these on a regular basis as part of trial participation could heighten patients’ awareness beyond standard care, and likely also lead to better outcomes in the TAU group.

### Implications

The current study suggests potentially meaningful economic benefits of actively monitoring antidepressant therapy as implemented in the PReDicT trial, both for the healthcare systems and the society. However, without a waitlist control, we cannot determine what proportion of cost reductions stem from trial participation effects versus active monitoring including the PReDicT test. Although the PReDicT test showed some promising effects on functional recovery and anxiety symptoms, these effects were modest and not consistently statistically significant, highlighting considerable uncertainty in both clinical and economic outcomes.^15^ This underscores the need for further research to refine the predictive algorithm and clarify its potential value in guiding personalised antidepressant treatment. Nevertheless, the study highlights possibilities for substantial cost savings through remote patient monitoring, warranting further health services research. Additionally, the trial reinforced the need for harmonised multinational outcome and costing tools, such as those recently developed in the European PECUNIA project.^38–40^

## Supporting information

10.1192/bjo.2026.11021.sm001 Perić et al. supplementary material Perić et al. supplementary material

## Data Availability

The data that support the findings of this study are available on request from the sponsor of the study, P1Vital (info@p1vital.com). The data are not publicly available due to their containing information that could compromise the privacy of research participants. The study sponsor will retain ultimate ownership of the study data.

## Supplementary material

The supplementary material is available online at https://doi.org/10.1192/bjo.2026.11021
