# Supplementary material for: The cost-effectiveness of predictive algorithm guided primary antidepressant treatment: economic evaluation of the multinational PReDicT randomised controlled trial
Source: BJPsych Open. 2026 Apr 13;12(3):e107. doi: 10.1192/bjo.2026.11021 (PMC13107311; doi:10.1192/bjo.2026.11021)
Supplement: Perić et al. supplementary material [file S2056472426110217sup001.docx]

Supplementary materials

**The cost-effectiveness of predictive algorithm guided primary antidepressant treatment: economic evaluation of the multi-national PReDicT randomized controlled trial**

[Tables 3](#_Toc203682109)

[Supplementary Table S1: CHEERS 2022 checklist 3](#_Toc203682110)

[Supplementary Table S2: Country-level unit costs (€, year 2018) 5](#_Toc203682111)

[Supplementary Table S3: Trial-level unit costs (€, year 2018) 10](#_Toc203682112)

[Supplementary Table S4: Health economic outcomes over time 12](#_Toc203682113)

[Supplementary Table S5: Health economic outcomes over 24 weeks by country 13](#_Toc203682114)

[Supplementary Table S6: Observed health and social care resource use 14](#_Toc203682115)

[Supplementary Table S7a: Average number of antidepressants taken per participant 15](#_Toc203682116)

[Supplementary Table S7b: Number of different types of antidepressants taken by participants 15](#_Toc203682117)

[Supplementary Table S8: Mean costs per participant - France (€, year 2018, n=76) 16](#_Toc203682118)

[Supplementary Table S9: Mean costs per participant - Germany (€, year 2018, n=130) 17](#_Toc203682119)

[Supplementary Table S10: Mean costs per participant - Netherlands (€, year 2018, n=54) 18](#_Toc203682120)

[Supplementary Table S11: Mean costs per participant - Spain (€, year 2018, n=164) 19](#_Toc203682121)

[Supplementary Table S12: Mean costs per participant - United Kingdom (€, year 2018, n=489) 20](#_Toc203682122)

[Supplementary Table S13: Net monetary benefit of PReDicT in comparison to TAU (n=913) 21](#_Toc203682123)

[Supplementary Table S14: Cost-effectiveness of PReDicT from different analytical perspectives - United Kingdom (€, year 2018, n=489) 22](#_Toc203682124)

[Supplementary Table S15: QALY and CWLY under alternative timepoint assumptions 23](#_Toc203682125)

[Supplementary Table S16: Cost-effectiveness of PReDicT from different analytical perspectives, assuming outcome changes at the start of each timepoint 24](#_Toc203682126)

[Supplementary Table S17: Health economic outcomes over time - complete cases (n=534) 25](#_Toc203682127)

[Supplementary Table S18a: Health economic analysis sample characteristics– complete cases (n=534) 26](#_Toc203682128)

[Supplementary Table S18b: Mean costs per participant - complete cases (€, year 2018, n=534) 27](#_Toc203682129)

[Supplementary Table S18c: Mean cost differences per participant between the before- and during-trial periods - complete cases (€, year 2018, n=534) 28](#_Toc203682130)

[Supplementary Table S19a: Health economic analysis sample characteristics at baseline - employed sample (n=638) 29](#_Toc203682131)

[Supplementary Table S19b: Mean costs per participant - employed sample (€, year 2018, n=638) 30](#_Toc203682132)

[Supplementary Table S20: Cost-effectiveness of PReDicT by analytical perspective - employed sample (€, year 2018, n=638) 31](#_Toc203682133)

[Supplementary Table S21: Net monetary benefit of PReDicT in comparison to TAU - employed sample (€, year 2018, n=638) 32](#_Toc203682134)

[Supplementary Table S22a: Health economic analysis sample characteristics at baseline – OxCAP-MH sample (n=619) 33](#_Toc203682135)

[Supplementary Table S22b: Mean costs per participant – OxCAP-MH sample (€, year 2018, n=619) 34](#_Toc203682136)

[Supplementary Table S23: Outlier-adjusted mean costs per participant (€, year 2018, n=913) 35](#_Toc203682137)

[Figures 36](#_Toc203682138)

[Supplementary Figure S1 36](#_Toc203682139)

[Supplementary Figure S2 37](#_Toc203682140)

[References 38](#_Toc203682141)

# Tables

## Supplementary Table S1: CHEERS 2022 checklist

| **Topic** | **No.** | **Item** | **Reported in** |  |
| --- | --- | --- | --- | --- |
| Title | 1 | Identify the study as an economic evaluation and specify the interventions being compared. | Title, page 1, lines 1-3 |  |
| Abstract | 2 | Provide a structured summary that highlights context, key methods, results, and alternative analyses. | Abstract: entire section |  |
| **Introduction** | | | | |
| Background and objectives | 3 | Give the context for the study, the study question, and its practical relevance for decision making in policy or practice. | Background: 1st paragraph (Lines 44-76); Aims: Standalone section (Lines 77-80) |  |
| **Methods** | | | | |
| Health economic analysis plan | 4 | Indicate whether a health economic analysis plan was developed and where available. | Methods: lines 105-106 |  |
| Study population | 5 | Describe characteristics of the study population (such as age range, demographics, socioeconomic, or clinical characteristics). | Method: 2nd paragraph |  |
| Setting and location | 6 | Provide relevant contextual information that may influence findings. | Background: Lines 69-76; Method: 1st paragraph |  |
| Comparators | 7 | Describe the interventions or strategies being compared and why chosen. | Background: Lines 56-67; Method: 2nd paragraph |  |
| Perspective | 8 | State the perspective(s) adopted by the study and why chosen. | Method: 3rd paragraph, resource use and costs: 4^th^ paragraph |  |
| Time horizon | 9 | State the time horizon for the study and why appropriate. | Method: 3rd paragraph |  |
| Discount rate | 10 | Report the discount rate(s) and reason chosen. | Not applicable (24-week horizon) |  |
| Selection of outcomes | 11 | Describe what outcomes were used as the measure(s) of benefit(s) and harm(s). | Method/Outcomes: 1st paragraph |  |
| Measurement of outcomes | 12 | Describe how outcomes used to capture benefit(s) and harm(s) were measured. | Method: 2nd paragraph |  |
| Valuation of outcomes | 13 | Describe the population and methods used to measure and value outcomes. | Method/Outcomes: 1st paragraph |  |
| Measurement and valuation of resources and costs | 14 | Describe how costs were valued. | Method/Resource use: 1st-3rd paragraphs |  |
| Currency, price date, and conversion | 15 | Report the dates of the estimated resource quantities and unit costs, plus the currency and year of conversion. | Method: 2nd paragraph |  |
| Rationale and description of model | 16 | If modelling is used, describe in detail and why used. Report if the model is publicly available and where it can be accessed. | Not applicable (within-trial analysis) |  |
| Analytics and assumptions | 17 | Describe any methods for analysing or statistically transforming data, any extrapolation methods, and approaches for validating any model used. | Method/Health economic analyses: 1st -4^th^ paragraph; Sensitivity analyses: 1st -2^nd^ paragraph |  |
| Characterising heterogeneity | 18 | Describe any methods used for estimating how the results of the study vary for subgroups. | Methods/Health economic analyses: 1st- 2nd paragraph (country variations) |  |
| Characterising distributional effects | 19 | Describe how impacts are distributed across different individuals or adjustments made to reflect priority populations. | Not reported |  |
| Characterising uncertainty | 20 | Describe methods to characterise any sources of uncertainty in the analysis. | Method/Health economic analyses: 3^rd^ paragraph |  |
| Approach to engagement with patients and others affected by the study | 21 | Describe any approaches to engage patients or service recipients, the general public, communities, or stakeholders (such as clinicians or payers) in the design of the study. | Not applicable |  |
| **Results** | | | | |
| Study parameters | 22 | Report all analytic inputs (such as values, ranges, references) including uncertainty or distributional assumptions. | Results/Participants: 1^st^ paragraph and Table 1, Supplementary Tables S2, S3, S18a, S19a, S22a |  |
| Summary of main results | 23 | Report the mean values for the main categories of costs and outcomes of interest and summarise them in the most appropriate overall measure. | Results/Outcome results, Resource use and cost results, Cost-effectiveness results, Tables 2-4, Supplementary Tables S4, S5, S8-S14, S19, S20; Figures S1-S2 |  |
| Effect of uncertainty | 24 | Describe how uncertainty about analytic judgments, inputs, or projections affect findings. Report the effect of choice of discount rate and time horizon, if applicable. | Results/Cost-effectiveness results: 2^nd^ paragraph, Sensitivity analysis results; Supplementary Tables S15-S23 |  |
| Effect of engagement with patients and others affected by the study | 25 | Report on any difference patient/service recipient, general public, community, or stakeholder involvement made to the approach or findings of the study | Not reported |  |
| **Discussion** | | | | |
| Study findings, limitations, generalisability, and current knowledge | 26 | Report key findings, limitations, ethical or equity considerations not captured, and how these could affect patients, policy, or practice. | Discussion |  |
| **Other relevant information** | | | | |
| Source of funding | 27 | Describe how the study was funded and any role of the funder in the identification, design, conduct, and reporting of the analysis | End of manuscript |  |
| Conflicts of interest | 28 | Report authors conflicts of interest according to journal or International Committee of Medical Journal Editors requirements. | End of manuscript |  |

*From:* Husereau D, Drummond M, Augustovski F, et al. Consolidated Health Economic Evaluation Reporting Standards 2022 (CHEERS 2022) Explanation and Elaboration: A Report of the ISPOR CHEERS II Good Practices Task Force. Value Health 2022;25. <doi:10.1016/j.jval.2021.10.008>

## Supplementary Table S2: Country-level unit costs (€, year 2018)

|  |  | FRANCE | | GERMANY | | UNITED KINGDOM | | | NETHERLANDS | | | | SPAIN | |  |  |
| --- | --- | --- | --- | --- | --- | --- | --- | --- | --- | --- | --- | --- | --- | --- | --- | --- |
|  | **Unit** | **Cost in €** | **Source** | **Cost in €** | **Source** | **Cost in £** | **Cost in €** | **Source** | **Cost in €** | | **Source** | | **Cost in €** | | | **Source** |
| Medication |  |  |  |  |  |  |  |  |  | |  | |  | | |  |
| Oral medication | per mg / mL | **various** | **(1)** | **various** | **(2)** | **various** | | BNF 76 (September 2018 – March 2019) (3) | **various** | | **(4)** | | **various** | | | **(5)** |
| Mental Health (MH) Care | | |  |  |  |  |  |  |  | |  | |  | | |  |
| MH Community Care | |  |  |  |  |  |  |  |  | |  | |  | | |  |
| Community mental health centre | per attendance | 51.30 | Rapp et al. (2018)* (6) | 49.53 | Bock et al. (2015) * (7) | 45.00 | 50.83 | Curtis & Burns (2018) (8) | 31.51 | | Goorden et al. (2017)* (9) | | 32.59 | | | Departament de salut (2018) (10) |
| Day care centre (groups/programs, non-health care staff) | per attendance | 41.79 | Curtis & Burns (2018) (8) | 8.42 | Grupp et al. (2017) (11) | 37.00 | 41.79 | Curtis & Burns (2018) (8) | 66.12 | | Hakkaart-van Roijen et al. (2016)* (12) | | 55.25 | | | Obiluke Consulting (2019)*  (13) |
| Group therapy | per attendance | 18.07 | Curtis & Burns (2018) (8) | 26.07 | Grupp et al. (2017) (11) | 16.00 | 18.07 | Curtis & Burns (2018) (8) | 60.87 | | Hakkaart-van Roijen et al. (2016)* (12) | | 20.89 | | | Fernández et al. (2018)* (14) |
| Sheltered workshop | per attendance | 193.00 | L’Etablissement Public de Santé Maison Blanche (2019) (15) | 63.62 | Grupp et al. (2017) (11) | 112.99 | 127.61 | Mavranezouli (2014)* (16) | 127.61 | | | Mavranezouli (2014)* (16) | | |  |  |
| Specialist education | per attendance | n.r. |  | 47.02 | Grupp et al. (2017) (11) | 102.21 | 115.44 | Curtis (2014)* (17) | n.r. | |  | | 47.02 | | | Grupp et al. (2017) (11) |
| Self Help/Support Group | per attendance | 58.96 | Goorden et al. (2017)* (9) | 58.96 | Goorden et al. (2017)* (9) | 96.00 | 108.43 | NHS reference costs 2017-18 (18) | 58.96 | | Goorden et al. (2017)* (9) | | 35.61 | | | Departament de salut (2018) (10) |
| Community Psychiatrist | per contact | 51.48 | L’Assurance Maladie-AMELI (2019) (19) | 47.45 | Bock et al. (2015) * (7) | 46.00 | 51.96 | NHS reference costs 2017-18 (18) | 92.77 | | Kanters et al. (2017) * (20) | | 49.12 | | | Salvador-Carulla et al. (2011)* (21) |
| Community Psychologist | per contact | 51.30 | Rapp et al. (2018)* (6) | 82.85 | Bock et al. (2015) * (7) | 146.00 | 164.90 | NHS reference costs 2017-18 (18) | 77.97 | | Kanters et al. (2017) * (20) | | 65.78 | | | Salvador-Carulla et al. (2011)* (21) |
| CPN (psychiatric nurse/case manager) | per contact | 32.34 | Grupp et al. (2017) (11) | 32.34 | Grupp et al. (2017) (11) | 13.25 | 14.97 | Curtis & Burns (2018) (8) | 83.36 | | Goorden et al. (2017)* (9) | | 25.46 | | | Obiluke Consulting (2019)*  (13) |
| Other MH community-based day services | per attendance | 51.48 – 60.39 | L’Assurance Maladie-AMELI (2019) (19), Average market price (22) | 43.70 – 82.85 | Bock et al. (2015) * (7), Average market price (23) | 54.76 – 194.69 | 61.85 – 219.90 | NHS reference costs 2017-18 (18), Curtis & Burns (2017)* (24), Curtis & Burns (2018) (8), Email enquiry | 33.56 – 84.22 | | Kanters et al. (2017) * (20) | | 43.62 – 135.39 | | | Consejería de Sanidad (2017)* (25), Average market prices (26, 27) |
| MH Outpatient Care | |  |  |  |  |  |  |  |  | |  | |  | | |  |
| Psychiatric outpatient | per visit | 51.48 | L’Assurance Maladie-AMELI (2019) (19) | 73.14 | Grupp et al. (2017) (11, 28) | 103.00 | 116.34 | NHS reference costs 2017-18 (18) | 96.72 | | Kanters et al. (2017) * (20) | | 49.12 | | | Salvador-Carulla et al. (2011)* (21) |
| Day hospital (group therapies, etc.) | per visit | 313.00 | Hospital Sainte-Anne (2018) (29) | 234.30 | Bock et al. (2015) * (7) | 495.50 | 559.65 | NHS reference costs 2017-18 (18) | 166.7928 | | Kanters et al. (2017) * (20) | | 214.58 | | | Obiluke Consulting (2019)*  (13) |
| Other MH outpatient | per visit | 46.17 – 51.48 | L’Assurance Maladie-AMELI (2019) (19), Average market price (30) | 82.85 | Bock et al. (2015) * (7) | 73 – 167 | 82.45 – 188.62 | NHS reference costs 2017-18 (18) | n.r. | | n.r. | | 65.78 | | | Salvador-Carulla et al. (2011)* (21) |
| MH Inpatient Care | |  |  |  |  |  |  |  |  | |  | |  | | |  |
| Psychiatric hospital inpatient – number of days in hospital in acute psychiatric ward | per bed day | 503.19 | Scansante (2019) (31) | 360.47 | Bock et al. (2015) * (7) | 512.60 | 578.97 | Curtis & Burns (2018) (8) | 298.06 | | Kanters et al. (2017) * (20) | | 184.51 | | | Departament de salut (2018) (10) |
| Psychiatric hospital inpatient – number of days in hospital in PICU | per bed day | 585.50 | Scansante (2019) (31) | 360.47 | Bock et al. (2015) * (7) | 699.04 | 789.54 | Curtis (2012)* (32) | 298.06 | | Kanters et al. (2017) * (20) | | 289.95 | | | Salvador-Carulla et al. (2011)* (21) |
| Psychiatric hospital inpatient – number of days in hospital in PICU | per day case | n.r. | n.r. | n.r. | n.r. | 495.50 | 559.65 | NHS reference costs 2017-18 (18) | n.r. | | n.r. | | n.r. | | | n.r. |
| Psychiatric hospital inpatient – number of days in psychiatric long-stay ward | per inpatient day | 403.81 | Scansante (2019) (31) | 75.13 | Bock et al. (2015) * (7) | 241.67 | 272.96 | Curtis (2010)* (33) | 298.06 | | Kanters et al. (2017) * (20) | | 131.40 | | | Obiluke Consulting (2019)*  (13) |
| Psychiatric hospital inpatient – number of days in psychiatric rehabilitation ward | per bed day | 414.90 | Scansante (2019) (31) | 125.10 | Grupp et al. (2017) (11, 28) | 495.00 | 559.09 | Scottish National Tariff April 2017-March 2018 (34) | 453.99 | | Kanters et al. (2017) * (20) | | 121.28 | | | Obiluke Consulting (2019)*  (13) |
| Non-Mental Health (NMH) Care | |  |  |  |  |  |  |  |  | |  | |  | | |  |
| Primary Care |  |  |  |  |  |  |  |  |  | |  | |  | | |  |
| General Practitioner (GP) | per contact | 25.00 | L’Assurance Maladie-AMELI (2019) (19) | 21.29 | Bock et al. (2015) * (7) | 31.30 | 35.35 | Curtis & Burns (2018) (8) | 32.57 | | Kanters et al. (2017) * (20) | | 43.49 | | | Departament de salut (2013)* (35) |
| GP Practice Nurse | per contact | 16.13 | L’Assurance Maladie-AMELI (2019) (19) | 16.78 | Kanters et al. (2017) * (20) | 9.30 | 10.50 | Curtis & Burns (2018) (8), Curtis (2015)* (36) | 16.78 | | Kanters et al. (2017) * (20) | | 29.09 | | | Departament de salut (2013)* (35) |
| NMH Community Care | |  |  |  |  |  |  |  |  | |  | |  | | |  |
| Community District Nurse | per contact | 21.01 | Rapp et al. (2018)* (6) | 13.12 | Bock et al. (2015) * (7) | 38.00 | 42.92 | NHS reference costs 2017-18 (18) | n.r. | | n.r. | | 25.46 | | | Obiluke Consulting (2019)*  (13) |
| Occupational Therapist | per contact | 16.13 | L’Assurance Maladie-AMELI (2019) (19) | 41.95 | Grupp et al. (2017) (11) | 31.33 | 35.39 | Curtis & Burns (2018) (8), Curtis (2010)* (33) | 32.57 | | Kanters et al. (2017) * (20) | | 17.45 | | | Obiluke Consulting (2019)*  (13) |
| Emergency Services/ Ambulance | per contact | 141.48 | [L’Assurance Maladie-AMELI (2019b)](https://www.ameli.fr/assure/remboursements/rembourse/consultations/modifications-tarifs-consultations-mai-2017) (37) | 523.23 | Feuerwehr Frankfurt (2019) (38) | 184.00 | 207.82 | Curtis & Burns (2018) (8) | 604.99 | | Hakkaart-van Roijen et al. (2016)* (12) | | 268.77 | | | Consejería de Sanidad (2017)* (25) |
| Alternative Care | per contact | 52.50 | Market price acupuncture (39) | 50.00 | Market price acupuncture (40, 41) | 74.00 | 83.58 | NHS reference costs 2017-18 (18) | 82.50 | | Market price acupuncture (42) | | 43.87 | | | Market price (43) |
| Other Health Care | per contact | 16.13 – 35 | L’Assurance Maladie-AMELI (2019) (19) | 17.42 – 59.28 | Bock et al. (2015) * (7) | 52.50 – 135 | 59.30 – 152.48 | NHS reference costs 2017-18 (18), Curtis & Burns (2018) (8) | 32.57 – 89.81 | | Kanters et al. (2017) * (20) | | 15.27 – 135.39 | | | Departament de salut (2013)* (35) |
| NMH Outpatient Care | |  |  |  |  |  |  |  |  | |  | |  | | |  |
| Accident and Emergency | per visit | 139.98 | Gozlan et al. (2018)* (44) | 139.98 | Gozlan et al. (2018)* (44) | 136.00 | 153.61 | NHS reference costs 2017-18 (18) | 255.62 | | Kanters et al. (2017) * (20) | | 105.49 | | | Departament de salut (2018) (10) |
| NMH Outpatient | per visit | 17.15 – 69.33 | Rapp et al. (2018)* (6) | 8.42 – 39.23 | Grupp et al. (2017) (11) | 55 – 308 | 62.12 – 347.88 | NHS reference costs 2017-18 (18), Curtis & Burns (2018) (8) | 89.81 | | Kanters et al. (2017) * (20) | | 43.49 – 135.39 | | | Consejería de Sanidad (2017)* (25), Departament de salut (2013)* (35) |
| NMH Inpatient Care | |  |  |  |  |  |  |  |  | |  | |  | | |  |
| Hospital inpatient – number of days in general medical ward | per bed day | 778.49 | Scansante (2019) (31) | 629.27 | Bock et al. (2015) * (7) | 475.00 | 536.50 | Scottish National Tariff April 2017-March 2018 (34) | 469.78 | | Kanters et al. (2017) * (20) | | 289.95 | | | Bendeck et al. (2013)* (45) |
| Hospital inpatient – number of days in surgical ward | per bed day | 678.73 | Rapp et al. (2018)* (6) | 456.48 | Krauth et al. (2005)*(46) | 865.83 | 977.94 | Scottish National Tariff April 2017-March 2018 (34) | 399.71 | | Kanters et al. (2017) * (20) | | | | | |
| Hospital inpatient – number of days in other medical ward | per bed day | 288.30 – 678.76 | Rapp et al. (2018)* (6) | 147.30 – 755.93 | Bock et al. (2015) * (7) | 272.40 – 276 | | | | Kanters et al. (2017) * (20) | | | | 199.04 – 207.44 | | Departament de salut (2018) (10) |
| Social Care |  |  |  |  |  |  |  |  |  | |  | |  | | |  |
| Social Worker | per hour | n.r. | n.r. | 32.53 | Grupp et al. (2017) (11) | 61.00 | 68.90 | Curtis & Burns (2018) (8) | 64.15 | | Kanters et al. (2017) * (20) | | 52.92 | | | Salvador-Carulla et al. (2011)* (21) |
| Home Helper | per contact of 30 minutes | 9.02 | Rapp et al. (2018)* (6) | 14.96 | Grupp et al. (2017) (11) | 4.97 | 5.61 | Assuming national average salary, ONS (2018) (47) | 21.22 | | Kanters et al. (2017) * (20) | | | | | |
| Home Care Worker | per contact of 30 minutes | n.r. | n.r. | 25.95 | Statistisches Bundesamt (Destatis) (2018)* (48) | 13.88 | 15.67 | Curtis & Burns (2018) (8) | n.r. | | n.r. | |  | | |  |
| Volunteer Helper | per hour | 19.48 | Assuming national average salary, INSEE (2018)* (49) | 24.66 | Statistisches Bundesamt (Destatis) (2018)* (48) | 17.50 | 19.77 | Curtis & Burns (2018) (8) | 13.82 | | Hakkaart-van Roijen et al. (2016)* (12) | | 15.99 | | | Assuming national average salary, INE (2019) (50) |
| Other paid help | per hour | 9.88 | Assuming national average salary, INSEE (2019b) (51) | 14.96 | Grupp et al. (2017) (11) | 9.93 | 11.22 | Assuming national average salary, ONS (2018) (47) | 21.22 | | Kanters et al. (2017) * (20) | | 5.78 | | | Assuming national average salary, BOE (2017) (52) |
| Indirect Costs |  |  |  |  |  |  |  |  |  | |  | |  | | |  |
| Informal Care received from family members/friends | per hour based on proxy good/replacement using hourly wage of a home care worker as formal caregiver | 15.38 | Assuming national average salary, INSEE (2018)* (49) | 25.95 | Assuming national average salary, Statistisches Bundesamt (Destatis) (2018)* (48) | 27.75 | 31.34 | Curtis & Burns (2018) (8) | 13.82 | | Hakkaart-van Roijen et al. (2016)* (12) | | 48.92 | | | Departament de salut (2013)* (35) |
| Informal Care received from family members/friends | per hour based on opportunity cost using the average salary of fulltime employment (income of caregiver) | 19.48 | Assuming national average salary, INSEE (2018)* (49) | 24.66 | Assuming national average salary, Statistisches Bundesamt (Destatis) (2018)* (48) | 17.50 | 19.77 | Assuming national average salary, ONS (2018) (53) | 34.30 | | Hakkaart-van Roijen et al. (2016)* (12) | | 15.99 | | | Assuming national average salary, INE (2019) (50) |
| Lost productivity – fulltime employment | per hour | 19.48 | Assuming national average salary, INSEE (2018)* (49) | 24.66 | Assuming national average salary, Statistisches Bundesamt (Destatis) (2018)* (48) | 17.50 | 19.77 | Assuming national average salary for full-time employment, ONS (2018) (53) | 34.30 | | Hakkaart-van Roijen et al. (2016)* (12) | | 15.99 | | | Assuming national average salary, INE (2019) (50) |
| Lost productivity – parttime employment | per hour | 16.88 | Assuming national average salary, INSEE (2018)* (49) | 19.21 | Assuming national average salary, Statistisches Bundesamt (Destatis) (2018)* (48) | 9.26 | 10.46 | Assuming national average salary for full-time employment, ONS (2018) (53) | 19.73 | | CBS (2019) (54) | | 11.63 | | | Assuming national average salary, INE (2019) (50) |

Note: mg = milligram. mL = millilitre. MH = Mental Health. NMH = Non-Mental Health. n.r. = zero resource use reported. *Adjusted for inflation based on the New Health Services (HS) Index using CPI (Health) for UK and Harmonised Index of Consumer Prices (HICP) for Health from Eurostat for France, Germany, the Netherlands, and Spain (55).

## Supplementary Table S3: Trial-level unit costs (€, year 2018)

|  |  | **HYBRID** |  |  |
| --- | --- | --- | --- | --- |
|  | **Unit** | **Cost in €** | **Source** |  |
| **Medication** |  |  |  |  |
| Oral medication | per mg / mL | various | BNF 76 (September 2018 – March 2019) (3) |  |
| **Mental Health (MH) Care** |  |  |  |  |
| **MH Community Care** |  |  |  |  |
| Community mental health centre | per attendance | 50.83 | Curtis & Burns (2018)* (8) | |
| Day care centre (groups/programs, non-health care staff) | per attendance | 41.79 | Curtis & Burns (2018) (8) | |
| Group therapy | per attendance | 18.07 | Curtis & Burns (2018) (8) | |
| Sheltered workshop | per attendance | 127.61 | Mavranezouli (2014)* (16) |  |
| Specialist education | per attendance | 115.44 | Curtis (2014)* (17) |  |
| Self Help/Support Group | per attendance | 108.43 | NHS reference costs 2017-18 (18) | |
| Community Psychiatrist | per contact | 51.96 | NHS reference costs 2017-18 (18) | |
| Community Psychologist | per contact | 164.90 | NHS reference costs 2017-18 (18) | |
| CPN (psychiatric nurse/case manager) | per contact | 14.97 | Curtis & Burns (2018) (8) | |
| Other MH community-based day services | per attendance | 33.56 – 219.90 | Bock et al. (2015) * (7), Kanters et al. (2017) * (20), L’Assurance Maladie-AMELI (2019) (19), NHS reference costs 2017-18 (18), Curtis & Burns (2017)* (24), Curtis & Burns (2018) (8), Averages of market prices for various service (e.g relaxing massage, reiki session etc.) (22, 23, 26, 27) | |
| **MH Outpatient Care** |  |  |  |  |
| Psychiatric outpatient | per visit | 116.34 | NHS reference costs 2017-18 (18) | |
| Day hospital (group therapies, etc.) | per visit | 559.65 | NHS reference costs 2017-18 (18) | |
| Other MH outpatient | per visit | 46.17 – 188.62 | NHS reference costs 2017-18 (18), Average of market prices for sophrology session (30) | |
| **MH Inpatient Care** |  |  |  |  |
| Psychiatric hospital inpatient – number of days in hospital in acute psychiatric ward | per bed day | 578.97 | Curtis & Burns (2018) (8) | |
| Psychiatric hospital inpatient – number of days in hospital in PICU | per bed day | 789.54 | Curtis (2012)* (32) |  |
| Psychiatric hospital inpatient – number of days in hospital in PICU | per day case | 559.65 | NHS reference costs 2017-18 (18) | |
| Psychiatric hospital inpatient – number of days in psychiatric long-stay ward | per inpatient day | 272.96 | Curtis (2010)* (33) |  |
| Psychiatric hospital inpatient – number of days in psychiatric rehabilitation ward | per bed day | 559.09 | Scottish National Tariff April 2017-March 2018 (34) | |
| **Non-Mental Health (NMH) Care** |  |  |  |  |
| **Primary Care** |  |  |  |  |
| General Practitioner (GP) | per contact | 35.35 | Curtis & Burns (2018) (8) | |
| GP Practice Nurse | per contact | 10.50 | Curtis & Burns (2018) (8), Curtis (2015)* (36) | |
| **NMH Community Care** |  |  |  |  |
| Community District Nurse | per contact | 42.92 | NHS reference costs 2017-18 (18) | |
| Occupational Therapist | per contact | 35.39 | Curtis & Burns (2018) (8), Curtis (2010)* (33) | |
| Emergency Services/ Ambulance | per contact | 207.82 | Curtis & Burns (2018) (8) | |
| Alternative Care | per contact | 83.58 | NHS reference costs 2017-18 (18) | |
| Other Health Care | per contact | 20.04 – 152.48 | NHS reference costs 2017-18 (18), Curtis & Burns (2018) (8), Bock et al. (2015)* (7), Consejería de Sanidad (2017)* (25), Departament de salut (2013)* (35), Kanters et al. (2017)* (20), Krauth et al. (2005)*(46), L'Assurance Maladie-AMELI (2019) 2019) (19) |  |
| **NMH Outpatient Care** |  |  |  |  |
| Accident and Emergency | per visit | 153.61 | NHS reference costs 2017-18 (18) | |
| NMH Outpatient | per visit | 8.42 – 347.88 | NHS reference costs 2017-18 (18), Curtis & Burns (2018) (8), Consejería de Sanidad (2017)* (25), Grupp et al. (2017)* (11, 28) | |
| **NMH Inpatient Care** |  |  |  |  |
| Hospital inpatient – number of days in general medical ward | per bed day | 536.50 | Scottish National Tariff April 2017-March 2018 (34) | |
| Hospital inpatient – number of days in surgical ward | per bed day | 977.94 | Scottish National Tariff April 2017-March 2018 (34) | |
| Hospital inpatient – number of days in other medical (e.g. ) ward | per bed day | 153.18 – 1,479.12 | NHS reference costs 2017-18 (18), Curtis & Burns (2018) (8), Scottish National Tariff April 2017-March 2018 (34), Bock et al. (2015)* (7), Hospital Sainte-Anne (2018) (29), Scansante (2019) (31) | |
| **Social Care** |  |  |  |  |
| Social Worker | per contact of 1 hour | 68.90 | Curtis & Burns (2018) (8) | |
| Home Helper | per contact of 30 minutes | 5.61 | ONS (2018) (47) | |
| Home Care Worker | per contact of 30 minutes | 15.67 | Curtis & Burns (2018) (8) | |
| Volunteer Helper | per hour | 19.77 | Curtis & Burns (2018) (8) | |
| Other paid help | per hour | 11.22 | Assuming national average salary, ONS (2018) (47) | |
| **Indirect Costs** |  |  |  |  |
| Informal Care received from family members/friends | per hour | 31.34 | Curtis & Burns (2018) (8) based on proxy good/replacement using hourly wage of a home care worker as formal caregiver | |
| Informal Care received from family members/friends | per hour | 19.77 | Assuming national average salary of fulltime employment (income of caregiver), ONS (2018) (53) | |
| Lost productivity | per hour | 19.77 | Assuming national average salary for full-time employment, ONS (2018) (53) | |
| Lost productivity | per hour | 10.46 | Assuming national average salary for part-time employment, ONS (2018) (53) | |

Note: mg = milligram. mL = millilitre. MH = Mental Health. NMH = Non-Mental Health. * Adjusted for inflation based on the New Health Services (HS) Index using CPI (Health) for UK and Harmonised Index of Consumer Prices (HICP) for Health from Eurostat for France, Germany, the Netherlands, and Spain (55).

## Supplementary Table S4: Health economic outcomes over time

|  | **week 0** | | | **week 8** | | | **week 24** | | | **week 24 versus week 0** | | | |
| --- | --- | --- | --- | --- | --- | --- | --- | --- | --- | --- | --- | --- | --- |
|  | Mean | SD | p-value^1^ | Mean | SD | p-value^1^ | Mean | SD | p-value^1^ | Mean diff | 95% LCL | 95% UCL | p-value^6^ |
| **EQ-5D-5L index (DE 5L tariff)**  **(n=913)** |  |  |  |  |  |  |  |  |  |  |  |  |  |
| PReDiCT (n=460) | 0.707 | 0.226 |  | 0.849 | 0.176 |  | 0.846 | 0.189 |  | 0.139 | 0.120 | 0.159 | **<0.001** |
| TAU (n=453) | 0.707 | 0.199 |  | 0.834 | 0.173 |  | 0.848 | 0.167 |  | 0.140 | 0.123 | 0.158 | **<0.001** |
| Mean difference^2^ | 0.000 |  | 0.996 | 0.014 |  | 0.210 | -0.001 |  | 0.925 | -0.001 | -0.027 | 0.025 | 0.9370 |
| **EQ-5D-5L index (UK crosswalk 3L tariff) ^3^ (n=913)** |  |  |  |  |  |  |  |  |  |  |  |  |  |
| PReDiCT (n=460) | 0.594 | 0.230 |  | 0.752 | 0.190 |  | 0.761 | 0.197 |  | 0.167 | 0.147 | 0.187 | **<0.001** |
| TAU (n=453) | 0.594 | 0.205 |  | 0.735 | 0.188 |  | 0.755 | 0.180 |  | 0.161 | 0.142 | 0.179 | **<0.001** |
| Mean difference^2^ | -0.001 |  | 0.960 | 0.017 |  | 0.171 | 0.006 |  | 0.648 | 0.006 | -0.021 | 0.033 | 0.6420- |
| **EQ VAS ^4^**  **(n=913)** |  |  |  |  |  |  |  |  |  |  |  |  |  |
| PReDiCT (n=460) | 51.389 | 20.263 |  | 66.273 | 18.236 |  | 66.210 | 18.118 |  | 14.821 | 12.824 | 16.818 | **<0.001** |
| TAU (n=453) | 51.523 | 19.942 |  | 64.127 | 18.733 |  | 64.612 | 17.741 |  | 13.089 | 11.032 | 15.146 | **<0.001** |
| Mean difference^2^ | -0.134 |  | 0.920 | 2.146 |  | 0.080 | 1.599 |  | 0.178 | 1.733 | -1.134 | 4.600 | 0.2360 |
| **OXCAP-MH ^5^**  **(n=619)** |  |  |  |  |  |  |  |  |  |  |  |  |  |
| PReDiCT (n=311) | 60.367 | 14.015 |  | 68.568 | 14.062 |  | 71.423 | 13.068 |  | 11.056 | 9.724 | 12.389 | **<0.001** |
| TAU (n=308) | 60.162 | 12.223 |  | 67.763 | 13.089 |  | 69.092 | 13.176 |  | 8.929 | 7.713 | 10.146 | **<0.001** |
| Mean difference^2^ | 0.204 |  | 0.847 | 0.805 |  | 0.461 | 2.331 |  | 0.027 | 2.127 | 0.323 | 3.931 | **0.0210** |

Note: n = sample size. Imputation based on baseline EQ-5D/OxCAP-MH final score, age, gender, country. n = sample size. SD = standard deviation. PReDicT = PReDicT intervention group, TAU = Treatment-As-Usual group. VAS = Visual Analogue Scale.
^1^ paired t-test, *p<0.05. ^2^ diff = mean(PReDiCT) - mean(TaU). ^3^ Cross-walk calculator generated EQ5D Index values from https://euroqol.org/eq-5d-instruments/eq-5d-5l-about/valuation-standard-value-sets/crosswalk-index-value-calculator/ . ^4^ Imputation based on baseline (week 0) EQ-5D index with DE 5L tariffs. ^5^ Data were only collected in England (UK) and Germany (DE).
^6^ Multilevel mixed-effects linear regression according to group and week

## Supplementary Table S5: Health economic outcomes over 24 weeks by country

| week 24 versus week 0 | | | | | | | | | | | | | | | | | | |
| --- | --- | --- | --- | --- | --- | --- | --- | --- | --- | --- | --- | --- | --- | --- | --- | --- | --- | --- |
|  |  | **Trial level** | | **France** | | | **Germany** | | | **Netherlands** | | | **Spain** | | | **United Kingdom** | | |
|  | n | Mean diff | p-value^1^ | n | Mean diff | p-value^1^ | n | Mean diff | p-value^1^ | n | Mean diff | p-value^1^ | n | Mean diff | p-value^1^ | n | Mean diff | p-value^1^ |
| EQ-5D-5L index | 913 |  |  | 76 |  |  | 130 |  |  | 54 |  |  | 164 |  |  | 489 |  |  |
| *EQ-5D tariff* |  | *5L DE* | | *Crosswalk 3L^4^ FR* | | | *5L DE* | | | *5L NL* | | | *5L ES* | | | *UK crosswalk 3L tariff* | | |
| PReDiCT | 460 | 0.139 | **<0.001** | 39 | 0.1750 | **<0.001** | 63 | 0.1717 | **<0.001** | 28 | 0.1687 | **0.0020** | 82 | 0.1444 | **<0.001** | 248 | 0.1613 | **<0.001** |
| TAU | 453 | 0.140 | **<0.001** | 37 | 0.2438 | **<0.001** | 67 | 0.1394 | **<0.001** | 26 | 0.1888 | **<0.001** | 82 | 0.1572 | **<0.001** | 241 | 0.1525 | **<0.001** |
| Mean diff ^2^ |  | -0.001 | 0.9370 |  | -0.0688 | 0.1240 |  | 0.0323 | 0.3820 |  | -0.0201 | 0.7690 |  | -0.0128 | 0.6220 |  | 0.0088 | 0.6370 |
| EQ VAS ^5^ | 913 |  |  | 76 |  |  |  |  |  | 54 |  |  | 164 |  |  | 489 |  |  |
| PReDiCT | 460 | 3.615 | **<0.001** | 39 | 20.4371 | **<0.001** | 63 | 12.0336 | **<0.001** | 28 | 12.6148 | **0.0010** | 82 | 0.1444 | **<0.001** | 248 | 17.3909 | **<0.001** |
| TAU | 453 | 3.868 | **<0.001** | 37 | 22.2111 | **<0.001** | 67 | 9.6084 | **<0.001** | 26 | 13.6951 | **<0.001** | 82 | 0.1572 | **<0.001** | 241 | 14.6771 | **<0.001** |
| Mean diff ^2^ |  | -0.253 | 0.8230 |  | -1.7740 | 0.7260 |  | 2.4252 | 0.5230 |  | -1.0803 | 0.8280 |  | -0.0128 | 0.6220 |  | 2.7138 | 0.1810 |
| OXCAP-MH ^3^ | 619 |  |  |  |  |  | 130 |  |  |  |  |  |  |  |  | 489 |  |  |
| PReDiCT | 311 | 11.056 | **<0.001** | .. | .. | .. | 63 | 7.5907 | **<0.001** | .. | .. | .. | .. | .. | .. | 248 | 11.9367 | **<0.001** |
| TAU | 308 | 8.929 | **<0.001** | .. | .. | .. | 67 | 7.4185 | **<0.001** | .. | .. | .. | .. | .. | .. | 241 | 9.3495 | **<0.001** |
| Mean diff ^2^ |  | 2.127 | **0.0210** | .. | .. | .. |  | 0.1721 | 0.9320 | .. | .. | .. | .. | .. | .. |  | 2.5872 | **0.0120** |

Note: n = sample size. SD = standard deviation. PReDicT = PReDicT intervention group, TAU = Treatment-As-Usual group. VAS = Visual Analogue Scale. FR = France, DE = Germany, NL = The Netherlands, ES = Spain, UK = United Kingdom. .. = not available.
^1^ Multilevel mixed-effects linear regression according to group and week. ^2^ Mean diff = difference: mean(PReDiCT) – mean(TAU). ^3^ Data were only collected in UK and DE. ^4^ The EQ-5D-5L Crosswalk Index Value Calculator 2019 (56) was used based on van Hout et al. (2012) (57): for FR (58) and for UK (59).

##

## Supplementary Table S6: Observed health and social care resource use

|  | **DURING Trial (week 0 to week 24)** | | | | | | **BEFORE Trial (-week 4 to week 0)** | | | | | | |
| --- | --- | --- | --- | --- | --- | --- | --- | --- | --- | --- | --- | --- | --- |
|  | **PReDicT** | | | **TAU** | | | **PReDicT** | | | **TAU** | | | |
|  | **n** | **Mean** | **SD** | **n** | **Mean** | **SD** | **n** | **Mean** | **SD** | **n** | **Mean** | **SD** |  |
| **Intervention** |  |  |  |  |  |  |  |  |  |  |  |  |  |
| Time spent on PReDicT test delivery (minutes) | 274 | 29.70 | 114.66 | . | . | . | . | . | . | . | . | . |  |
| Time spent on PReDicT test completion (minutes) | 274 | 139.26 | 435.20 | . | . | . | . | . | . | . | . | . |  |
| **Mental Health Care** |  |  |  |  |  |  |  |  |  |  |  |  |  |
| **MH Community Care** |  |  |  |  |  |  |  |  |  |  |  |  |  |
| Community mental health centre - attendance | 274 | 0.04 | 0.35 | 260 | 0.05 | 0.44 | 274 | 0.06 | 0.34 | 260 | 0.04 | 0.25 |  |
| Day care centre - attendance | 274 | 0.01 | 0.12 | 260 | 0.03 | 0.39 | 274 | 0.00 | 0.06 | 260 | 0.02 | 0.26 |  |
| Group therapy - attendance | 274 | 0.00 | 0.00 | 260 | 0.29 | 2.16 | 274 | 0.07 | 0.81 | 260 | 0.02 | 0.26 |  |
| Sheltered workshop - attendance | 274 | 0.13 | 2.00 | 260 | 0.01 | 0.12 | 274 | 0.00 | 0.00 | 260 | 0.03 | 0.50 |  |
| Specialist education - attendance | 274 | 0.13 | 2.00 | 260 | 0.00 | 0.00 | 274 | 0.00 | 0.00 | 260 | 0.00 | 0.00 |  |
| Self Help/Support Group - attendance | 274 | 0.12 | 1.50 | 260 | 0.12 | 1.39 | 274 | 0.01 | 0.12 | 260 | 0.03 | 0.40 |  |
| Community Psychiatrist - contact | 274 | 0.28 | 1.73 | 260 | 0.33 | 2.11 | 274 | 0.27 | 0.61 | 260 | 0.25 | 0.58 |  |
| Community Psychologist - contact | 274 | 0.64 | 2.69 | 260 | 0.32 | 1.28 | 274 | 0.50 | 1.33 | 260 | 0.43 | 1.21 |  |
| CPN (psychiatric nurse/case manager) - contact | 274 | 0.04 | 0.61 | 260 | 0.14 | 1.40 | 274 | 0.01 | 0.12 | 260 | 0.02 | 0.17 |  |
| Other MH community-based day services - attendance | 274 | 0.06 | 0.67 | 260 | 0.01 | 0.09 | 274 | 0.03 | 0.28 | 260 | 0.00 | 0.06 |  |
| **MH Outpatient Care** |  |  |  |  |  |  |  |  |  |  |  |  |  |
| Psychiatric outpatient - visit | 274 | 0.19 | 1.49 | 260 | 0.64 | 5.27 | 274 | 0.12 | 0.49 | 260 | 0.16 | 0.51 |  |
| Day hospital - visit | 274 | 0.03 | 0.43 | 260 | 0.05 | 0.49 | 274 | 0.00 | 0.00 | 260 | 0.02 | 0.26 |  |
| Other MH outpatient - visit | 274 | 0.19 | 2.61 | 260 | 0.00 | 0.00 | 274 | 0.01 | 0.24 | 260 | 0.00 | 0.00 |  |
| **MH Inpatient Care** |  |  |  |  |  |  |  |  |  |  |  |  |  |
| Psychiatric hospital inpatient - number of days in hospital in acute psychiatric ward | 274 | 0.25 | 2.78 | 260 | 0.19 | 2.64 | 274 | 0.02 | 0.21 | 260 | 0.00 | 0.00 |  |
| Psychiatric hospital inpatient - number of days in hospital in PICU | 274 | 0.00 | 0.00 | 260 | 0.08 | 1.06 | 274 | 0.00 | 0.00 | 260 | 0.04 | 0.62 |  |
| Psychiatric hospital inpatient - number of days in psychiatric long-stay ward | 274 | 0.46 | 4.02 | 260 | 0.45 | 4.35 | 274 | 0.00 | 0.00 | 260 | 0.01 | 0.19 |  |
| Psychiatric hospital inpatient - number of days in psychiatric rehabilitation ward | 274 | 0.03 | 0.48 | 260 | 0.00 | 0.00 | 274 | 0.00 | 0.00 | 260 | 0.00 | 0.00 |  |
| **Non-Mental Health Care** |  |  |  |  |  |  |  |  |  |  |  |  |  |
| **Primary Care** |  |  |  |  |  |  |  |  |  |  |  |  |  |
| General Practitioner (GP) - contact | 274 | 0.45 | 1.59 | 260 | 0.35 | 1.11 | 274 | 0.75 | 0.95 | 260 | 0.82 | 1.04 |  |
| GP Practice Nurse - contact | 274 | 0.28 | 1.97 | 260 | 0.11 | 1.01 | 274 | 0.12 | 0.48 | 260 | 0.10 | 0.39 |  |
| **NMH Community Care** |  |  |  |  |  |  |  |  |  |  |  |  |  |
| Community District Nurse - contact | 274 | 0.00 | 0.06 | 260 | 0.01 | 0.19 | 274 | 0.02 | 0.36 | 260 | 0.01 | 0.12 |  |
| Occupational Therapist - contact | 274 | 0.01 | 0.10 | 260 | 0.05 | 0.50 | 274 | 0.01 | 0.10 | 260 | 0.02 | 0.19 |  |
| Emergency Services/ Ambulance - contact | 274 | 0.02 | 0.12 | 260 | 0.03 | 0.21 | 274 | 0.03 | 0.20 | 260 | 0.02 | 0.14 |  |
| Alternative Care - contact | 274 | 0.02 | 0.17 | 260 | 0.07 | 0.51 | 274 | 0.11 | 0.84 | 260 | 0.04 | 0.30 |  |
| Other Health Care - contact | 274 | 0.22 | 2.79 | 260 | 0.15 | 1.33 | 274 | 0.24 | 1.64 | 260 | 0.08 | 0.59 |  |
| **NMH Outpatient Care** |  |  |  |  |  |  |  |  |  |  |  |  |  |
| Accident and Emergency - visit | 274 | 0.05 | 0.33 | 260 | 0.12 | 0.58 | 274 | 0.10 | 0.34 | 260 | 0.07 | 0.32 |  |
| NMH Outpatient - visit | 274 | 0.28 | 1.66 | 260 | 0.36 | 1.84 | 274 | 0.18 | 0.58 | 260 | 0.20 | 1.01 |  |
| **NMH Inpatient Care** |  |  |  |  |  |  |  |  |  |  |  |  |  |
| Hospital inpatient - number of days in general medical ward | 274 | 0.05 | 0.57 | 260 | 0.05 | 0.69 | 274 | 0.01 | 0.15 | 260 | 0.02 | 0.20 |  |
| Hospital inpatient - number of days in surgical ward | 274 | 0.07 | 0.46 | 260 | 0.05 | 0.51 | 274 | 0.05 | 0.41 | 260 | 0.01 | 0.09 |  |
| Hospital inpatient - number of days in other medical ward | 274 | 0.01 | 0.12 | 260 | 0.40 | 3.65 | 274 | 0.01 | 0.09 | 260 | 0.02 | 0.15 |  |
| **Social Care** |  |  |  |  |  |  |  |  |  |  |  |  |  |
| Social Worker - contact | 274 | 0.00 | 0.00 | 260 | 0.03 | 0.38 | 274 | 0.00 | 0.06 | 260 | 0.10 | 1.26 |  |
| Home Helper - contact | 274 | 0.00 | 0.00 | 260 | 0.00 | 0.00 | 274 | 0.00 | 0.00 | 260 | 0.01 | 0.14 |  |
| Home Care Worker - contact | 274 | 0.08 | 1.33 | 260 | 0.01 | 0.08 | 274 | 0.05 | 0.73 | 260 | 0.00 | 0.01 |  |
| Volunteer Helper - hours | 274 | 0.37 | 5.15 | 260 | 1.14 | 8.42 | 274 | 0.07 | 0.67 | 260 | 0.04 | 0.35 |  |
| Other paid help - hours | 274 | 0.49 | 3.46 | 260 | 0.22 | 2.20 | 274 | 0.10 | 0.74 | 260 | 0.05 | 0.57 |  |
| Informal Care received from family members/friends - hours | 274 | 14.52 | 57.07 | 260 | 15.28 | 78.83 | 274 | 3.64 | 12.77 | 260 | 2.44 | 8.18 |  |

Note: Note: n = sample size. SD = standard deviation. PReDicT = PReDicT intervention group, TAU = Treatment-As-Usual group.

##

## Supplementary Table S7a: Average number of antidepressants taken per participant

|  | **PReDiCT** | | | | | **TAU** | | | | |
| --- | --- | --- | --- | --- | --- | --- | --- | --- | --- | --- |
|  | **n** | **Mean** | **SD** | **Min** | **Max** | **n** | **Mean** | **SD** | **Min** | **Max** |
| **Trial level** | 460 | 1.17 | 0.52 | 0 | 5 | 453 | 1.20 | 0.54 | 0 | 4 |
| **France** | 39 | 1.03 | 0.43 | 0 | 2 | 37 | 1.11 | 0.61 | 0 | 4 |
| **Germany** | 63 | 1.40 | 0.83 | 0 | 5 | 67 | 1.37 | 0.74 | 0 | 4 |
| **Netherlands** | 28 | 1.36 | 0.73 | 0 | 3 | 26 | 1.15 | 0.37 | 1 | 2 |
| **Spain** | 82 | 1.09 | 0.32 | 0 | 2 | 82 | 1.15 | 0.45 | 0 | 3 |
| **United Kingdom** | 248 | 1.13 | 0.42 | 0 | 3 | 241 | 1.20 | 0.51 | 0 | 4 |

Note: n = sample size. SD = standard deviation. PReDicT = PReDicT intervention group, TAU = Treatment-As-Usual group.

## Supplementary Table S7b: Number of different types of antidepressants taken by participants

| **Types of antidepressants taken** | **Trial Level** | | **France** | | **Germany** | | **Netherlands** | | **Spain** | | **United Kingdom** | |
| --- | --- | --- | --- | --- | --- | --- | --- | --- | --- | --- | --- | --- |
|  | PReDiCT | TAU | PReDiCT | TAU | PReDiCT | TAU | PReDiCT | TAU | PReDiCT | TAU | PReDiCT | TAU |
| 0 | 11 | 7 | 3 | 2 | 2 | 1 | 1 | 0 | 1 | 1 | 4 | 3 |
| 1 | 377 | 367 | 32 | 31 | 42 | 48 | 19 | 22 | 73 | 70 | 211 | 196 |
| 2 | 60 | 62 | 4 | 3 | 14 | 11 | 5 | 4 | 8 | 9 | 29 | 35 |
| 3 | 10 | 14 | 0 | 0 | 3 | 6 | 3 | 0 | 0 | 2 | 4 | 6 |
| 4 | 1 | 3 | 0 | 1 | 1 | 1 | 0 | 0 | 0 | 0 | 0 | 1 |
| 5 | 1 | 0 | 0 | 0 | 1 | 0 | 0 | 0 | 0 | 0 | 0 | 0 |

Note: Note: n = sample size. PReDicT = PReDicT intervention group, TAU = Treatment-As-Usual group.

## Supplementary Table S8: Mean costs per participant - France (€, year 2018, n=76)

|  | **DURING Trial (week 0 to week 24)** | | | | | | | | | **BEFORE Trial vs. DURING Trial costs** | | | | | | | | |
| --- | --- | --- | --- | --- | --- | --- | --- | --- | --- | --- | --- | --- | --- | --- | --- | --- | --- | --- |
|  | **PReDicT (n=39)** | | **TAU (n=37)** | | **Mean diff^1^** |  | **p-value** | **95% LCL** | **95% UCL** | **PReDicT (n=39)** | | | | **TAU (n=37)** | | | |  |
|  | **Mean** | **SD** | **Mean** | **SD** |  |  |  |  |  | **Mean diff^2^** | **95% LCL** | **95% UCL** | **p-value** | **Mean diff^2^** | **95% LCL** | **95% UCL** | **p-value** |  |
| **A) Intervention (PReDicT test) costs** | **101.26** | **213.48** | **0.00** | **0.00** | **101.26** |  | **0.01** | **31.30** | **171.21** | **101.26** | **31.30** | **171.21** | **0.01** |  |  |  |  |  |
| **B) Total medication costs** | **75.63** | **88.60** | **166.38** | **312.18** | **-90.75** |  | **0.05** | **-181.94** | **0.44** | **56.20** | **41.45** | **70.95** | **0.00** | **108.99** | **46.74** | **171.24** | **0.00** |  |
| Antidepressants (BNF chapter 04.03) | 50.14 | 44.14 | 66.02 | 134.30 | -15.88 |  | 0.45 | -56.98 | 25.22 | 49.41 | 35.14 | 63.69 | 0.00 | 65.96 | 21.20 | 110.73 | 0.01 |  |
| Other MH (BNF chapter 04.01/02/04) | 13.45 | 36.71 | 66.67 | 268.71 | -53.21 |  | 0.16 | -128.19 | 21.76 | 6.78 | 0.59 | 12.97 | 0.03 | 30.66 | -9.19 | 70.50 | 0.13 |  |
| Other Nervous System (BNF chapter 04.05-11) | 12.04 | 75.19 | 33.69 | 125.45 | -21.65 |  | 0.48 | -81.14 | 37.83 | 0.00 | 0.00 | 0.00 | 0.32 | 12.37 | -12.40 | 37.13 | 0.32 |  |
| **C) Mental Health Care** | **1,522.84** | **5,064.59** | **1,409.45** | **4,759.47** | **113.38** |  | **0.92** | **-2,095.83** | **2,322.60** | **-1,499.42** | **-6,168.35** | **3,169.51** | **0.52** | **91.60** | **-1,701.44** | **1,884.65** | **0.92** |  |
| MH Community Care | 117.79 | 308.92 | 152.46 | 463.85 | -34.67 |  | 0.70 | -208.95 | 139.60 | -630.98 | -830.51 | -431.45 | 0.00 | -828.80 | -1,453.41 | -204.20 | 0.01 |  |
| MH Outpatient Care | 86.09 | 409.91 | 84.10 | 352.27 | 1.99 |  | 0.98 | -169.91 | 173.88 | -32.70 | -193.69 | 128.29 | 0.68 | -252.48 | -553.81 | 48.84 | 0.10 |  |
| MH Inpatient Care | 1,318.96 | 4,541.84 | 1,172.89 | 4,327.97 | 146.07 |  | 0.89 | -1,852.51 | 2,144.65 | -835.74 | -5,450.87 | 3,779.40 | 0.72 | 1,172.89 | -270.13 | 2,615.91 | 0.11 |  |
| **A+B+C** | **1,699.73** | **5,048.46** | **1,575.83** | **4,778.30** | **123.89** |  | **0.91** | **-2,085.93** | **2,333.72** | -1,341.97 | -6,014.94 | 3,331.01 | 0.56 | 200.59 | -1,599.40 | 2,000.58 | 0.82 |  |
| **D) Non-Mental Health Care** | **131.62** | **319.38** | **477.08** | **2,410.89** | **-345.47** |  | **0.28** | **-971.12** | **280.19** | **-481.81** | **-800.70** | **-162.93** | **0.00** | **-117.06** | **-836.14** | **602.02** | **0.74** |  |
| Primary Care | 15.93 | 66.58 | 9.38 | 40.12 | 6.55 |  | 0.60 | -18.15 | 31.25 | -87.67 | -147.89 | -27.44 | 0.01 | -96.03 | -147.93 | -44.12 | 0.00 |  |
| NMH Community Care | 12.87 | 31.87 | 18.01 | 57.18 | -5.14 |  | 0.62 | -25.20 | 14.93 | -106.72 | -186.96 | -26.48 | 0.01 | -51.43 | -122.17 | 19.32 | 0.15 |  |
| NMH Outpatient Care | 48.69 | 198.62 | 20.29 | 51.16 | 28.40 |  | 0.33 | -28.31 | 85.11 | -67.81 | -181.10 | 45.47 | 0.23 | -36.46 | -118.94 | 46.01 | 0.38 |  |
| NMH Inpatient Care | 54.12 | 226.63 | 429.40 | 2,366.13 | -375.28 |  | 0.28 | -1,055.06 | 304.50 | -219.61 | -501.35 | 62.12 | 0.12 | 66.85 | -708.71 | 842.41 | 0.86 |  |
| **Perspective I: A+B+C+D  (HEALTH CARE)** | **1,831.34** | **5,092.69** | **2,052.91** | **5,329.77** | **-221.57** |  | **0.85** | **-2,581.13** | **2,137.98** | **-1,823.78** | **-6,502.42** | **2,854.87** | **0.43** | **83.53** | **-1,875.55** | **2,042.61** | **0.93** |  |
| **E) Social Care** | **26.19** | **70.67** | **11.61** | **47.95** | **14.57** |  | **0.36** | **-16.76** | **45.90** | **26.19** | **3.28** | **49.09** | **0.03** | **-4.27** | **-24.77** | **16.23** | **0.68** |  |
| **Perspective II: A+B+C+D+E  (HEALTH AND SOCIAL CARE)** | **1,857.53** | **5,128.82** | **2,064.53** | **5,334.77** | **-207.00** |  | **0.86** | **-2,574.37** | **2,160.37** | **-1,797.59** | **-6,477.11** | **2,881.93** | **0.44** | **79.26** | **-1,882.51** | **2,041.03** | **0.94** |  |
| **F) Indirect Costs** | **4,225.70** | **6,296.03** | **3,557.22** | **6,042.55** | **668.47** |  | **0.64** | **-2,128.15** | **3,465.09** | **-1,754.24** | **-2,763.69** | **-744.78** | **0.00** | **-1,646.34** | **-3,820.91** | **528.23** | **0.13** |  |
| Informal Care | 175.26 | 677.15 | 18.22 | 41.98 | 157.05 |  | 0.08 | -20.06 | 334.16 | 9.60 | -167.29 | 186.49 | 0.91 | -14.21 | -44.68 | 16.26 | 0.35 |  |
| Transport Expenses | 12.49 | 49.94 | 2.49 | 7.26 | 10.00 |  | 0.16 | -4.06 | 24.06 | -11.90 | -33.24 | 9.45 | 0.27 | -11.83 | -27.75 | 4.09 | 0.14 |  |
| Lost Household Productivity | **1,074.22** | **1,370.83** | **1,104.09** | **1,228.27** | **-29.87** |  | **0.92** | **-618.30** | **558.56** | **-1,103.44** | **-1,577.91** | **-628.97** | **0.00** | **-711.34** | **-1,195.86** | **-226.83** | **0.01** |  |
| Lost Productivity Costs | **2,963.73** | **5,837.07** | **2,432.43** | **5,213.17** | **531.30** |  | **0.68** | **-1,969.29** | **3,031.89** | **-648.49** | **-1,649.58** | **352.59** | **0.20** | **-908.96** | **-2,829.61** | **1,011.69** | **0.34** |  |
| Absenteeism Cost | 2,444.60 | 5,273.56 | 2,194.49 | 5,221.22 | 250.11 |  | 0.84 | -2,116.25 | 2,616.48 | -123.89 | -1,003.45 | 755.67 | 0.78 | 184.72 | -1,533.25 | 1,902.69 | 0.83 |  |
| Presenteeism Cost | 519.13 | 1,017.28 | 237.94 | 662.04 | 281.18 |  | 0.20 | -150.15 | 712.52 | -524.60 | -1,172.61 | 123.40 | 0.11 | -1,093.68 | -1,990.84 | -196.52 | 0.02 |  |
| **Perspective III: A+B+C+D+E+F (SOCIETAL)** | **6,083.22** | **7,939.46** | **5,621.75** | **9,911.09** | **461.47** |  | **0.82** | **-3,606.38** | **4,529.32** | **-3,551.83** | **-8,405.08** | **1,301.43** | **0.15** | **-1,567.08** | **-4,583.21** | **1,449.05** | **0.30** |  |

Note: n = sample size. SD = standard deviation. PReDicT = PReDicT intervention group, TAU = Treatment-As-Usual group. .. = not available. LCL = lower confidence level. UCL = upper confidence level.  ^1^ Mean diff = mean(PReDiCT) – mean(TAU). Multiple imputation included treatment group, age, sex, country, and baseline costs as predictors. Means costs by group and the adjusted difference were estimated using a generalized linear model (GLM) with family gamma and link log (cost as the dependent variable and treatment group as the independent variable), using the margins command. ^2^ Mean diff = mean(DURING trial costs) – mean(BEFORE Trial costs); ttest.

## Supplementary Table S9: Mean costs per participant - Germany (€, year 2018, n=130)

|  | **DURING Trial (week 0 to week 24)** | | | | | | | | **BEFORE Trial vs. DURING Trial costs** | | | | | | | |
| --- | --- | --- | --- | --- | --- | --- | --- | --- | --- | --- | --- | --- | --- | --- | --- | --- |
|  | **PReDicT (n=63)** | | **TAU (n=67)** | | **Mean diff^1^** | **p-value** | **95% LCL** | **95% UCL** | **PReDicT (n=63)** | | | | **TAU (n=67)** | | | |
|  | **Mean** | **SD** | **Mean** | **SD** |  |  |  |  | **Mean diff3** | **95% LCL** | **95% UCL** | **p-value** | **Mean diff^2^** | **95% LCL** | **95% UCL** | **p-value** |
| **A) Intervention (PReDicT test) costs** | **95.46** | **139.18** | **0.00** | **0.00** | **95.46** | **0.00** | **61.82** | **129.09** | **95.46** | **61.82** | **129.09** | **0.00** | **.** | **.** | **.** | **.** |
| **B) Total medication costs** | **255.31** | **392.10** | **236.40** | **341.90** | **18.91** | **0.77** | **-107.38** | **145.21** | **244.21** | **146.72** | **341.71** | **0.00** | **234.26** | **150.83** | **317.68** | **0.00** |
| Antidepressants (BNF chapter 04.03) | 156.28 | 118.07 | 187.26 | 273.62 | -30.98 | 0.38 | -100.41 | 38.46 | 156.12 | 126.48 | 185.75 | 0.00 | 186.77 | 120.01 | 253.54 | 0.00 |
| Other MH (BNF chapter 04.01/02/04) | 85.71 | 292.11 | 44.71 | 138.18 | 41.00 | 0.30 | -36.06 | 118.06 | 81.95 | 8.12 | 155.78 | 0.03 | 43.06 | 9.70 | 76.42 | 0.01 |
| Other Nervous System (BNF chapter 04.05-11) | 13.31 | 64.49 | 4.43 | 21.29 | 8.88 | 0.30 | -7.79 | 25.56 | 6.14 | -3.77 | 16.05 | 0.22 | 4.43 | -0.77 | 9.62 | 0.09 |
| **C) Mental Health Care** | **1,360.13** | **3,655.78** | **596.58** | **2,252.31** | **763.56** | **0.21** | **-438.59** | **1,965.70** | **-296.23** | **-1,681.46** | **1,088.99** | **0.67** | **-836.76** | **-1,453.18** | **-220.34** | **0.01** |
| MH Community Care | 132.77 | 341.03 | 114.77 | 285.48 | 18.01 | 0.74 | -90.10 | 126.12 | -346.41 | -521.21 | -171.61 | 0.00 | -678.55 | -880.53 | -476.58 | 0.00 |
| MH Outpatient Care | 178.05 | 738.93 | 67.50 | 318.64 | 110.56 | 0.30 | -98.12 | 319.23 | -37.89 | -240.69 | 164.91 | 0.71 | -174.85 | -273.66 | -76.04 | 0.00 |
| MH Inpatient Care | 1,049.31 | 3,510.65 | 414.32 | 2,146.31 | 634.99 | 0.31 | -582.96 | 1,852.95 | 88.06 | -1,274.68 | 1,450.81 | 0.90 | 16.64 | -552.43 | 585.72 | 0.95 |
| **A+B+C** | **1,710.90** | **3,826.23** | **832.97** | **2,312.18** | **877.92** | **0.15** | **-304.89** | **2,060.74** | **43.44** | **-1,371.31** | **1,458.18** | **0.95** | **-602.50** | **-1,224.27** | **19.27** | **0.06** |
| **D) Non-Mental Health Care** | **136.78** | **369.65** | **189.12** | **663.68** | **-52.34** | **0.56** | **-229.98** | **125.30** | **-736.58** | **-1,405.63** | **-67.52** | **0.03** | **-824.85** | **-1,981.36** | **331.66** | **0.16** |
| Primary Care | 12.60 | 32.36 | 12.16 | 24.19 | 0.44 | 0.93 | -9.31 | 10.19 | -141.04 | -180.79 | -101.28 | 0.00 | -141.43 | -179.49 | -103.38 | 0.00 |
| NMH Community Care | 17.15 | 82.25 | 40.20 | 165.94 | -23.05 | 0.34 | -70.01 | 23.90 | -153.95 | -326.26 | 18.36 | 0.08 | -81.47 | -221.73 | 58.80 | 0.25 |
| NMH Outpatient Care | 10.93 | 24.39 | 25.14 | 66.65 | -14.20 | 0.09 | -30.42 | 2.01 | -146.41 | -238.38 | -54.45 | 0.00 | -65.44 | -179.90 | 49.03 | 0.26 |
| NMH Inpatient Care | 96.09 | 352.08 | 111.62 | 563.89 | -15.52 | 0.85 | -174.01 | 142.96 | -295.18 | -858.63 | 268.28 | 0.30 | -536.52 | -1,565.95 | 492.92 | 0.30 |
| **Perspective I: A+B+C+D  (HEALTH CARE)** | **1,847.68** | **3,853.45** | **1,022.09** | **2,409.02** | **825.59** | **0.16** | **-329.50** | **1,980.68** | **-693.14** | **-2,253.83** | **867.55** | **0.38** | **-1,427.35** | **-2,837.66** | **-17.05** | **0.05** |
| **E) Social Care** | **88.07** | **293.66** | **32.70** | **90.54** | **55.37** | **0.13** | **-15.28** | **126.03** | **-36.41** | **-175.55** | **102.74** | **0.60** | **-14.23** | **-38.68** | **10.21** | **0.25** |
| **Perspective II: A+B+C+D+E  (HEALTH AND SOCIAL CARE)** | **1,935.75** | **3,860.65** | **1,054.79** | **2,406.23** | **880.96** | **0.14** | **-280.55** | **2,042.48** | **-729.55** | **-2,298.59** | **839.50** | **0.36** | **-1,441.59** | **-2,849.07** | **-34.10** | **0.04** |
| **F) Indirect Costs** | **8,096.96** | **10,308.90** | **6,089.54** | **8,639.68** | **2,007.43** | **0.24** | **-1,337.62** | **5,352.47** | **-3,465.31** | **-5,394.10** | **-1,536.53** | **0.00** | **-3,597.72** | **-5,500.92** | **-1,694.52** | **0.00** |
| Informal Care | 246.07 | 438.04 | 216.85 | 593.28 | 29.22 | 0.76 | -156.43 | 214.88 | -715.42 | -1,283.32 | -147.52 | 0.01 | -436.24 | -856.59 | -15.89 | 0.04 |
| Transport Expenses | 1.48 | 3.36 | 1.61 | 4.28 | -0.13 | 0.84 | -1.45 | 1.18 | -12.11 | -17.00 | -7.22 | 0.00 | -16.57 | -20.77 | -12.38 | 0.00 |
| Lost Household Productivity | 1,491.46 | 1,635.19 | 1,608.61 | 1,656.29 | -117.15 | 0.69 | -683.56 | 449.27 | -2,294.88 | -2,889.63 | -1,700.13 | 0.00 | -1,776.58 | -2,389.86 | -1,163.31 | 0.00 |
| Lost Productivity Costs | 6,357.95 | 10,044.17 | 4,262.47 | 8,335.14 | 2,095.48 | 0.22 | -1,244.72 | 5,435.68 | -442.90 | -2,190.90 | 1,305.09 | 0.61 | -1,368.33 | -2,988.22 | 251.56 | 0.10 |
| Absenteeism Cost | 5,877.86 | 9,704.24 | 3,710.04 | 8,307.99 | 2,167.81 | 0.21 | -1,197.37 | 5,532.99 | 219.30 | -1,597.96 | 2,036.55 | 0.81 | -98.71 | -1,710.43 | 1,513.01 | 0.90 |
| Presenteeism Cost | 480.10 | 1,513.10 | 552.43 | 1,783.69 | -72.33 | 0.80 | -639.29 | 494.62 | -662.20 | -1,347.31 | 22.91 | 0.06 | -1,269.62 | -1,968.30 | -570.94 | 0.00 |
| **Perspective III: A+B+C+D+E+F (SOCIETAL)** | **10,032.72** | **12,128.05** | **7,144.33** | **9,145.46** | **2,888.38** | **0.13** | **-864.75** | **6,641.52** | **-4,194.86** | **-6,971.00** | **-1,418.72** | **0.00** | **-5,039.31** | **-7,510.34** | **-2,568.27** | **0.00** |

Note: n = sample size. SD = standard deviation. PReDicT = PReDicT intervention group, TAU = Treatment-As-Usual group. .. = not available. LCL = lower confidence level. UCL = upper confidence level.  ^1^ Mean diff = mean(PReDiCT) – mean(TAU). Multiple imputation included treatment group, age, sex, country, and baseline costs as predictors. Means costs by group and the adjusted difference were estimated using a generalized linear model (GLM) with family gamma and link log (cost as the dependent variable and treatment group as the independent variable), using the margins command. ^2^ Mean diff = mean(DURING trial costs) – mean(BEFORE Trial costs); ttest.

## Supplementary Table S10: Mean costs per participant - Netherlands (€, year 2018, n=54)

|  | **DURING Trial (week 0 to week 24)** | | | | | | | | **BEFORE Trial vs. DURING Trial costs** | | | | | | | | |
| --- | --- | --- | --- | --- | --- | --- | --- | --- | --- | --- | --- | --- | --- | --- | --- | --- | --- |
|  | **PReDicT (n=28)** | | **TAU (n=26)** | | **Mean diff1** | **p-value** | **95% LCL** | **95% UCL** | **PReDicT (n=28)** | | | | **TAU (n=26)** | | | | |
|  | **Mean** | **SD** | **Mean** | **SD** |  |  |  |  | **Mean diff2** | **95% LCL** | **95% UCL** | **p-value** | **Mean diff2** | **95% LCL** | **95% UCL** | **p-value** |  |
| **A) Intervention (PReDicT test) costs** | **185.40** | **373.70** | **0.00** | **0.00** |  | **0.02** | **38.23** | **332.56** | **185.40** | **38.23** | **332.56** | **0.02** | **.** | **.** | **.** | **.** |  |
| **B) Total medication costs** | **111.94** | **312.19** | **35.59** | **45.10** | **76.35** | **0.12** | **-19.42** | **172.11** | **91.62** | **305.38** | **-26.79** | **0.12** | **27.30** | **19.47** | **19.44** | **0.00** |  |
| Antidepressants (BNF chapter 04.03) | 34.97 | 29.49 | 27.12 | 19.50 | 7.85 | 0.24 | -5.22 | 20.92 | 34.97 | 29.49 | 23.53 | 0.00 | 27.12 | 19.50 | 19.24 | 0.00 |  |
| Other MH (BNF chapter 04.01/02/04) | 4.37 | 20.62 | 1.25 | 2.77 | 3.12 | 0.33 | -3.19 | 9.42 | -0.28 | 3.09 | -1.47 | 0.64 | 0.18 | 1.61 | -0.47 | 0.57 |  |
| Other Nervous System (BNF chapter 04.05-11) | 72.61 | 308.45 | 7.23 | 30.12 | 65.38 | 0.26 | -48.44 | 179.19 | 56.93 | 300.78 | -59.70 | 0.33 | 0.00 | 0.00 | 0.00 | 0.24 |  |
| **C) Mental Health Care** | **681.93** | **1,033.09** | **967.74** | **2,579.57** | **-285.80** | **0.56** | **-1,250.98** | **679.37** | **-834.57** | **1,515.17** | **-1,422.09** | **0.01** | **47.59** | **2,759.68** | **-1,067.07** | **0.93** |  |
| MH Community Care | 532.60 | 749.60 | 282.24 | 545.30 | 250.36 | 0.20 | -128.05 | 628.77 | -719.13 | 1,264.18 | -1,209.32 | 0.01 | -503.99 | 939.37 | -883.41 | 0.01 |  |
| MH Outpatient Care | 139.15 | 419.24 | 473.84 | 1,555.84 | -334.69 | 0.27 | -930.30 | 260.93 | 35.52 | 86.24 | 2.09 | 0.04 | 339.92 | 1,437.71 | -240.78 | 0.24 |  |
| MH Inpatient Care | 10.18 | 31.99 | 211.66 | 992.55 | -201.48 | 0.22 | -524.20 | 121.25 | -150.97 | 605.47 | -385.75 | 0.20 | 211.66 | 992.55 | -189.24 | 0.29 |  |
| **A+B+C** | **979.27** | **1,207.75** | **1,003.33** | **2,582.05** | **-24.06** | **0.96** | **-1,079.46** | **1,031.34** | **-557.56** | **1,643.23** | **-1,194.74** | **0.08** | **74.89** | **2,765.45** | **-1,042.10** | **0.89** |  |
| **D) Non-Mental Health Care** | **239.34** | **465.76** | **531.10** | **1,464.85** | **-291.76** | **0.28** | **-819.61** | **236.10** | **-735.60** | **1,337.49** | **-1,254.23** | **0.01** | **94.49** | **1,293.06** | **-427.78** | **0.71** |  |
| Primary Care | 95.88 | 183.16 | 38.06 | 72.01 | 57.82 | 0.12 | -15.22 | 130.86 | -156.42 | 202.11 | -234.79 | 0.00 | -232.97 | 190.79 | -310.03 | 0.00 |  |
| NMH Community Care | 73.88 | 292.26 | 52.55 | 154.55 | 21.33 | 0.73 | -97.88 | 140.54 | -213.73 | 752.70 | -505.60 | 0.14 | -30.12 | 374.95 | -181.57 | 0.69 |  |
| NMH Outpatient Care | 53.33 | 103.47 | 51.40 | 154.57 | 1.93 | 0.96 | -68.21 | 72.06 | -264.96 | 693.75 | -533.97 | 0.05 | -31.50 | 295.90 | -151.02 | 0.59 |  |
| NMH Inpatient Care | 16.25 | 62.46 | 389.09 | 1,435.15 | -372.84 | 0.20 | -937.06 | 191.39 | -100.49 | 434.31 | -268.90 | 0.23 | 389.09 | 1,435.15 | -190.58 | 0.18 |  |
| **Perspective I: A+B+C+D  (HEALTH CARE)** | **1,218.61** | **1,361.31** | **1,534.43** | **3,870.34** | **-315.82** | **0.67** | **-1,745.74** | **1,114.10** | **-1,293.16** | **2,625.82** | **-2,311.35** | **0.01** | **169.38** | **3,806.28** | **-1,368.01** | **0.82** |  |
| **E) Social Care** | **35.99** | **98.11** | **20.78** | **89.66** | **15.21** | **0.59** | **-40.42** | **70.84** | **-26.83** | **190.71** | **-100.78** | **0.46** | **-82.85** | **378.91** | **-235.90** | **0.28** |  |
| **Perspective II: A+B+C+D+E  (HEALTH AND SOCIAL CARE)** | **1,254.60** | **1,357.24** | **1,555.21** | **3,899.76** | **-300.61** | **0.68** | **-1,743.35** | **1,142.13** | **-1,319.99** | **2,681.30** | **-2,359.69** | **0.01** | **86.53** | **3,876.38** | **-1,479.17** | **0.91** |  |
| **F) Indirect Costs** | **7,818.58** | **9,844.23** | **7,384.70** | **10,400.92** | **433.88** | **0.88** | **-4,977.14** | **5,844.90** | **-5,169.89** | **-8,985.48** | **-1,354.29** | **0.01** | **-3,682.75** | **-7,323.27** | **-42.23** | **0.05** |  |
| Informal Care | 283.24 | 550.87 | 69.81 | 142.74 | 213.43 | 0.05 | -2.44 | 429.31 | -1.00 | 750.32 | -291.94 | 0.99 | -35.41 | 228.36 | -127.65 | 0.44 |  |
| Transport Expenses | 1.69 | 2.46 | 1.24 | 2.65 | 0.45 | 0.54 | -0.98 | 1.88 | -3.78 | 6.31 | -6.22 | 0.00 | -3.43 | 9.56 | -7.29 | 0.08 |  |
| Lost Household Productivity | 2,638.20 | 2,957.28 | 2,084.56 | 2,287.15 | 553.64 | 0.44 | -849.42 | 1,956.70 | -2,362.50 | 3,404.25 | -3,682.53 | 0.00 | -1,000.27 | 2,414.98 | -1,975.70 | 0.04 |  |
| Lost Productivity Costs | 4,895.45 | 8,480.20 | 5,229.09 | 9,905.47 | -333.64 | 0.89 | -5,243.04 | 4,575.76 | -2,802.61 | -6,278.61 | 673.39 | 0.11 | -2,643.64 | -5,991.64 | 704.37 | 0.12 |  |
| Absenteeism Cost | 3,961.62 | 7,012.86 | 3,780.55 | 7,711.31 | 181.07 | 0.93 | -3,756.78 | 4,118.92 | -2,011.24 | -5,388.51 | 1,366.03 | 0.23 | -1,803.98 | -4,632.50 | 1,024.55 | 0.20 |  |
| Presenteeism Cost | 933.83 | 2,582.31 | 1,448.54 | 4,308.17 | -514.71 | 0.59 | -2,394.73 | 1,365.31 | -791.37 | -1,683.71 | 100.97 | 0.08 | -839.66 | -1,683.88 | 4.56 | 0.05 |  |
| **Perspective III: A+B+C+D+E+F (SOCIETAL)** | **9,073.18** | **10,451.16** | **8,939.90** | **11,164.46** | **133.27** | **0.96** | **-5,634.92** | **5,901.47** | **-6,489.88** | **-10,690.76** | **-2,288.99** | **0.00** | **-3,596.22** | **-7,786.22** | **593.78** | **0.09** |  |

Note: n = sample size. SD = standard deviation. PReDicT = PReDicT intervention group, TAU = Treatment-As-Usual group. .. = not available. LCL = lower confidence level. UCL = upper confidence level.  ^1^ Mean diff = mean(PReDiCT) – mean(TAU). Multiple imputation included treatment group, age, sex, country, and baseline costs as predictors. Means costs by group and the adjusted difference were estimated using a generalized linear model (GLM) with family gamma and link log (cost as the dependent variable and treatment group as the independent variable), using the margins command. ^2^ Mean diff = mean(DURING trial costs) – mean(BEFORE Trial costs); ttest.

## Supplementary Table S11: Mean costs per participant - Spain (€, year 2018, n=164)

|  | **DURING Trial (week 0 to week 24)** | | | | | | | | **BEFORE Trial vs. DURING Trial costs** | | | | | | | | |
| --- | --- | --- | --- | --- | --- | --- | --- | --- | --- | --- | --- | --- | --- | --- | --- | --- | --- |
|  | **PReDicT (n=82)** | | **TAU (n=82)** | |  |  |  |  | **PReDicT (n=82)** | | | | **TAU (n=82)** | | | | |
|  | **Mean** | **SD** | **Mean** | **SD** | **Mean diff^1^** | **p-value** | **95% LCL** | **95% UCL** | **Mean diff^2^** | **95% LCL** | **95% UCL** | **p-value** | **Mean diff^2^** | **95% LCL** | **95% UCL** | **p-value** |  |
| **A) Intervention (PReDicT test) costs** | **122.34** | **304.62** | **0.00** | **0.00** | **122.34** | **0.00** | **55.91** | **188.77** | **122.34** | **55.91** | **188.77** | **0.00** | **.** | **.** | **.** | **.** |  |
| **B) Total medication costs** | **26.44** | **50.43** | **24.50** | **34.59** | **1.94** | **0.77** | **-11.16** | **15.03** | **18.23** | **14.00** | **22.46** | **0.00** | **18.85** | **14.92** | **22.79** | **0.00** |  |
| Antidepressants (BNF chapter 04.03) | 17.27 | 18.97 | 17.15 | 22.31 | 0.12 | 0.97 | -6.23 | 6.46 | 17.21 | 13.05 | 21.36 | 0.00 | 15.54 | 12.30 | 18.78 | 0.00 |  |
| Other MH (BNF chapter 04.01/02/04) | 2.04 | 3.05 | 2.50 | 3.44 | -0.46 | 0.37 | -1.46 | 0.55 | 0.94 | 0.37 | 1.51 | 0.00 | 1.47 | 0.86 | 2.08 | 0.00 |  |
| Other Nervous System (BNF chapter 04.05-11) | 7.13 | 47.38 | 4.85 | 17.92 | 2.28 | 0.66 | -7.76 | 12.31 | 0.08 | -0.05 | 0.22 | 0.22 | 1.84 | -0.09 | 3.76 | 0.06 |  |
| **C) Mental Health Care** | **119.73** | **652.51** | **59.98** | **208.94** | **59.75** | **0.38** | **-72.84** | **192.33** | **-37.06** | **-208.99** | **134.88** | **0.67** | **-24.49** | **-92.46** | **43.48** | **0.48** |  |
| MH Community Care | 101.98 | 641.37 | 26.36 | 57.11 | 75.62 | 0.17 | -31.62 | 182.86 | -44.03 | -211.24 | 123.18 | 0.60 | -36.55 | -77.65 | 4.54 | 0.08 |  |
| MH Outpatient Care | 7.34 | 31.53 | 26.51 | 172.49 | -19.17 | 0.25 | -51.99 | 13.66 | -3.44 | -17.55 | 10.67 | 0.63 | 4.94 | -39.21 | 49.10 | 0.82 |  |
| MH Inpatient Care | 10.41 | 46.82 | 7.12 | 27.85 | 3.29 | 0.58 | -8.21 | 14.80 | 10.41 | 0.12 | 20.70 | 0.05 | 7.12 | 1.00 | 13.24 | 0.02 |  |
| **A+B+C** | **268.51** | **709.10** | **84.49** | **215.09** | **184.03** | **0.02** | **25.89** | **342.16** | **103.51** | **-78.71** | **285.73** | **0.26** | **-5.64** | **-74.73** | **63.45** | **0.87** |  |
| **D) Non-Mental Health Care** | **109.69** | **196.18** | **299.57** | **851.38** | **-189.88** | **0.02** | **-353.83** | **-25.93** | **-807.61** | **-1,018.28** | **-596.94** | **0.00** | **-712.18** | **-983.51** | **-440.85** | **0.00** |  |
| Primary Care | 28.24 | 54.51 | 24.97 | 50.37 | 3.27 | 0.69 | -12.83 | 19.38 | -342.01 | -397.36 | -286.66 | 0.00 | -394.11 | -459.38 | -328.85 | 0.00 |  |
| NMH Community Care | 16.07 | 65.39 | 39.71 | 142.40 | -23.65 | 0.19 | -59.22 | 11.93 | -195.73 | -324.57 | -66.88 | 0.00 | -128.77 | -250.96 | -6.58 | 0.04 |  |
| NMH Outpatient Care | 38.41 | 97.78 | 154.89 | 528.51 | -116.48 | 0.03 | -220.47 | -12.50 | -251.31 | -370.61 | -132.02 | 0.00 | -254.74 | -431.65 | -77.83 | 0.01 |  |
| NMH Inpatient Care | 26.98 | 82.42 | 80.01 | 490.92 | -53.02 | 0.24 | -141.61 | 35.56 | -18.55 | -79.65 | 42.54 | 0.55 | 65.44 | -46.76 | 177.64 | 0.25 |  |
| **Perspective I: A+B+C+D  (HEALTH CARE)** | **378.21** | **779.99** | **384.06** | **903.41** | **-5.86** | **0.97** | **-264.03** | **252.32** | **-704.09** | **-973.19** | **-435.00** | **0.00** | **-717.82** | **-996.43** | **-439.21** | **0.00** |  |
| **E) Social Care** | **25.64** | **95.59** | **105.13** | **635.66** | **-79.49** | **0.19** | **-197.16** | **38.18** | **-63.38** | **-207.85** | **81.08** | **0.39** | **88.79** | **-52.29** | **229.88** | **0.21** |  |
| **Perspective II: A+B+C+D+E  (HEALTH AND SOCIAL CARE)** | **403.84** | **786.65** | **489.19** | **1,261.87** | **-85.34** | **0.59** | **-399.47** | **228.78** | **-767.48** | **-1,076.29** | **-458.67** | **0.00** | **-629.03** | **-981.47** | **-276.58** | **0.00** |  |
| **F) Indirect Costs** | **3,885.05** | **5,505.20** | **6,215.43** | **9,488.26** | **-2,330.39** | **0.05** | **-4,669.29** | **8.51** | **-1,882.13** | **-3,300.84** | **-463.42** | **0.01** | **-1,179.57** | **-3,324.99** | **965.86** | **0.28** |  |
| Informal Care | 892.92 | 2,569.98 | 1,947.53 | 6,632.87 | -1,054.62 | 0.16 | -2,516.76 | 407.52 | 252.17 | -188.73 | 693.07 | 0.26 | 612.35 | -846.21 | 2,070.92 | 0.41 |  |
| Transport Expenses | 1.07 | 4.35 | 1.07 | 2.52 | 0.00 | 1.00 | -1.09 | 1.09 | -4.43 | -6.88 | -1.99 | 0.00 | -4.82 | -8.07 | -1.56 | 0.00 |  |
| Lost Household Productivity | **1,089.87** | **1,107.95** | **1,061.40** | **1,067.49** | **28.47** | **0.87** | **-304.59** | **361.54** | **-826.66** | **-1,135.35** | **-517.98** | **0.00** | **-893.78** | **-1,185.23** | **-602.32** | **0.00** |  |
| Lost Productivity Costs | **1,901.19** | **4,410.64** | 3,205.43 | **5,230.29** | **-1,304.23** | **0.11** | **-2,922.73** | **314.26** | **-1,303.21** | **-2,486.94** | **-119.48** | **0.03** | **-893.33** | **-2,191.72** | **405.06** | **0.17** |  |
| Absenteeism Cost | 1,579.01 | 4,193.59 | 2,796.19 | 4,991.24 | -1,217.19 | 0.13 | -2,789.87 | 355.50 | -632.35 | -1,742.38 | 477.67 | 0.26 | -148.24 | -1,385.01 | 1,088.52 | 0.81 |  |
| Presenteeism Cost | 322.19 | 1,028.99 | 409.24 | 1,221.34 | -87.05 | 0.62 | -435.53 | 261.43 | -670.86 | -1,158.46 | -183.26 | 0.01 | -745.08 | -1,141.58 | -348.59 | 0.00 |  |
| **Perspective III: A+B+C+D+E+F (SOCIETAL)** | **4,288.89** | **5,553.46** | **6,704.62** | **9,706.91** | **-2,415.72** | **0.05** | **-4,783.96** | **-47.48** | **-2,649.61** | **-4,135.25** | **-1,163.97** | **0.00** | **-1,808.59** | **-4,078.59** | **461.40** | **0.12** |  |

Note: n = sample size. SD = standard deviation. PReDicT = PReDicT intervention group, TAU = Treatment-As-Usual group. .. = not available. LCL = lower confidence level. UCL = upper confidence level.  ^1^ Mean diff = mean(PReDiCT) – mean(TAU). Multiple imputation included treatment group, age, sex, country, and baseline costs as predictors. Means costs by group and the adjusted difference were estimated using a generalized linear model (GLM) with family gamma and link log (cost as the dependent variable and treatment group as the independent variable), using the margins command. ^2^ Mean diff = mean(DURING trial costs) – mean(BEFORE Trial costs); ttest.

## Supplementary Table S12: Mean costs per participant - United Kingdom (€, year 2018, n=489)

|  | **DURING Trial (week 0 to week 24)** | | | | | | | | | **BEFORE Trial vs. DURING Trial costs** | | | | | | | |
| --- | --- | --- | --- | --- | --- | --- | --- | --- | --- | --- | --- | --- | --- | --- | --- | --- | --- |
|  | **PReDicT (n=248)** | | **TAU (n=241)** | | **Mean diff^1^** |  |  |  |  | **PReDicT (n=248)** | | | | **TAU (n=241)** | | | |
|  | **Mean** | **SD** | **Mean** | **SD** |  |  | **p-value** | **95% LCL** | **95% UCL** | **Mean diff^2^** | **95% LCL** | **95% UCL** | **p-value** | **Mean diff^2^** | **95% LCL** | **95% UCL** | **p-value** |
| **A) Intervention (PReDicT test) costs** | **77.96** | **216.59** | **0.00** | **0.00** | **77.96** |  | **0.00** | **50.54** | **105.37** | **77.96** | **50.54** | **105.37** | **0.00** | **.** | **.** | **.** | **.** |
| **B) Total medication costs** | **15.19** | **27.90** | **18.70** | **59.70** | **-3.51** |  | **0.38** | **-11.36** | **4.33** | **8.96** | **4.91** | **13.01** | **0.00** | **11.33** | **8.20** | **14.47** | **0.00** |
| Antidepressants (BNF chapter 04.03) | 10.29 | 12.17 | 11.22 | 19.50 | -0.92 |  | 0.52 | -3.75 | 1.91 | 9.91 | 8.44 | 11.37 | 0.00 | 10.62 | 8.16 | 13.07 | 0.00 |
| Other MH (BNF chapter 04.01/02/04) | 0.58 | 5.91 | 0.15 | 0.69 | 0.44 |  | 0.15 | -0.16 | 1.03 | 0.47 | -0.24 | 1.18 | 0.20 | 0.11 | 0.04 | 0.18 | 0.00 |
| Other Nervous System (BNF chapter 04.05-11) | 4.31 | 25.09 | 7.34 | 56.66 | -3.03 |  | 0.42 | -10.33 | 4.28 | -1.42 | -5.07 | 2.23 | 0.44 | 0.61 | -1.42 | 2.64 | 0.55 |
| **C) Mental Health Care** | **73.75** | **250.00** | **81.89** | **279.80** | **-8.15** |  | **0.73** | **-55.20** | **38.91** | **-242.05** | **-376.23** | **-107.86** | **0.00** | **-363.75** | **-675.16** | **-52.33** | **0.02** |
| MH Community Care | 35.33 | 136.51 | 32.71 | 130.42 | 2.62 |  | 0.83 | -21.06 | 26.30 | -261.29 | -388.64 | -133.95 | 0.00 | -223.11 | -350.88 | -95.33 | 0.00 |
| MH Outpatient Care | 6.02 | 30.85 | 33.84 | 206.92 | -27.81 |  | 0.03 | -52.24 | -3.38 | -13.15 | -42.26 | 15.97 | 0.37 | -16.65 | -72.07 | 38.76 | 0.55 |
| MH Inpatient Care | 32.39 | 158.98 | 15.35 | 65.35 | 17.05 |  | 0.10 | -3.53 | 37.62 | 32.39 | 12.51 | 52.28 | 0.00 | -123.99 | -398.71 | 150.74 | 0.37 |
| **A+B+C** | **166.89** | **334.51** | **100.59** | **283.75** | **66.30** |  | **0.03** | **6.85** | **125.74** | **-155.13** | **-287.44** | **-22.82** | **0.02** | **-352.41** | **-663.89** | **-40.94** | **0.03** |
| **D) Non-Mental Health Care** | **137.82** | **620.34** | **199.46** | **868.24** | **-61.64** |  | **0.37** | **-196.59** | **73.31** | **-281.15** | **-441.69** | **-120.60** | **0.00** | **-229.53** | **-410.09** | **-48.97** | **0.01** |
| Primary Care | 5.09 | 16.21 | 8.09 | 34.51 | -3.00 |  | 0.19 | -7.51 | 1.52 | -73.30 | -91.16 | -55.43 | 0.00 | -70.20 | -90.25 | -50.14 | 0.00 |
| NMH Community Care | 10.82 | 76.31 | 9.98 | 52.26 | 0.84 |  | 0.89 | -10.64 | 12.32 | -82.25 | -167.18 | 2.68 | 0.06 | -32.31 | -61.87 | -2.75 | 0.03 |
| NMH Outpatient Care | 35.56 | 161.18 | 43.61 | 185.15 | -8.05 |  | 0.61 | -39.09 | 22.99 | -108.56 | -165.81 | -51.31 | 0.00 | -153.51 | -241.95 | -65.07 | 0.00 |
| NMH Inpatient Care | 86.35 | 513.25 | 137.78 | 775.50 | -51.44 |  | 0.40 | -169.84 | 66.97 | -17.04 | -134.20 | 100.12 | 0.77 | 26.48 | -110.70 | 163.67 | 0.70 |
| **Perspective I: A+B+C+D  (HEALTH CARE)** | **304.71** | **714.08** | **300.05** | **1,010.27** | **4.66** |  | **0.95** | **-150.45** | **159.75** | **-436.28** | **-645.00** | **-227.55** | **0.00** | **-581.94** | **-993.83** | **-170.06** | **0.01** |
| **E) Social Care** | **10.49** | **92.32** | **5.92** | **29.39** | **4.57** |  | **0.41** | **-6.22** | **15.36** | **8.02** | **-4.04** | **20.07** | **0.19** | **-42.08** | **-111.20** | **27.03** | **0.23** |
| **Perspective II: A+B+C+D+E  (HEALTH AND SOCIAL CARE)** | **315.20** | **721.64** | **305.97** | **1,013.84** | **9.23** |  | **0.91** | **-147.17** | **165.62** | **-428.26** | **-637.91** | **-218.62** | **0.00** | **-624.03** | **-1,041.35** | **-206.71** | **0.00** |
| **F) Indirect Costs** | **4,412.74** | **5,657.75** | **4,485.03** | **5,289.97** | **-72.29** |  | **0.88** | **-1,045.02** | **900.45** | **-1,543.54** | **-2,287.82** | **-799.26** | **0.00** | **-2,032.60** | **-3,280.88** | **-784.33** | **0.00** |
| Informal Care | 339.09 | 1,649.30 | 250.47 | 674.86 | 88.62 |  | 0.40 | -119.53 | 296.78 | -280.44 | -588.48 | 27.60 | 0.07 | -682.80 | -1,726.59 | 361.00 | 0.20 |
| Transport Expenses | 0.55 | 2.29 | 0.48 | 2.09 | 0.07 |  | 0.74 | -0.32 | 0.45 | -5.77 | -7.54 | -4.01 | 0.00 | -3.66 | -4.87 | -2.45 | 0.00 |
| Lost Household Productivity | **2,041.15** | **1,892.50** | **2,125.02** | **1,881.14** | **-83.87** |  | **0.62** | **-418.91** | **251.17** | **-530.39** | **-717.06** | **-343.72** | **0.00** | **-348.91** | **-550.64** | **-147.19** | **0.00** |
| Lost Productivity Costs | **2,031.96** | **4,440.69** | **2,109.05** | **4,156.85** | **-77.10** |  | **0.84** | **-841.85** | **687.65** | **-726.94** | **-1,284.26** | **-169.62** | **0.01** | **-997.23** | **-1,563.18** | **-431.29** | **0.00** |
| Absenteeism Cost | 1,465.14 | 3,732.91 | 1,661.03 | 3,718.02 | -195.89 |  | 0.57 | -862.95 | 471.16 | 172.23 | -259.50 | 603.96 | 0.43 | 7.66 | -490.95 | 506.28 | 0.98 |
| Presenteeism Cost | 566.82 | 1,881.31 | 448.02 | 1,445.05 | 118.79 |  | 0.43 | -177.20 | 414.79 | -899.16 | -1,191.47 | -606.86 | 0.00 | -1,004.90 | -1,317.11 | -692.69 | 0.00 |
| **Perspective III: A+B+C+D+E+F (SOCIETAL)** | **4,727.94** | **5,826.01** | **4,791.00** | **5,519.81** | **-63.06** |  | **0.90** | **-1,070.46** | **944.34** | **-1,971.80** | **-2,764.13** | **-1,179.47** | **0.00** | **-2,656.63** | **-3,982.31** | **-1,330.95** | **0.00** |

Note: n = sample size. SD = standard deviation. PReDicT = PReDicT intervention group, TAU = Treatment-As-Usual group. .. = not available. LCL = lower confidence level. UCL = upper confidence level.  ^1^ Mean diff = mean(PReDiCT) – mean(TAU). Multiple imputation included treatment group, age, sex, country, and baseline costs as predictors. Means costs by group and the adjusted difference were estimated using a generalized linear model (GLM) with family gamma and link log (cost as the dependent variable and treatment group as the independent variable), using the margins command. ^2^ Mean diff = mean(DURING trial costs) – mean(BEFORE Trial costs); ttest.

## Supplementary Table S13: Net monetary benefit of PReDicT in comparison to TAU (n=913)

| **Perspective** | **WTP threshold = €34,000/QALY** | | **WTP threshold = €50,000/QALY** | |
| --- | --- | --- | --- | --- |
|  | NMB | Probability PReDicT being cost-effective | NMB | Probability PReDicT being cost-effective |
|  | (95% CI) |  | (95% CI) |  |
| **I: Health care** | -€ 35 | 46% | € 43 | 53% |
|  | (-€1,215 to €1,165) |  | (-€1,340 to €1,406) |  |
| **II: Health and social care** | -€ 38 | 48% | € 27 | 53% |
|  | (-€1,227 to €1,173) |  | (-€1,375 to €1,406) |  |
| **III: Societal** | € 148 | 56% | € 237 | 59% |
|  | (-€2,405 to €2,382) |  | (-€2,543 to €2,735) |  |

Note: n = sample size. WTP: Willingness-To-Pay, NMB: Net Monetary Benefit, CI: Confidence Interval.

## Supplementary Table S14: Cost-effectiveness of PReDicT from different analytical perspectives - United Kingdom (€, year 2018, n=489)

| **Outcome** | **Cost** | **Outcome difference ^1^ (95% CI)** | **ICER** | **CE plane** | | | | **WTP threshold = €34,000/QALY** | | **WTP threshold = €50,000/QALY** | |
| --- | --- | --- | --- | --- | --- | --- | --- | --- | --- | --- | --- |
|  | **difference ^1^** |  | **(95% CI) ^1^** |  |  |  |  |  |  |  |  |
|  | **(95% CI)** |  |  | **NE** | **SE** | **SW** | **NW** | **NMB (95% CI)** | **Probability PReDicT being cost-effective** | **NMB (95% CI)** | **Probability PReDicT being cost-effective** |
| **I: Health care perspective** | | | | | | | | | | | |
| HRQoL | **€ 4.66** (-€150.45 to €159.75) | **0.0069** | **€677/QALY** | 40% | 43% | 8% | 9% | €227 (-€281 to €697) | 82% | €331  (-€358 to €993) | 83% |
| (EQ-5D-5L) |  | (-0.0064 to 0.0202) | (-€87,561/QALY to €94,626/QALY) |  |  |  |  |  |  |  |  |
| Capability |  | **0.0037** | **€1,255/CWLY** | 38% | 37% | 13% | 13% | n/a | n/a | n/a | n/a |
| (OxCAP-MH) |  | (-0.0063 to 0.0137) | (-€154,006/CWLY to €197,494/CWLY) |  |  |  |  |  |  |  |  |
| **II: Health and social care perspective** | | | | | | | | | | | |
| HRQoL | **€ 9.23** (-€147.17 to €165.62) | **0.0069** | **€1,342/QALY** | 46% | 38% | 5% | 10% | €216  (-€298 to €690) | 81% | €321  (-€393 to €984) | 82% |
| (EQ-5D-5L) |  | (-0.0064 to 0.0202) | (-€88,654/QALY to €110,075/QALY) |  |  |  |  |  |  |  |  |
| Capability |  | **0.0037** | **€2,488/CWLY** | 38% | 38% | 10% | 14% | n/a | n/a | n/a | n/a |
| (OxCAP-MH) |  | (-0.0063 to 0.0137) | (-€167,195/CWLY to €150,879/CWLY) |  |  |  |  |  |  |  |  |
| **III: Societal perspective** | | | | | | | | | | | |
| HRQoL | **-€63.06** (-€1,070.46 to €944.34) | **0.0069** | **-€9,172/QALY** (-€708,707 to €648,084) | 28% | 56% | 5% | 11% | €388  (-€903 to €1,756) | 71% | €50  (-€948 to €1,997) | 74% |
| (EQ-5D-5L) |  | (-0.0064 to 0.0202) |  |  |  |  |  |  |  |  |  |
| Capability |  | **0.0037** | **-€17,005/CWLY** (-€1,392,331 to €839,516) | 25% | 49% | 10% | 16% | n/a | n/a | n/a | n/a |
| (OxCAP-MH) |  | (-0.0063 to 0.0137) |  |  |  |  |  |  |  |  |  |

Note: n= sample size. QALYs = Quality-Adjusted Life Years. CWLY = Capability-Weighted Life Years. PReDicT = PReDicT intervention group. TAU = Treatment-As-Usual group. CI = confidence interval. ICER = incremental cost-effectiveness ratio.
^1^ difference = mean(PReDiCT) - mean(TAU); multiple imputation included treatment group, age, sex, baseline values (baseline EQ-5D for HRQoL, OxCAP-MH index for capability outcomes, or baseline costs) as predictors. Differences were estimated using regression models with treatment group as the explanatory variable (costs: generalized linear model with gamma family and log link; outcomes: multilevel mixed-effects linear regression) with treatment group as the explanatory variable.

## Supplementary Table S15: QALY and CWLY under alternative timepoint assumptions

|  | **n** | **Mean difference^1^** | **95% LCL** | **95% UCL** | **p-value** |  |
| --- | --- | --- | --- | --- | --- | --- |
| QALY (mid) | 913 | 0.0039 | -0.0051 | 0.0128 | 0.3970 |  |
| QALY (begin) | 913 | 0.0038 | -0.0052 | 0.0129 | 0.4090 |  |
| QALY (end) | 913 | 0.0039 | -0.0050 | 0.0128 | 0.3920 |  |
| CWLY (mid) | 619 | 0.0056 | -0.0034 | 0.0146 | 0.2230 |  |
| CWLY (begin) | 619 | 0.0084 | -0.0009 | 0.0178 | 0.0780 |  |
| CWLY (end) | 619 | 0.0028 | -0.0063 | 0.0118 | 0.5460 |  |

Note: n = sample size. QALYs = Quality Adjusted Life Years (based on EQ-5D-5L DE 5L tariffs). CWLY = Capability Weighted Life Year. Mean diff = difference: mean(PReDiCT) – mean(TAU). PReDicT = PReDicT intervention group, TAU = Treatment-As-Usual group. LCL = lower confidence level. UCL = upper confidence level.
^1^ difference = mean(PReDiCT) - mean(TAU); multiple imputation included treatment group, age, sex, baseline values (baseline EQ-5D for HRQoL, OxCAP-MH index for capability outcomes) as predictors. Differences were estimated using multilevel mixed-effects linear regression with treatment group as the explanatory variable (*p<0.05).

## Supplementary Table S16: Cost-effectiveness of PReDicT from different analytical perspectives, assuming outcome changes at the start of each timepoint

| **Outcome** | **n** | **Cost  difference ^1^**  **(95% CI)** | **Outcome difference ^2^ (95% CI)** | **ICER**  **(95% CI)** | **CE plane** | | | |
| --- | --- | --- | --- | --- | --- | --- | --- | --- |
|  |  |  |  |  | **NE** | **SE** | **SW** | **NW** |
|  | **I: Health care perspective** | | | | | | | |
| HRQoL | 913 | **210.83 €** | **0,0038** | **€55,200/QALY** | 43% | 25% | 10% | 22% |
| (EQ-5D-5L) |  | (-230.53 to 652.18) | (-0.0052 to 0.0129) | (-€530,913/QALY to €760,511/QALY) |  |  |  |  |
| Capability | 619 | **376.73 €** | **0,0084** | **€44,792/CWLY** | 91% | 6% | 0% | 3% |
| (OxCAP-MH) |  | (-122.25 to 875.71) | (-0.0009 to 0.0178) | (-€73,136/CWLY to €426,984/CWLY) |  |  |  |  |
|  | **II: Health and social care perspective** | | | | | | | |
| HRQoL | 913 | **209.60 €** | **0,0038** | **€54,879/QALY** | 44% | 22% | 11% | 23% |
| (EQ-5D-5L) |  | (-233.83 to 653.03) | (-0.0052 to 0.0129) | (-€477,473/QALY to €495,928/QALY) |  |  |  |  |
| Capability | 619 | **392.51 €** | **0,0084** | **€46,668/CWLY** | 91% | 6% | 0% | 3% |
| (OxCAP-MH) |  | (-108.79 to 893.81) | (-0.0009 to 0.0178) | (-€124,955/CWLY to €325,799/CWLY) |  |  |  |  |
|  | **III: SOCIETAL perspective** | | | | | | | |
| HRQoL | 913 | **€ 99.19** | **0,0038** | **€25,970/QALY** | 33% | 35% | 10% | 22% |
| (EQ-5D-5L) |  | (-€880.30 to €1,078.67) | (-0.0052 to 0.0129) | (-€984,995/QALY to €908,046/QALY) |  |  |  |  |
| Capability | 619 | **€ 658.90** | **0,0084** | **€78,341/CWLY** | 83% | 14% | 0% | 4% |
| (OxCAP-MH) |  | (-€463.85 to €1,781.65) | (-0.0009 to 0.0178) | (-€251,904/CWLY to €725,622/CWLY) |  |  |  |  |

Note: n = sample size. QALYs = Quality-Adjusted Life Years. CWLY = Capability-Weighted Life Years. PReDicT = PReDicT intervention group. TAU = Treatment-As-Usual group. CI = confidence interval. ICER = incremental cost-effectiveness ratio.
^1^ difference = mean(PReDiCT) - mean(TAU); multiple imputation included treatment group, age, sex, baseline values (baseline EQ-5D for HRQoL, OxCAP-MH index for capability outcomes, or baseline costs) as predictors. Differences were estimated using regression models with treatment group as the explanatory variable (costs: generalized linear model with gamma family and log link; outcomes: multilevel mixed-effects linear regression) with treatment group as the explanatory variable.

## Supplementary Table S17: Health economic outcomes over time - complete cases (n=534)

|  | week 24 versus week 0 | | | | | | | | | | | | | | | | | |
| --- | --- | --- | --- | --- | --- | --- | --- | --- | --- | --- | --- | --- | --- | --- | --- | --- | --- | --- |
|  | **Trial level** | | | **France** | | | **Germany** | | | **Netherlands** | | | **Spain** | | | **United Kingdom** | | |
|  | n | Mean diff | p-value^1^ | n | Mean diff | p-value^1^ | n | Mean diff | p-value^1^ | n | Mean diff | p-value^1^ | n | Mean diff | p-value^1^ | n | Mean diff | p-value^1^ |
| EQ-5D-5L | 534 |  |  | 65 |  |  | 62 |  |  | 38 |  |  | 100 |  |  | 269 |  |  |
| *EQ-5D tariff* | *5L DE* | | | *Crosswalk 3L FR* | | | *5L DE* | | | *5L NL* | | | *5L ES* | | | *Crosswalk 3L UK* | | |
| PReDiCT | 274 | 0.141 | **<0.001** | 32 | 0.1975 | **<0.001** | 31 | 0.1287 | **<0.001** | 20 | 0.1321 | **0.0030** | 52 | 0.1619 | **<0.001** | 139 | 0.1577 | **<0.001** |
| TAU | 260 | 0.146 | **<0.001** | 33 | 0.2499 | **<0.001** | 31 | 0.1712 | **<0.001** | 18 | 0.1627 | 0.0670 | 48 | 0.1275 | **<0.001** | 130 | 0.1566 | **<0.001** |
| Mean diff^2^ |  | -0.005 | 0.7660 |  | -0.0524 | 0.2970 |  | -0.0425 | 0.4020 |  | -0.0306 | 0.7360 |  | 0.0345 | 0.2880 |  | 0.0012 | 0.9640 |
| EQ-5D VAS | 534 |  |  | 65 |  |  | 62 |  |  | 38 |  |  | 100 |  |  | 269 |  |  |
| PReDiCT | 274 | 16.380 | **<0.001** | 32 | 23.2813 | **<0.001** | 31 | 9.1613 | **0.0110** | 20 | 15.5500 | **0.0010** | 52 | 12.4808 | **<0.001** | 139 | 17.9784 | **<0.001** |
| TAU | 260 | 14.027 | **<0.001** | 33 | 22.4242 | **<0.001** | 31 | 13.2903 | **0.0040** | 18 | 12.1111 | **0.0040** | 48 | 8.1875 | **0.0120** | 130 | 14.4923 | **<0.001** |
| Mean diff^2^ |  | 2.353 | 0.2520 |  | 0.8570 | 0.8770 |  | -4.1290 | 0.4820 |  | 3.4389 | 0.5820 |  | 4.2933 | 0.3170 |  | 3.4861 | 0.2530 |
| OXCAP-MH^3^ | 330 |  |  |  |  |  | 62 |  |  |  |  |  |  |  |  | 269 |  |  |
| PReDiCT | 170 | 10.453 | **<0.001** | .. | .. | .. | 31 | 5.8387 | **0.0090** | .. | .. | .. | .. | .. | .. | 139 | 11.4820 | **<0.001** |
| TAU | 160 | 9.238 | **<0.001** | .. | .. | .. | 31 | 9.0645 | **<0.001** | .. | .. | .. | .. | .. | .. | 129 | 9.2791 | **<0.001** |
| Mean diff^2^ |  | 1.215 | 0.3810 | .. | .. | .. |  | -3.2258 | 0.2840 | .. | .. | .. | .. | .. | .. |  | 2.2029 | 0.1560 |

Note: n = sample size. SD = standard deviation. PReDicT = PReDicT intervention group, TAU = Treatment-As-Usual group. VAS = Visual Analogue Scale. FR = France, DE = Germany, NL = The Netherlands, ES = Spain, UK = United Kingdom. .. = not available.
^1^ Multilevel mixed-effects linear regression according to treatment group and week, p<0.05. ^2^ Mean diff = difference: mean(PReDiCT) – mean(TAU). ^3^ Data were only collected in UK and DE.

## Supplementary Table S18a: Health economic analysis sample characteristics– complete cases (n=534)

|  | **PReDicT (n=274)** | **TAU (n=260)** |
| --- | --- | --- |
| **Country, n (%)** |  |  |
| France | 32 (12%) | 33 (13%) |
| Germany | 31 (11%) | 31 (12%) |
| The Netherlands | 20 (7%) | 18 (7%) |
| Spain | 52 (19%) | 48 (18%) |
| United Kingdom | 139 (51%) | 130 (50%) |
| **Mean age at baseline (SD)** | 40.84  (13.57) | 40.55 (14.37) |
| **Female sex, n (%)** | 173 (63%) | 174 (67%) |
| **Ethnicity*, n (%)** |  |  |
| White | 212 (77%) | 211 (81%) |
| Racially minoritized groups* | 62 (23%) | 49 (19%) |
| **(Self-)Employed during trial, n (%)** |  |  |
| **Yes** | 197 (72%) | 176 (68%) |
| **No** | 77 (28%) | 84 (32%) |
| Working age (18-65 years) | 68 (88%) | 77 (92%) |
| Past retirement age (>65 years) | 9 (12%) | 7 (8%) |
| **Mean length of education at baseline, years (SD)** | 14.44 (3.82) | 14.29 (3.47) |
| **Family history of depression, n (%)** |  |  |
| No | 142 (52%) | 121 (47%) |
| Yes | 132 (48%) | 139 (54%) |
| **Mean length of depression at baseline, years (SD)** | 5.27 (8.36) | 5.15 (7.81) |
| **Mean baseline QIDS-SR-16 (SD)** | 15.40 (4.54) | 15.48 (4.26) |
| **Mean baseline EQ-5D-5L index (SD) ^1^** | 0.704 (0.24) | 0.703 (0.20) |
| **Mean baseline EQ VAS (SD)** | 50.71 (20.14) | 51.99 (20.02) |
| **Mean baseline OxCAP-MH score (SD) ^2^** | 62.61 (15.46) | 57.45 (9.51) |

Note: n = sample size. SD = standard deviation. PReDicT = PReDicT intervention group, TAU = Treatment-As-Usual group. * A local ethical requirement prevented the collection of data on ethnicity from patients in France. *Includes: American Indian or Alaska Native, Asian, Black or African American, Native Hawaiian or Other Pacific Islander, Other.
QIDS-SR-16 = Quick Inventory of Depressive Symptoms, 16-item self-report version (score range 0–27). ^1^ Based on Ludwig K. et al. (2018) German value set for the EQ-5D-5L. Pharmacoeconomics. ^2^ Data were only collected in the UK and DE: PReDicT (n=170) and TAU (n=160).

## Supplementary Table S18b: Mean costs per participant - complete cases (€, year 2018, n=534)

|  | **DURING Trial (week 0 to week 24)** | | | | | | | | | | **BEFORE Trial (extrapolated over 24 weeks)** | | | | | | | |
| --- | --- | --- | --- | --- | --- | --- | --- | --- | --- | --- | --- | --- | --- | --- | --- | --- | --- | --- |
|  | **PReDicT (n=274)** | | **TAU (n=260)** | |  |  | |  | |  | **PReDicT (n=274)** | | **TAU (n=260)** | |  |  |  |  |
|  | **Mean** | **SD** | **Mean** | **SD** | **Mean diff^1^** |  | **95% LCL** | | **95% UCL** | | **Mean** | **SD** | **Mean** | **SD** | **Mean diff^1^** |  | **95% LCL** | **95% UCL** |
| **A) Intervention (PReDicT test) costs** | **97.34** | **238.81** | **0.00** | **0.00** | **97.34** | ******* | **68.24** | | **126.43** | | **0.00** | **0.00** | **0.00** | **0.00** | **0.00** |  | **0.00** | **0.00** |
| **B) Total medication costs** | **36.19** | **130.00** | **42.50** | **132.40** | **-6.32** |  | **-28.62** | | **15.99** | | **12.53** | **84.64** | **10.72** | **61.69** | **1.80** |  | **-10.84** | **14.45** |
| Antidepressants (BNF chapter 04.03) | 17.31 | 38.88 | 24.41 | 108.97 | -7.10 |  | -20.87 | | 6.67 | | 0.25 | 2.50 | 0.63 | 7.73 | -0.37 |  | -1.34 | 0.59 |
| Other MH (BNF chapter 04.01/02/04) | 10.31 | 89.42 | 10.37 | 52.78 | -0.07 |  | -12.63 | | 12.50 | | 2.97 | 20.29 | 3.19 | 23.43 | -0.22 |  | -3.94 | 3.50 |
| Other Nervous System (BNF chapter 04.05-11) | 8.57 | 75.82 | 7.72 | 55.26 | 0.85 |  | -10.48 | | 12.18 | | 9.31 | 82.48 | 6.91 | 56.02 | 2.40 |  | -9.65 | 14.44 |
| **C) Mental Health Care** | **531.55** | **2,517.00** | **492.62** | **2,771.75** | **38.94** |  | **-410.76** | | **488.64** | | **801.67** | **1,619.32** | **909.32** | **2839.15** | **-107.65** |  | **-498.09** | **282.78** |
| MH Community Care | 176.53 | 733.67 | 95.89 | 392.58 | 80.65 |  | -20.15 | | 181.45 | | 625.23 | 1,366.62 | 573.71 | 1347.13 | 51.52 |  | -179.30 | 282.35 |
| MH Outpatient Care | 68.49 | 522.78 | 102.71 | 678.01 | -34.21 |  | -136.83 | | 68.40 | | 100.37 | 408.44 | 187.56 | 943.09 | -87.20 |  | -209.68 | 35.29 |
| MH Inpatient Care | 286.53 | 2,018.64 | 294.02 | 2,385.72 | -7.50 |  | -382.52 | | 367.53 | | 76.07 | 724.31 | 148.05 | 2103.51 | -71.98 |  | -336.74 | 192.78 |
| **A+B+C** | **665.08** | **2,570.92** | **535.12** | **2,789.59** | **129.96** |  | **-325.78** | | **585.70** | | **814.20** | **1,620.58** | **920.05** | **2843.29** | **-105.85** |  | **-496.79** | **285.09** |
| **D) Non-Mental Health Care** | **194.42** | **713.63** | **362.39** | **1,862.24** | **-167.97** |  | **-405.45** | | **69.51** | | **994.94** | **3,010.17** | **707.09** | **1731.62** | **287.86** |  | **-132.53** | **708.25** |
| Primary Care | 18.65 | 69.77 | 13.37 | 42.07 | 5.29 |  | -4.57 | | 15.14 | | 167.29 | 206.99 | 181.13 | 224.84 | -13.84 |  | -50.55 | 22.88 |
| NMH Community Care | 14.56 | 101.06 | 23.93 | 106.98 | -9.38 |  | -27.06 | | 8.31 | | 183.22 | 740.15 | 80.62 | 359.34 | 102.60 | ** | 2.85 | 202.35 |
| NMH Outpatient Care | 52.76 | 303.65 | 69.87 | 290.36 | -17.11 |  | -67.66 | | 33.45 | | 255.89 | 621.37 | 240.01 | 976.22 | 15.88 |  | -122.51 | 154.27 |
| NMH Inpatient Care | 108.45 | 576.14 | 255.22 | 1,775.30 | -146.77 |  | -368.83 | | 75.29 | | 388.54 | 2,527.13 | 205.33 | 1125.97 | 183.21 |  | -152.43 | 518.85 |
| **Perspective I: A+B+C+D  (HEALTH CARE)** | **859.50** | **2,695.14** | **897.51** | **3,848.10** | **-38.01** |  | **-600.46** | | **524.45** | | **1,809.14** | **3,453.42** | **1627.13** | **3636.35** | **182.01** |  | **-420.69** | **784.70** |
| **E) Social Care** | **18.01** | **114.00** | **34.49** | **296.68** | **-16.48** |  | **-54.32** | | **21.37** | | **39.26** | **262.74** | **55.43** | **529.52** | **-16.17** |  | **-86.69** | **54.35** |
| **Perspective II: A+B+C+D+E  (HEALTH AND SOCIAL CARE)** | **877.51** | **2,716.25** | **931.99** | **3,878.04** | **-54.48** |  | **-621.32** | | **512.36** | | **1,848.40** | **3,493.72** | **1682.56** | **3657.53** | **165.83** |  | **-442.09** | **773.76** |
| **F) Indirect Costs** | **3,924.37** | **5,275.84** | **3,926.03** | **5,853.32** | **-1.66** |  | **-946.83** | | **943.50** | | **6,432.70** | **7,201.57** | **5,975.99** | **6,778.11** | **456.72** |  | **-733.59** | **1,647.02** |
| Informal Care | 455.04 | 1,788.71 | 478.82 | 2,470.83 | -23.78 |  | -389.11 | | 341.55 | | 683.60 | 2,401.62 | 458.57 | 1538.65 | 225.03 |  | -119.87 | 569.92 |
| Transport Expenses | 5.10 | 46.27 | 1.87 | 7.90 | 3.23 |  | -2.49 | | 8.94 | | 17.13 | 72.73 | 13.58 | 47.04 | 3.56 |  | -6.91 | 14.03 |
| Lost Household Productivity | 1,465.36 | 1,474.76 | 1,528.68 | 1,468.44 | -63.32 |  | -313.62 | | 186.98 | | 2,421.89 | 1,756.83 | 2278.08 | 1773.92 | 143.80 |  | -156.41 | 444.02 |
| Lost Productivity | 1,998.87 | 4,267.57 | 1,916.67 | 4,207.31 | 82.20 |  | -638.64 | | 803.05 | | 3,310.09 | 5,912.20 | 3,225.76 | 5,788.84 | 84.33 |  | -911.04 | 1,079.70 |
| Absenteeism | 1,751.79 | 4,077.25 | 1,688.22 | 4,045.98 | 63.57 |  | -627.30 | | 754.43 | | 2,221.10 | 5,045.67 | 2,038.51 | 4,910.03 | 182.59 |  | -664.41 | 1,029.59 |
| Presenteeism | 247.09 | 711.04 | 228.45 | 710.66 | 18.64 |  | -102.26 | | 139.54 | | 1,089.00 | 2,397.56 | 1,187.26 | 2,508.27 | -98.26 |  | -515.31 | 318.78 |
| **Perspective III: A+B+C+D+E+F  (SOCIETAL)** | **4,801.88** | **6,271.46** | **4,858.03** | **7,492.66** | **-56.15** |  | **-1,228.50** | | **1,116.21** | | **8,281.10** | **8,664.55** | **7,658.55** | **7,959.43** | **622.55** |  | **-793.98** | **2,039.07** |

Note: n = sample size. SD = standard deviation. PReDicT = PReDicT intervention group, TAU = Treatment-As-Usual group. LCL = lower confidence level. UCL = upper confidence level. Mean diff = mean(PReDiCT) – mean(TAU).
GBP prices converted to EUR using average monthly exchange rate GBP/EUR for 2018: 1.129474167, Retrieved from: Exchange rate (InforEuro), <https://ec.europa.eu/budget/graphs/inforeuro.html>.
^1^ Mean diff = mean(PReDiCT) – mean(TAU). Means costs by group and the adjusted difference were estimated using a generalized linear model (GLM) with family gamma and link log (cost as the dependent variable and treatment group as the independent variable), using the margins command, *** p<0.01, ** p<0.05, * p<0.1.

## Supplementary Table S18c: Mean cost differences per participant between the before- and during-trial periods - complete cases (€, year 2018, n=534)

|  | **BEFORE Trial vs. DURING Trial costs** | | | | | | | |
| --- | --- | --- | --- | --- | --- | --- | --- | --- |
|  | **PReDicT (n=274)** | | | | **TAU (n=260)** | | | |
|  | **Mean diff^1^** | **95% LCL** | **95% UCL** | **p-value** | **Mean diff^1^** | **95% LCL** | **95% UCL** | **p-value** |
| **A) Intervention (PReDicT test) costs** | **97.34** | **68.24** | **126.43** | **<0.001** | **0.000** | **0.000** | **0.000** | **.** |
| **B) Total medication costs** | **23.66** | **10.72** | **36.60** | **<0.001** | **31.778** | **17.172** | **46.383** | **<0.001** |
| Antidepressants (BNF chapter 04.03) | 17.06 | 12.42 | 21.69 | <0.001 | 23.782 | 10.502 | 37.062 | <0.001 |
| Other MH (BNF chapter 04.01/02/04) | 7.34 | -3.04 | 17.72 | 0.165 | 7.188 | 1.458 | 12.917 | 0.014 |
| Other Nervous System (BNF chapter 04.05-11) | -0.74 | -4.16 | 2.68 | 0.672 | 0.809 | -1.165 | 2.782 | 0.421 |
| **C) Mental Health Care** | **-270.12** | **-592.39** | **52.16** | **0.100** | **-416.706** | **-861.232** | **27.820** | **0.066** |
| MH Community Care | -448.70 | -609.62 | -287.78 | <0.001 | -477.824 | -641.489 | -314.159 | <0.001 |
| MH Outpatient Care | -31.87 | -104.40 | 40.65 | 0.388 | -84.856 | -198.285 | 28.573 | 0.142 |
| MH Inpatient Care | 210.46 | -34.85 | 455.77 | 0.092 | 145.974 | -220.847 | 512.796 | 0.434 |
| **A+B+C** | **-149.12** | **-473.14** | **174.91** | **0.366** | **-384.928** | **-829.609** | **59.753** | **0.090** |
| **D) Non-Mental Health Care** | **-800.52** | **-1,160.98** | **-440.07** | **<0.001** | **-344.699** | **-608.477** | **-80.921** | **0.011** |
| Primary Care | -148.64 | -171.95 | -125.33 | <0.001 | -167.763 | -195.287 | -140.240 | <0.001 |
| NMH Community Care | -168.67 | -256.01 | -81.32 | <0.001 | -56.690 | -99.922 | -13.457 | 0.010 |
| NMH Outpatient Care | -203.12 | -285.91 | -120.34 | <0.001 | -170.134 | -292.465 | -47.803 | 0.007 |
| NMH Inpatient Care | -280.09 | -582.94 | 22.75 | 0.070 | 49.888 | -189.629 | 289.405 | 0.682 |
| **Perspective I: A+B+C+D  (HEALTH CARE)** | **-949.64** | **-1,405.08** | **-494.20** | **<0.001** | **-729.627** | **-1321.248** | **-138.006** | **0.016** |
| **E) Social Care** | **-21.25** | **-53.44** | **10.94** | **0.195** | **-20.944** | **-94.700** | **52.813** | **0.577** |
| **Perspective II: A+B+C+D+E  (HEALTH AND SOCIAL CARE)** | **-970.89** | **-1,431.36** | **-510.42** | **<0.001** | **-750.571** | **-1348.556** | **-152.586** | **0.014** |
| **F) Indirect Costs** | **-2,508.34** | **-3,270.48** | **-1,746.19** | <0.001 | **-2,049.96** | **-2,824.72** | **-1,275.19** | **<0.001** |
| Informal Care | -228.55 | -518.97 | 61.87 | 0.123 | 20.252 | -268.823 | 309.328 | 0.890 |
| Transport Expenses | -12.04 | -19.38 | -4.70 | <0.001 | -11.709 | -17.530 | -5.887 | <0.001 |
| Lost Household Productivity | -956.53 | -1,155.73 | -757.32 | <0.001 | -749.405 | -948.059 | -550.752 | <0.001 |
| Lost Productivity | -1,311.22 | -1,906.02 | -716.42 | <0.001 | -1,309.10 | -1,927.47 | -690.72 | <0.001 |
| Absenteeism Cost | -469.31 | -1,014.22 | 75.60 | 0.09 | -350.29 | -897.19 | 196.62 | 0.21 |
| Presenteeism Cost | -841.91 | -1,125.99 | -557.83 | <0.001 | -958.81 | -1,242.09 | -675.53 | <0.001 |
| **Perspective III: A+B+C+D+E+F  (SOCIETAL)** | **-3,479.22** | **-4,423.72** | **2,534.72** | <0.001 | **-2,800.53** | **-3,815.15** | **-1,785.91** | **<0.001** |

Note: n = sample size. SD = standard deviation. PReDicT = PReDicT intervention group, TAU = Treatment-As-Usual group. LCL = lower confidence level. UCL = upper confidence level. Mean diff = mean(DURING trial costs) – mean(BEFORE Trial costs); ttest.

## Supplementary Table S19a: Health economic analysis sample characteristics at baseline - employed sample (n=638)

|  | **PReDicT (n=327)** | **TAU (n=311)** |
| --- | --- | --- |
| **Country, n (%)** |  |  |
| France | 25 (8%) | 28 (9%) |
| Germany | 38 (12%) | 40 (13%) |
| The Netherlands | 18 (6%) | 15 (5%) |
| Spain | 52 (16%) | 59 (19%) |
| United Kingdom | 194 (59%) | 169 (54%) |
| **Mean age at baseline (SD)** | 39.69 (12.54) | 39.37 (12.18) |
| **Female sex, n (%)** | 206 (63%) | 191 (61%) |
| **Ethnicity*, n (%)** |  |  |
| White | 273 (84%) | 256 (82%) |
| Racially minoritized groups* | 54 (16%) | 55 (18%) |
| **Mean length of education at baseline, years (SD)** | 14.36 (3.7) | 14.07 (3.59) |
| **Family history of depression, n (%)** |  |  |
| No | 157 (48%) | 139 (45%) |
| Yes | 170 (52%) | 172 (55%) |
| **Mean length of depression at baseline, years (SD)** | 4.13 (6.71) | 4.34 (6.70) |
| **Mean baseline QIDS-SR-16 (SD)** | 15.63 (4.56) | 15.92 (4.2) |
| **Mean baseline EQ-5D-5L index (SD) ^1^** | 0.723 (0.22) | 0.708 (0.19) |
| **Mean baseline EQ VAS (SD)** | 51.96 (20.40) | 50.14 (20.14) |
| **Mean baseline OxCAP-MH score (SD) ^2^** | 60.95 (12.99) | 58.4 (10.04) |

Note: n = sample size. SD = standard deviation. PReDicT = PReDicT intervention group, TAU = Treatment-As-Usual group. * A local ethical requirement prevented the collection of data on ethnicity from patients in France. *Includes: American Indian or Alaska Native, Asian, Black or African American, Native Hawaiian or Other Pacific Islander, Other.
QIDS-SR-16 = Quick Inventory of Depressive Symptoms, 16-item self-report version (score range 0–27). ^1^ Based on Ludwig K. et al. (2018) German value set for the EQ-5D-5L. Pharmacoeconomics. ^2^ Data were only collected in the UK and DE: PReDicT (n=170) and TAU (n=160).

## Supplementary Table S19b: Mean costs per participant - employed sample (€, year 2018, n=638)

|  | **DURING Trial (week 0 to week 24)** | | | | | | | | **BEFORE Trial vs. DURING Trial costs** | | | | | | | |
| --- | --- | --- | --- | --- | --- | --- | --- | --- | --- | --- | --- | --- | --- | --- | --- | --- |
|  | **PReDicT (n=327)** | | **TAU (n=311)** | | **Mean diff^2^** |  | **95% LCL** | **95% UCL** | **PReDicT (n=327)** | | | | **TAU (n=311)** | | | |
|  | **Mean** | **SD** | **Mean** | **SD** |  |  |  |  | **Mean diff^3^** | **p-value^3^** | **95% LCL** | **95% UCL** | **Mean diff^3^** | **p-value^3^** | **95% LCL** | **95% UCL** |
| **A) Intervention (PReDicT test) costs** | **82.88** | **220.84** | **0.00** | **0.00** | **82.88** |  | **58.85** | **106.91** | **82.88** | <0.001 | **58.85** | **106.91** | **.** | **.** | **.** | **.** |
| **B) Total medication costs** | **28.91** | **90.83** | **40.36** | **122.41** | **-11.45** |  | **-28.36** | **5.47** | **17.14** | <0.001 | **12.00** | **22.27** | **32.25** | <0.001 | **19.18** | **45.33** |
| Antidepressants (BNF chapter 04.03) | 14.95 | 34.20 | 22.32 | 95.01 | -7.37 |  | -17.43 | 2.68 | 14.86 | 0.000 | 11.14 | 18.57 | 21.53 | <0.001 | 10.95 | 32.11 |
| Other MH (BNF chapter 04.01/02/04) | 5.67 | 26.98 | 11.39 | 53.45 | -5.72 |  | -12.37 | 0.93 | 1.03 | 0.002 | 1.19 | 5.24 | 8.57 | 0.002 | 3.13 | 14.01 |
| Other Nervous System (BNF chapter 04.05-11) | 8.30 | 80.17 | 6.65 | 41.42 | 1.65 |  | -7.86 | 11.16 | -0.93 | 0.502 | -3.67 | 1.80 | 2.15 | 0.201 | -1.15 | 5.46 |
| **C) Mental Health Care** | **622.36** | **3,299.25** | **373.88** | **2,272.99** | **248.47** |  | **-202.67** | **699.62** | **-415.29** | **0.289** | **-1,185.21** | **354.63** | **-504.30** | **0.007** | **-867.27** | **-141.33** |
| **D) Non-Mental Health Care** | **174.08** | **609.64** | **243.57** | **1,120.55** | **-69.49** |  | **-203.96** | **64.98** | **-638.07** | <0.001 | **-940.05** | **-336.08** | **-402.95** | <0.001 | **-577.55** | **-228.36** |
| **Perspective I: A+B+C+D**  **(HEALTH CARE)** | **908.23** | **3,393.09** | **657.81** | **2,596.07** | **250.42** |  | **-220.44** | **721.28** | **-953.34** | **0.022** | **-1,767.98** | **-138.70** | **-875.00** | <0.001 | **-1,306.98** | **-443.02** |
| **Perspective II: A+B+C+D+E**  **(HEALTH AND SOCIAL CARE)** | **929.10** | **3,406.59** | **690.78** | **2,636.84** | **238.32** |  | **-235.37** | **712.01** | **-963.58** | **0.021** | **-1,779.47** | **-147.69** | **-896.79** | <0.001 | **-1,335.55** | **-458.03** |
| **F) Indirect Costs** | **6,019.45** | **7,448.19** | **6,364.21** | **7,571.69** | **-344.76** |  | **-1,513.85** | **824.33** | **-1,857.62** | **<0.001** | **-2,611.62** | **-1,103.63** | **-2,244.06** | **<0.001** | **-3,400.27** | **-1,087.84** |
| Informal Care | 327.40 | 1,436.51 | 473.61 | 2,256.35 | -146.21 |  | -436.65 | 144.22 | -222.97 | 0.050 | -445.80 | -0.14 | -492.08 | 0.255 | -1,341.10 | 356.95 |
| Transport Expenses | 4.77 | 42.47 | 1.22 | 4.75 | 3.55 | * | -0.16 | 7.25 | -10.21 | <0.001 | -16.43 | -4.00 | -10.98 | <0.001 | -15.84 | -6.13 |
| Lost Household Productivity | 2,030.82 | 1,795.48 | 2,003.35 | 1,784.10 | 27.47 |  | -250.33 | 305.26 | -535.22 | <0.001 | -701.11 | -369.32 | -422.62 | <0.001 | -596.32 | -248.92 |
| Lost Productivity | **3,656.46** | **6,287.59** | **3,886.03** | **5,983.67** | **-229.56** |  | **-1,187.65** | **728.52** | **-1,089.22** | **<0.001** | **-1,724.68** | **-453.75** | **-1,318.37** | **<0.001** | **-2,004.95** | **-631.80** |
| Absenteeism | 2,963.73 | 5,826.94 | 3,219.46 | 5,697.19 | -255.72 |  | -1,156.54 | 645.09 | -37.09 | 0.900 | -616.93 | 542.75 | 33.52 | 0.918 | -606.96 | 673.99 |
| Presenteeism | 692.74 | 1,870.66 | 666.58 | 1,751.35 | 26.16 |  | -254.96 | 307.27 | -1,052.12 | <0.001 | -1,340.85 | -763.38 | -1,351.88 | <0.001 | -1,655.68 | -1,048.08 |
| **Perspective III: A+B+C+D+E+F  (SOCIETAL)** | **6,948.54** | **9,098.95** | **7,054.98** | **8,469.25** | **-106.44** |  | **-1,474.19** | **1,261.31** | **-2,821.20** | **<0.001** | **-3,984.21** | **-1,658.18** | **-3,140.85** | **<0.001** | **-4,419.82** | **-1,861.88** |

Note: n = sample size. SD = standard deviation. PReDicT = PReDicT intervention group, TAU = Treatment-As-Usual group. .. = not available. LCL = lower confidence level. UCL = upper confidence level.
GBP prices converted to EUR using average monthly exchange rate GBP/EUR for 2018: 1.129474167, Retrieved from: Exchange rate (InforEuro), <https://ec.europa.eu/budget/graphs/inforeuro.html>. ^2^ Mean diff = mean(PReDiCT) – mean(TAU). Multiple imputation included treatment group, age, sex, country, and baseline costs as predictors. Means costs by group and the adjusted difference were estimated using a generalized linear model (GLM) with family gamma and link log (cost as the dependent variable and treatment group as the independent variable), using the margins command.^3^ Mean diff = mean(DURING trial costs) – mean(BEFORE Trial costs); ttest,

## Supplementary Table S20: Cost-effectiveness of PReDicT by analytical perspective - employed sample (€, year 2018, n=638)

| **Outcome** | **n** | **Cost** | **Outcome difference ^1^ (95% CI)** | **ICER** | **CE plane** | | | |
| --- | --- | --- | --- | --- | --- | --- | --- | --- |
|  |  | **difference ^1^** |  | **(95% CI)** |  |  |  |  |
|  |  | **(95% CI)** |  |  | **NE** | **SE** | **SW** | **NW** |
|  | **I: Health care perspective** | | | | | | | |
| HRQoL | 638 | **€ 250.42** | **0.0059** | **€42,652/QALY** | 75% | 13% | 2% | 10% |
| (EQ-5D-5L)^2^ |  | (-€220.44 to €721.28) | (-0.0041 to 0.0159) | (-€334,097/QALY to €416,451/QALY) |  |  |  |  |
| Capability | 441 | **€ 443.03** | **0.0046** | **€96,462/CWLY** | 71% | 3% | 1% | 25% |
| (OxCAP-MH)^3^ |  | (-€165.58 to €1,051.65) | (-0.0059 to 0.0151) | (-€2,160,652/CWLY to €1,015,824/CWLY) |  |  |  |  |
|  | **II: Health and social care perspective** | | | | | | | |
| HRQoL | 638 | **€ 238.32** | **0.0059** | **€40,591/QALY** | 70% | 18% | 2% | 10% |
| (EQ-5D-5L)^2^ |  | (-€235.37 to €712.01) | (-0.0041 to 0.0159) | (-€439,310/QALY to €390,223/QALY) |  |  |  |  |
| Capability | 441 | **€ 453.15** | **0.0046** | **€98,664/CWLY** | 72% | 2% | 1% | 25% |
| (OxCAP-MH)^3^ |  | (-€157.03 to €1,063.33) | (-0.0059 to 0.0151) | (-€845,340/CWLY to €1,309,749/CWLY) |  |  |  |  |
|  | **III: Societal perspective** | | | | | | | |
| HRQoL | 638 | **-€ 106.44** | **0.0059** | **-€18,129/QALY** | 32% | 57% | 3% | 8% |
| (EQ-5D-5L)^2^ |  | (-€1,474.19 to €1,261.31) | (-0.0041 to 0.0159) | (-€1,010,488/QALY to €632,486/QALY) |  |  |  |  |
| Capability | 441 | **€479.85** | **0.0046** | **€104,478/CWLY** | 47% | 25% | 6% | 22% |
| (OxCAP-MH)^3^ |  | (€-1,105.74 to €2,065.44) | (-0.0059 to 0.0151) | (-€1,783,140/CWLY to €2,040,863/CWLY) |  |  |  |  |

Note: n = sample size. QALYs = Quality-Adjusted Life Years. CWLY = Capability-Weighted Life Years. PReDicT = PReDicT intervention group. TAU = Treatment-As-Usual group. CI = confidence interval. ICER = incremental cost-effectiveness ratio.
^1^ difference = mean(PReDiCT) - mean(TAU); multiple imputation included treatment group, age, sex, baseline values (EQ-5D for HRQoL, OxCAP-MH index for capability, or baseline cost) as predictors; differences were estimated using regression models with treatment group as the explanatory variable. ^2^ Participants employed during the trial: PReDicT (n=327), TAU (n=311). ^3^ Data were only collected in the UK and DE; participants employed during the trial: PReDicT (n=232), TAU (n=209).

## Supplementary Table S21: Net monetary benefit of PReDicT in comparison to TAU - employed sample (€, year 2018, n=638)

| **Perspective** | **WTP threshold = €34,000/QALY** | | **WTP threshold = €50,000/QALY** | |
| --- | --- | --- | --- | --- |
|  | NMB | Probability PReDicT being cost-effective | NMB | Probability PReDicT being cost-effective |
|  | (95% CI) |  | (95% CI) |  |
| **I: Health care** | -€ 15 | 49% | € 85 | 59% |
|  | (-€635 to €1,029) |  | (-€656 to €787) |  |
| **II: Health and social care** | € 4 | 52% | € 102 | 63% |
|  | (-€633 to €1,035) |  | (-€645 to €779) |  |
| **III: Societal** | € 405 | 68% | € 503 | 71% |
|  | (-€1,212 to €1,947) |  | (-€1,184 to €2,130) |  |
| Note: WTP: Willingness-to-pay, NMB: Net monetary benefit, CI: Confidence interval. | | | |  |

## Supplementary Table S22a: Health economic analysis sample characteristics at baseline – OxCAP-MH sample (n=619)

|  | **PReDicT (n=311)** | **TAU (n=308)** |
| --- | --- | --- |
| **Country, n (%)** |  |  |
| Germany | 63 (20%) | 67 (22%) |
| United Kingdom | 248 (80) | 241 (78%) |
| **Mean age at baseline (SD)** | 37.43 (13.86) | 38.18 (14.42) |
| **Female sex, n (%)** | 182 (59%) | 181 (59%) |
| **Ethnicity*, n (%)** |  |  |
| White | 289 (93%) | 286 (93%) |
| Racially minoritized groups* | 22 (7%) | 22 (7%) |
| **(Self-)Employed during trial, n (%)** |  |  |
| **Yes** | 232 (75%) | 209 (68%) |
| **No** | 79 (25%) | 99 (32%) |
| Working age (18-65 years) | 73 (92%) | 92 (93%) |
| Past retirement age (>65 years) | 6 (8%) | 7 (7%) |
| **Mean length of education at baseline, years (SD)** | 13.92 (3.59) | 13.88 (3.3) |
| **Family history of depression at baseline, n (%)** |  |  |
| No | 146 (47%) | 147 (48%) |
| Yes | 165 (53%) | 161 (52%) |
| **Mean length of depression at baseline, years (SD)** | 3.39 (6.19) | 4.21 (7.50) |
| **Mean baseline QIDS-SR-16 (SD)** | 15.75 (4.39) | 15.80 (4.06) |
| **Mean baseline EQ-5D-5L index (SD) ^1^** | 0.709 (0.23) | 0.715 (0.19) |
| **Mean baseline EQ VAS (SD)** | 51.70 (21.24) | 50.98 (20.11) |
| **Mean baseline OxCAP-MH score (SD) ^2^** | 61.13 (14.60) | 58.6 (10.61) |

Note: n = sample size. SD = standard deviation. PReDicT = PReDicT intervention group, TAU = Treatment-As-Usual group. * A local ethical requirement prevented the collection of data on ethnicity from patients in France. *Includes: American Indian or Alaska Native, Asian, Black or African American, Native Hawaiian or Other Pacific Islander, Other.
QIDS-SR-16 = Quick Inventory of Depressive Symptoms, 16-item self-report version (score range 0–27). ^1^ Based on Ludwig K. et al. (2018) German value set for the EQ-5D-5L. Pharmacoeconomics. ^2^ Data were only collected in the UK and DE.

## Supplementary Table S22b: Mean costs per participant – OxCAP-MH sample (€, year 2018, n=619)

|  | **DURING Trial (week 0 to week 24)** | | | | | | | | **BEFORE Trial vs. DURING Trial costs** | | | | | | | |
| --- | --- | --- | --- | --- | --- | --- | --- | --- | --- | --- | --- | --- | --- | --- | --- | --- |
|  | **PReDicT (n=311)** | | **TAU (n=308)** | | **Mean diff^1^** |  | **95% LCL** | **95% UCL** | **PReDicT (n=311)** | | | | **TAU (n=308)** | | | |
|  | **Mean** | **SD** | **Mean** | **SD** |  |  |  |  | **Mean diff^2^** | **p-value^2^** | **95% LCL** | **95% UCL** | **Mean diff^2^** | **p-value^2^** | **95% LCL** | **95% UCL** |
| **A) Intervention (PReDicT test) costs** | **83.19** | **203.50** | **0.00** | **0.00** | **83.19** | ******* | **17.27** | **105.90** | **83.19** | **0.000** | **60.48** | **105.90** | **0.00** | **.** | **.** | **.** |
| **B) Total medication costs** | **31.24** | **135.81** | **35.24** | **138.87** | **-4.01** |  | **-25.80** | **17.79** | **25.95** | **0.001** | **10.69** | **41.21** | **29.46** | **0.000** | **14.76** | **44.15** |
| Antidepressants (BNF chapter 04.03) | 13.68 | 32.55 | 25.41 | 120.45 | -11.73 |  | -23.79 | 0.33 | 13.37 | 0.000 | 9.75 | 16.99 | 24.93 | 0.000 | 11.43 | 38.44 |
| Other MH (BNF chapter 04.01/02/04) | 13.43 | 115.49 | 3.79 | 24.16 | 9.64 |  | -2.12 | 21.40 | 13.17 | 0.045 | 0.29 | 26.05 | 3.75 | 0.007 | 1.04 | 6.45 |
| Other Nervous System (BNF chapter 04.05-11) | 4.13 | 24.06 | 6.04 | 50.33 | -1.92 |  | -7.78 | 3.95 | -0.59 | 0.706 | -3.65 | 2.47 | 0.78 | 0.358 | -0.88 | 2.43 |
| **C) Mental Health Care** | **659.42** | **3,582.90** | **315.12** | **2,179.99** | **344.30** |  | **-161.21** | **849.80** | **-134.71** | **0.604** | **-644.79** | **375.36** | **-594.37** | **0.001** | **-943.44** | **-245.31** |
| **D) Non-Mental Health Care** | **157.49** | **644.13** | **204.24** | **820.99** | **-46.75** |  | **-163.35** | **69.85** | **-490.78** | **0.002** | **-796.02** | **-185.54** | **-393.00** | **0.003** | **-651.22** | **-134.77** |
| **Perspective I: A+B+C+D**  **(HEALTH CARE)** | **931.34** | **3,711.29** | **554.60** | **2,376.15** | **376.73** |  | **-122.25** | **875.72** | **-516.36** | **0.084** | **-1,102.12** | **69.40** | **-957.91** | **0.000** | **-1,438.26** | **-477.57** |
| **Perspective II: A+B+C+D+E**  **(HEALTH AND SOCIAL CARE)** | **957.86** | **3,726.54** | **565.35** | **2,377.42** | **392.51** |  | **-108.79** | **893.81** | **-518.35** | **0.084** | **-1,106.59** | **69.89** | **-997.56** | **0.000** | **-1,479.95** | **-515.18** |
| **F) Indirect Costs** | **4,968.54** | **6,261.80** | **4,702.15** | **5,493.59** | **266.39** |  | **-660.15** | **1,192.93** | **-1,683.63** | **0.000** | **-2,350.29** | **-1,016.98** | **-2,074.75** | **0.000** | **-3,098.84** | **-1,050.67** |
| Informal Care | 333.86 | 1,494.82 | 250.44 | 683.82 | 83.42 |  | -89.08 | 255.92 | -395.39 | 0.006 | -674.39 | -116.39 | -651.38 | 0.120 | -1,474.00 | 171.23 |
| Transport Expenses | 1.06 | 4.21 | 0.94 | 3.39 | 0.12 |  | -0.48 | 0.72 | -9.71 | 0.000 | -12.34 | -7.09 | -10.54 | 0.000 | -13.07 | -8.01 |
| Lost Household Productivity | 2,103.34 | 1,882.80 | 2,178.71 | 1,876.57 | -75.37 |  | -371.79 | 221.04 | -561.96 | 0.000 | -734.13 | -389.79 | -347.20 | 0.000 | -530.25 | -164.16 |
| Lost Productivity | **2,530.27** | **5,150.43** | **2,272.05** | **4,444.00** | **258.22** |  | **-497.92** | **1,014.37** | **-716.57** | **0.006** | **-1,229.92** | **-203.22** | **-1,065.63** | **0.000** | **-1,563.77** | **-567.49** |
| Absenteeism | 2,005.20 | 4,601.62 | 1,830.02 | 4,111.60 | 175.18 |  | -511.55 | 861.90 | 97.46 | 0.659 | -337.13 | 532.05 | -75.73 | 0.740 | -523.92 | 372.45 |
| Presenteeism | 525.08 | 1,762.52 | 442.03 | 1,436.83 | 83.05 |  | -169.54 | 335.63 | -814.03 | 0.000 | -1,070.60 | -557.47 | -989.89 | 0.000 | -1,260.06 | -719.73 |
| **Perspective III: A+B+C+D+E+F  (SOCIETAL)** | **5,926.40** | **8,030.37** | **5,267.49** | **6,213.75** | **658.90** |  | **-463.85** | **1,781.65** | **-2,201.99** | **0.000** | **-3,134.51** | **-1,269.46** | **-3,072.32** | **0.000** | **-4,220.08** | **-1,924.56** |

Note: n = sample size. SD = standard deviation. PReDicT = PReDicT intervention group, TAU = Treatment-As-Usual group. LCL = lower confidence level. UCL = upper confidence level. Mean diff = mean(PReDiCT) – mean(TAU).
GBP prices converted to EUR using average monthly exchange rate GBP/EUR for 2018: 1.129474167, Retrieved from: Exchange rate (InforEuro), <https://ec.europa.eu/budget/graphs/inforeuro.html>.
^1^ Mean diff = mean(PReDiCT) – mean(TAU). Multiple imputation included treatment group, age, sex, country, and baseline costs as predictors. Means costs by group and the adjusted difference were estimated using a generalized linear model (GLM) with family gamma and link log (cost as the dependent variable and treatment group as the independent variable), using the margins command, *** p<0.01, ** p<0.05, * p<0.1. ^2^ Mean diff = mean(DURING trial costs) – mean(BEFORE Trial costs); ttest.

## Supplementary Table S23: Outlier-adjusted mean costs per participant (€, year 2018, n=913)

|  | **DURING Trial (week 0 to week 24)** | | | | | | | | **BEFORE Trial vs. DURING Trial costs** | | | |
| --- | --- | --- | --- | --- | --- | --- | --- | --- | --- | --- | --- | --- |
|  | **PReDicT (n=460)** | | **TAU (n=453)** | | **Mean diff^1^** |  | **95% LCL** | **95% UCL** | **PReDicT (n=460)** | | **TAU (n=453)** | |
|  | **Mean** | **SD** | **Mean** | **SD** |  |  |  |  | **Mean diff^2^** | **p-value^2^** | **Mean diff^2^** | **p-value^2^** |
| **A) Intervention (PReDicT test) costs** | **93.18** | **244.54** | **0.00** | **0.00** | **93.18** |  | **-115.73** | **-70.63** | **93.18** | <0.001 | 0.00 | . |
| **B) Total medication costs** | **24.61** | **49.57** | **31.07** | **74.05** | **-6.46** |  | **-14.49** | **1.56** | **17.89** | **<0.001** | **23.81** | **<0.001** |
| Antidepressants (BNF chapter 04.03) | 15.36 | 33.52 | 16.03 | 42.52 | -0.67 |  | -5.62 | 4.27 | 15.04 | <0.001 | 15.39 | <0.001 |
| Other MH (BNF chapter 04.01/02/04) | 5.34 | 23.86 | 8.31 | 44.67 | -2.98 |  | -7.46 | 1.51 | 3.23 | <0.001 | 6.34 | <0.001 |
| Other Nervous System (BNF chapter 04.05-11) | 3.92 | 26.98 | 6.73 | 40.57 | -2.82 |  | -7.45 | 1.82 | -0.37 | 0.723 | 2.08 | 0.079 |
| **C) Mental Health Care** | **495.30** | **2,043.81** | **336.24** | **1,798.59** | **159.06** |  | **-102.68** | **420.81** | **-257.37** | **<0.001** | **-463.89** | **<0.001** |
| MH Community Care | 122.72 | 427.41 | 87.83 | 323.56 | 34.89 |  | -14.67 | 84.45 | -407.11 | <0.001 | -474.52 | <0.001 |
| MH Outpatient Care | 58.97 | 415.63 | 78.41 | 485.60 | -19.45 |  | -79.25 | 40.36 | -41.50 | 0.097 | -48.95 | 0.095 |
| MH Inpatient Care | 313.61 | 1,829.20 | 169.99 | 1,332.58 | 143.62 |  | -81.76 | 369.00 | 191.24 | 0.025 | 59.58 | 0.414 |
| **A+B+C** | **613.09** | **2,083.60** | **367.31** | **1,812.46** | **245.78** | ***** | **-31.11** | **522.67** | **-146.30** | **0.162** | **-440.07** | **<0.001** |
| **D) Non-Mental Health Care** | **180.60** | **617.47** | **189.28** | **563.07** | **-8.68** |  | **-85.65** | **68.29** | **-445.47** | **<0.001** | **-396.52** | **<0.001** |
| Primary Care | 16.10 | 55.86 | 13.55 | 39.57 | 2.55 |  | -3.64 | 8.74 | -139.74 | <0.001 | -152.38 | <0.001 |
| NMH Community Care | 17.04 | 98.66 | 22.04 | 97.61 | -5.01 |  | -18.21 | 8.20 | -94.68 | <0.001 | -59.71 | <0.001 |
| NMH Outpatient Care | 49.26 | 256.96 | 70.70 | 312.01 | -21.44 |  | -59.71 | 16.83 | -169.35 | <0.001 | -137.88 | <0.001 |
| NMH Inpatient Care | 98.20 | 488.27 | 82.99 | 357.79 | 15.22 |  | -39.67 | 70.10 | -41.69 | 0.367 | -46.54 | 0.286 |
| **Perspective I: A+B+C+D**  **(HEALTH CARE)** | **793.69** | **2,193.93** | **556.59** | **1,936.78** | **237.10** | ***** | **-41.74** | **515.94** | **-591.76** | **<0.001** | **-836.59** | **<0.001** |
| **E) Social Care** | 20.00 | 93.02 | 17.05 | 80.15 | 2.95 |  | -8.32 | 14.22 | -16.43 | 0.214 | -28.93 | 0.144 |
| **Perspective II: A+B+C+D+E**  **(HEALTH AND SOCIAL CARE)** | **813.69** | **2,206.02** | **573.64** | **1,943.23** | **240.05** | ***** | **-39.56** | **519.66** | **-608.19** | **<0.001** | **-865.52** | **<0.001** |
| **F) Indirect Costs** | **5,050.40** | **6,550.45** | **5,112.78** | **6,316.72** | **-62.39** |  | **-898.01** | **773.24** | **-1,545.75** | **<0.001** | **-1,499.02** | **<0.001** |
| Informal Care | 319.16 | 983.34 | 330.03 | 863.06 | -10.86 |  | -131.31 | 109.58 | -247.61 | <0.001 | -179.84 | 0.040 |
| Transport Expenses | 1.54 | 5.24 | 1.53 | 6.31 | 0.01 |  | -0.74 | 0.76 | -10.46 | <0.001 | -9.66 | <0.001 |
| Lost Household Productivity | 2,130.42 | 1,839.73 | 2,113.34 | 1,807.30 | 17.09 |  | -219.49 | 253.66 | -456.72 | <0.001 | -294.14 | <0.001 |
| Lost Productivity | 2,599.27 | 5,552.69 | 2,667.89 | 5,273.81 | -68.62 |  | -772.13 | 634.89 | -830.95 | <0.001 | -1,015.38 | <0.001 |
| Absenteeism | 2,106.83 | 5,091.58 | 2,210.27 | 4,949.39 | -103.44 |  | -756.34 | 549.46 | -66.54 | 0.754 | -68.15 | 0.767 |
| Presenteeism | 492.45 | 1,607.56 | 457.63 | 1,483.06 | 34.81 |  | -165.74 | 235.37 | -764.41 | <0.001 | -947.22 | 0.000 |
| **Perspective III: A+B+C+D+E+F  (SOCIETAL)** | **5,864.09** | **7,190.78** | **5,686.43** | **6,743.22** | **177.66** |  | **-726.48** | **1,081.81** | **-2,153.94** | **<0.001** | **-2,364.53** | **<0.001** |

Note: n = sample size. SD = standard deviation. PReDicT = PReDicT intervention group, TAU = Treatment-As-Usual group. .. = not available. LCL = lower confidence level. UCL = upper confidence level.
GBP prices converted to EUR using average monthly exchange rate GBP/EUR for 2018: 1.129474167, Retrieved from: Exchange rate (InforEuro), <https://ec.europa.eu/budget/graphs/inforeuro.html>. ^1^ Mean diff = mean(PReDiCT) – mean(TAU). Multiple imputation included treatment group, age, sex, country, and baseline costs as predictors. Means costs by group and the adjusted difference were estimated using a generalized linear model (GLM) with family gamma and link log (cost as the dependent variable and treatment group as the independent variable), using the margins command, *** p<0.01, ** p<0.05, * p<0.1. ^2^ Mean diff = mean(DURING trial costs) – mean(BEFORE Trial costs); ttest,

## Supplementary Table S24: Cost-effectiveness of PReDicT from different analytical perspectives (costs inflated to 2024 Euros)

| **Outcome** | **n** | **Cost  difference ^1^  (95% CI)** | **Outcome difference ^1^ (95% CI)** | **ICER (95% CI)** | **CE plane** | | | |
| --- | --- | --- | --- | --- | --- | --- | --- | --- |
|  |  |  |  |  | **NE** | **SE** | **SW** | **NW** |
|  | **I: Health care perspective** | | | | | | | |
| HRQoL  (EQ-5D-5L) | 913 | **€257**  (-€273 to €788) | **0.0039**  (-0.0051 to 0.0128) | **€66,722/QALY**  (-€872,580/QALY to €862,654/QALY) | 45% | 23% | 10% | 22% |
| Capability  (OxCAP-MH) | 619 | **€460**  (-€149 to €1,070) | **0.0056**  (-0.0034 to 0.0146) | **€82,156/CWLY**  (-€805,211/CWLY to €549,008/CWLY) | 83% | 6% | 1% | 10% |
|  | **II: Health and social care perspective** | | | | | | | |
| HRQoL  (EQ-5D-5L) | 913 | **€256**  (-€278 to €790) | **0.0039**  (-0.0051 to 0.0128) | **€66,334/QALY**  (-€671,072/QALY to €729,708/QALY) | 45% | 22% | 11% | 22% |
| Capability  (OxCAP-MH) | 619 | **€479**  (-€133 to €1,092) | **0.0056**  (-0.0034 to 0.0146) | **€85,597/CWLY**  (-€807,913/CWLY to €636,976/CWLY) | 84% | 6% | 0% | 10% |
|  | **III: Societal perspective** | | | | | | | |
| HRQoL  (EQ-5D-5L) | 913 | **€121**  (-€1,074 to €1,316) | **0.0039**  (-0.0051 to 0.0128) | **€31,390/QALY**  (-€1,319,281/QALY to €1,458,948/QALY) | 32% | 36% | 10% | 22% |
| Capability  (OxCAP-MH) | 619 | **€805**  (-€567 to €2,176) | **0.0056**  (-0.0034 to 0.0146) | **€143,690/CWLY**  (-€1,662,219/CWLY to €1,977,302/CWLY) | 76% | 14% | 1% | 10% |

Note: n = sample size. QALYs = Quality-Adjusted Life Years. CWLY = Capability-Weighted Life Years. PReDicT = PReDicT intervention group. TAU = Treatment-As-Usual group. CI = confidence interval. ICER = incremental cost-effectiveness ratio.
^1^ difference = mean(PReDiCT) - mean(TAU); multiple imputation included treatment group, age, sex, baseline values (EQ-5D for HRQoL, OxCAP-MH index for capability, or baseline cost) as predictors; differences were estimated using regression models with treatment group as the explanatory variable. Total costs were inflated from 2018 to 2024 using the NHS Cost Inflation Index (pay and prices) reported in Jones et al., Unit Costs of Health and Social Care 2024 (60).

# Figures

## Supplementary Figure S1


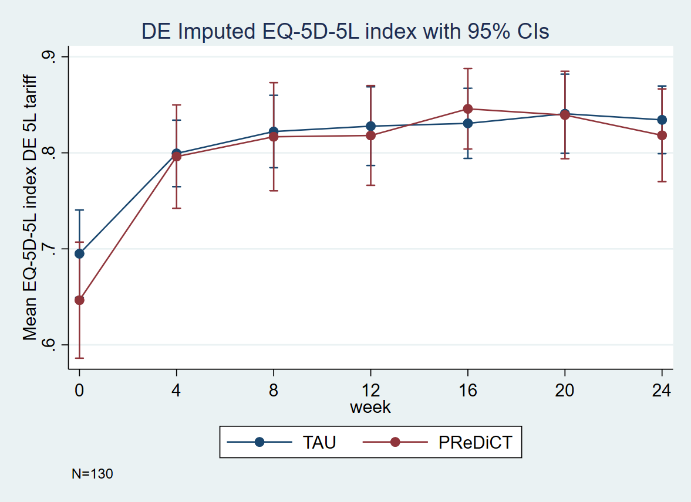

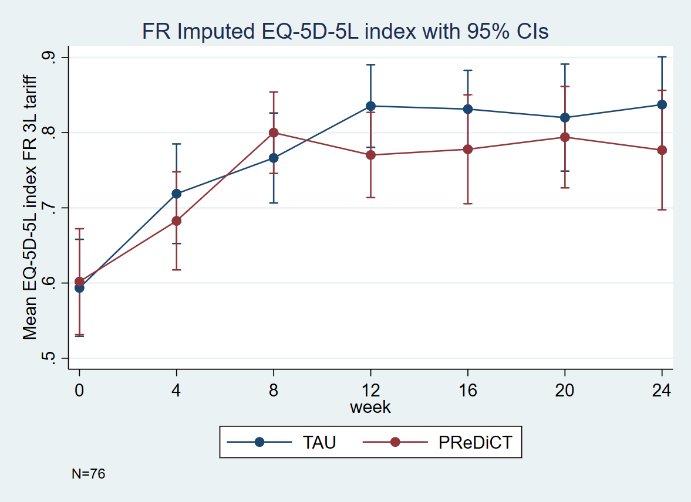

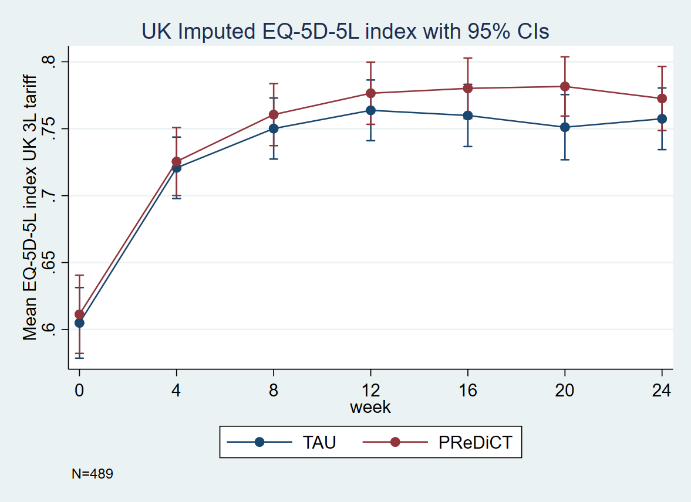

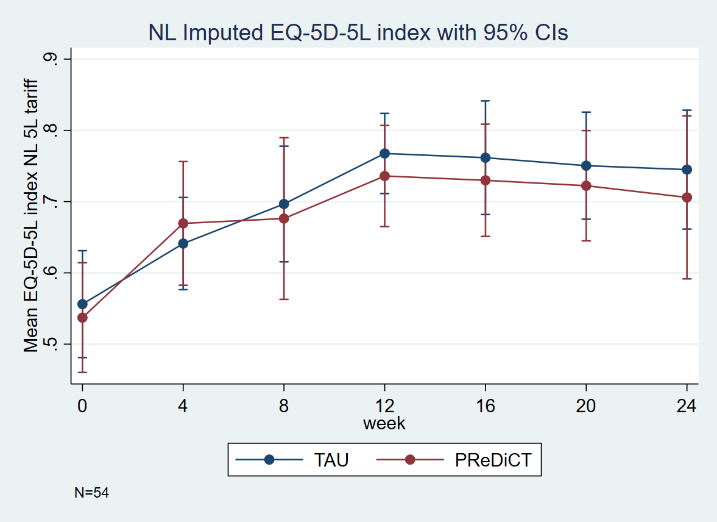


**Supplementary Figure S1: EQ-5D-5L utility index by country (n=913)**

Note: n = sample size. PReDicT = PReDicT intervention group. TAU = Treatment-As-Usual group.
CI = confidence interval. FR = France, DE = Germany, NL = The Netherlands, ES = Spain, UK = United Kingdom.

## Supplementary Figure S2

(a)


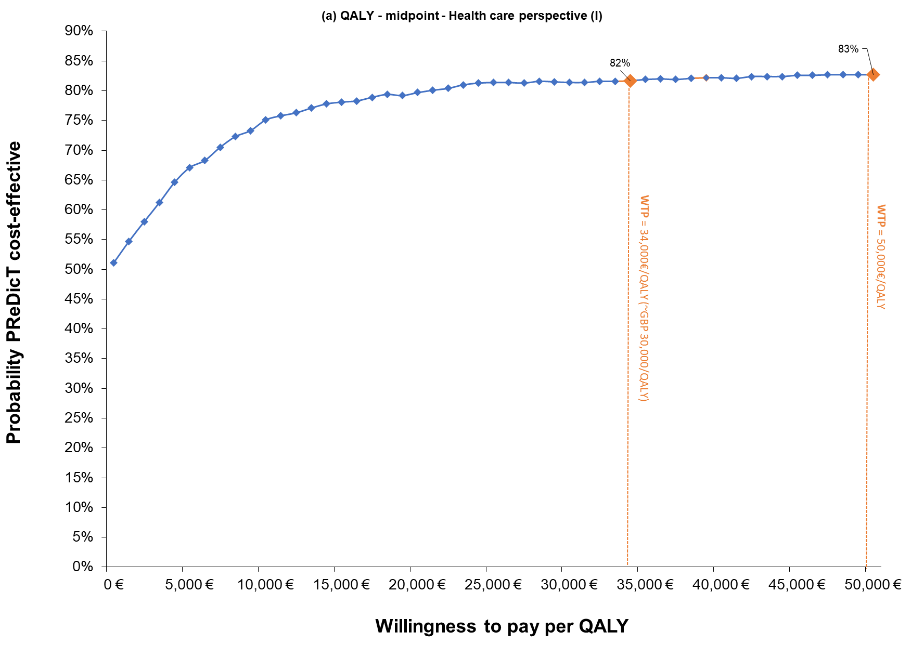


(b)


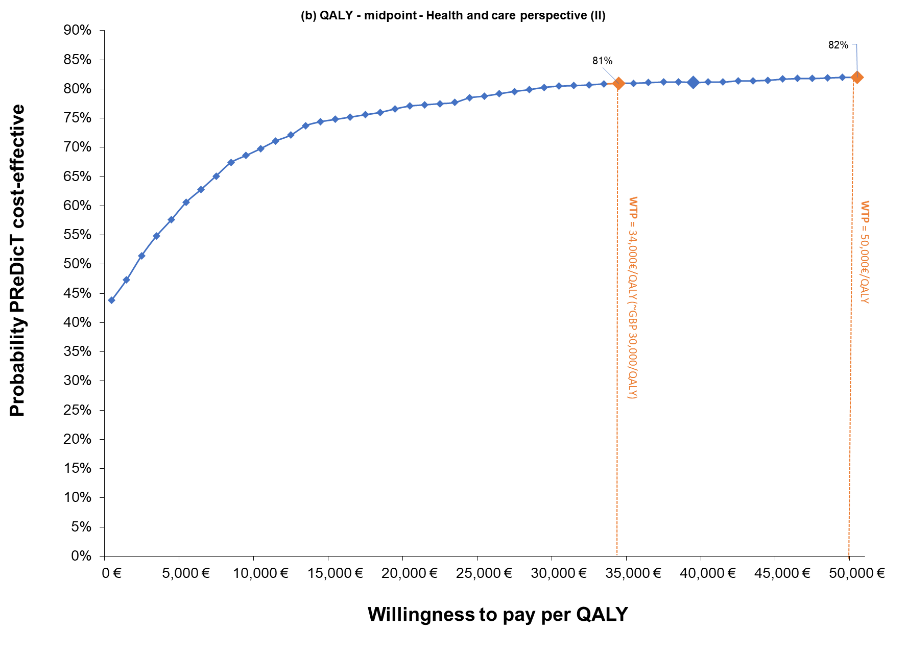


(c)


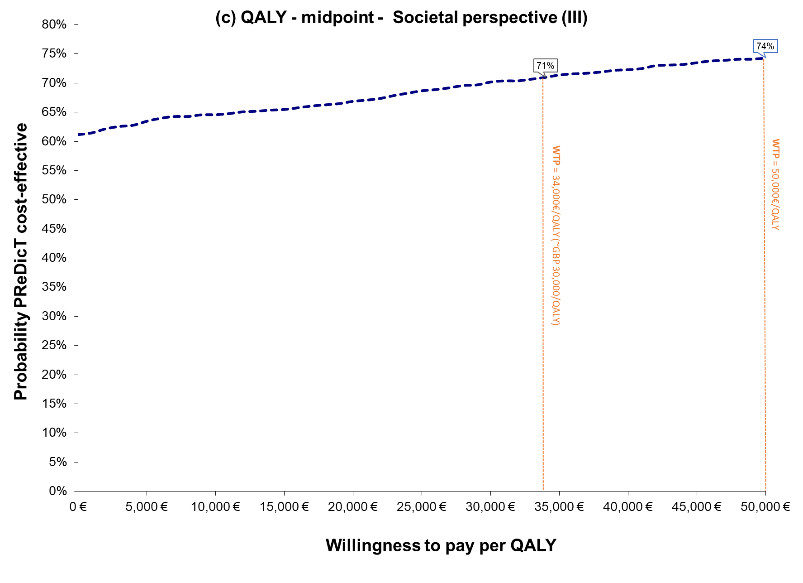


**Supplementary Figure S2: Cost-effectiveness acceptability curves (CEACs) by analytical perspective - United Kingdom (n=489)**

Note: (a) health care perspective (I), (b) health and social care perspective (II), (c) societal perspective (III)

# References

1. Vidal France. Médicaments 2019 [Available from: <https://www.vidal.fr/>.

2. Rote Liste. Arzneimittelverzeichnis für Deutschland (einschließlich EU-Zulassungen und bestimmter Medizinprodukte). Rote Liste Service GmbH, Frankfurt/Main. 2018.

3. NICE - National Institute for Health and Care Excellence. BNF 76: September 2018 - March 2019 2018 [updated 03 May 2018. Available from: <https://bnf.nice.org.uk/>.

4. Zorginstituut Nederland. Farmacotherpaeutisch Kompas 2019 [Available from: <https://www.farmacotherapeutischkompas.nl>.

5. Vademecum. Gui farmacologica - Medicamentos 2019 [Available from: <https://www.vademecum.es/>.

6. Rapp T, Andrieu S, Chartier F, Deberdt W, Reed C, Belger M, et al. Resource Use and Cost of Alzheimer’s Disease in France: 18-Month Results from the GERAS Observational Study. Value in Health. 2018;21(3):295–303.

7. Bock JO, Brettschneider C, Seidl H, Bowles D, Holle R, Greiner W, et al. Ermittlung standardisierter Bewertungssätze aus gesellschaftlicher Perspektive für die gesundheitsökonomische Evaluation- [Calculation of standardised unit costs from a societal perspective for health economic evaluation]. Gesundheitswesen. 2015;77(1):53–61.

8. Curtis L, Burns A. Unit Costs of Health and Social Care 2018: PSSRU; 2018.

9. Goorden M, van der Feltz-Cornelis C, van Steenbergen-Weijenburg K, Horn E, Beekman A, van Hakkaart-van Roijen L. Cost-utility of collaborative care for the treatment of comorbid major depressive disorder in outpatients with chronic physical conditions. Neuropsychiatric Disease and Treatment. 2017;2017:13:1881–93.

10. Departament de salut. ORDRE SLT/212/2018, de 13 de diciembre. Diari Oficial de la Generalitat de Catalunya, número 7770, 18 de diciembre de 2018 2018 [Available from: [https://dogc.gencat.cat/ca/pdogc_canals_interns/pdogc_sumari_del_dogc/?anexos=1&selectedYear=2018&selectedMonth=11&numDOGC=7770&language=ca_ES_0#](https://dogc.gencat.cat/ca/pdogc_canals_interns/pdogc_sumari_del_dogc/?anexos=1&selectedYear=2018&selectedMonth=11&numDOGC=7770&language=ca_ES_0).

11. Grupp H, König H-H, Konnopka A. Kostensätze zur monetären Bewertung von Versorgungsleistungen bei psychischen Erkrankungen. Gesundheitswesen. 2017;79(01):48–57.

12. Hakkaart-van Roijen L, Van der Linden N, Bouwmans C, Kanters T, Tan S. Methodology of costing research and reference prices for economic evaluations in healthcare [in Dutch: Kostenhandleiding: Methodologie van kostenonderzoek en referentieprijzen voor economische evaluaties in de gezondheidszorg]: Zorginstituut Nederland - National Health Care Institute; 2016 [updated February 2016. Available from: <https://www.zorginstituutnederland.nl/over-ons/publicaties/publicatie/2016/02/29/richtlijn-voor-het-uitvoeren-van-economische-evaluaties-in-de-gezondheidszorg>

<https://www.imta.nl/costingtool/>.

13. Obiluke Consulting. eSalud Platform 2018 [Available from: <http://www.oblikue.com/en/esalud.html>.

14. Fernández A, Mendive JM, Conejo-Cerón S, Moreno-Peral P, King M, Nazareth I, et al. A personalized intervention to prevent depression in primary care: cost-effectiveness study nested into a clustered randomized trial. BMC Medicine. 2018;16(1):28.

15. L’Etablissement Public de Santé Maison Blanche. TARIFS DES PRESTATIONS / FRAIS D’HOSPITALISATION 2019 [Available from: <http://www.ch-maison-blanche.fr/Usagers/Tarifs-des-prestations-Frais-d-hospitalisation>.

16. Mavranezouli I, Megnin-Viggars O, Cheema N, Howlin P, Baron-Cohen S, Pilling S. The cost-effectiveness of supported employment for adults with autism in the United Kingdom. Autism. 2014;18(8):975–84.

17. Curtis L. Unit Costs of Health and Social Care 2014. Canterbury: Personal Social Services Research Unit, University of Kent; 2014.

18. NHS - National Health Service. NHS reference costs 2017 to 2018 2018 [Available from: <https://improvement.nhs.uk/resources/national-cost-collection/>.

19. L'Assurance Maladie-AMELI. Consultations en métropole : vos remboursements 2019 [updated 11 Mar 2019. Available from: <https://www.ameli.fr/assure/remboursements/rembourse/consultations/metropole>.

20. Kanters TA, Bouwmans-Frijters C, van der Linden N, Tan SS, van Hakkaart-van Roijen L. Update of the Dutch manual for costing studies in health care. PloS one. 2017;12.

21. Salvador-Carulla L, Bendeck M, Fernandez A, Alberti C, Sabes-Figuera R, Molina C, et al. Costs of depression in Catalonia (Spain). Journal of affective disorders. 2011;132(1-2):130–8.

22. Un instant pour moi. LES MASSAGES/TARIFS 2019 [Available from: <http://uninstantpourmoi.e-monsite.com/pages/descriptions-des-massages.html>

<https://massage.ooreka.fr/comprendre/prix-massage>

<http://www.zeninstitut.com/tarifs.html>.

23. MBSR-Würzburg. Achtsamkeit als Weg 2019 [Available from: <https://mbsr-wuerzburg.jimdo.com/>, <https://bewegungs-raum.de/mbsr-stressbewaeltigung-durch-achtsamkeit/>, <https://www.mbsr-nidda.de/kurse-termine-im-%C3%BCberblick/>; 4. source: <https://www.mbsrinfrankfurt.de/mbsr-gruppe.html>.

24. Curtis L, Burns A. Unit Costs of Health and Social Care 2017: PSSRU; 2017.

25. Consejería de Sanidad. Orden SAN/35/2017 de 15 de diciembre de 2017. Boletín Oficial de Cantabria, núm. 248, 29 de diciembre de 2017. 2017.

26. Mi Masaje. Masajes Antiestrés 2019 [Available from: <https://www.mimasaje.es/masaje-antiestres/>, <https://www.milanuncios.com/anuncios/masajes-relajantes-barcelona.htm>.

27. Reiki Barcelona. Reiki Barcelona 2019 [Available from: <https://www.reiki-barcelona.cat/precio-sesion-individual-reiki/>, <https://www.cronoshare.com/servicios/sesiones-de-reiki/barcelona/barcelona>, <https://terapiareiki.es/precios/>, <https://camidesalut.com/tratamientos-naturales/reiki-barcelona/>.

28. Grupp H, König H-H, Riedel-Heller S, Konnopka A. FIMPsy – Fragebogen zur Inanspruchnahme medizinischer und nicht medizinischer Versorgungsleistungen bei psychischen Erkrankungen: Entwicklung und Verwendung. Psychiat Prax. 2017(EFirst).

29. Hospital Sainte-Anne. TARIF DES PRESTATIONS 2018 [Available from: <http://www.ch-sainte-anne.fr/content/download/27174/375891/file/Tarifs%20prix%20de%20journe%CC%81e%20CHSA%201er%20avril%202018.pdf>

30. Sebastiennormier-sophrologue. Récapitulatif des tarifs des prestations du cabinet de sophrologie 2019 [Available from: <https://sebastiennormier-sophrologue.fr/tarif-des-seances-de-sophrologie/>, <https://www.sophrologue-hypnose.com/services-tarifs>; <https://www.sophro-zoe.fr/index.php?IdPage=1539783952>.

31. Scansante. L’Etude nationale des coûts (ENC) d'une activité de médecine, chirurgie, obstétrique et odontologie (MCO) 2019 [Available from: <https://www.scansante.fr/applications/enc-mco/submit?snatnav=&annee=2017&secteur=dgf&type_activite=ghs&cmd=01&souscmd=01M&racine=01M21&ghm=01M21T&mbout=dummy&num_selection=01M21T&type_selection=ghm&zip=non> &

32. Curtis L. Unit Costs of Health and Social Care 2012. Canterbury: Personal Social Services Research Unit, University of Kent; 2012.

33. Curtis L. Unit Costs of Health and Social Care 2010. Canterbury: Personal Social Services Research Unit, University of Kent; 2010.

34. NHS National Services Scotland. Scottish National Tariff - File Listings 2017/18 2018 [updated 20 Nov 2018. Available from: <https://www.isdscotland.org/Health-Topics/Finance/Costs/File-Listings-2018.asp>.

35. Departament de salut. ORDRE SLT/30/2013, de 20 de febrer, Català de la Salut. Diari Oicial de la Generalitat de Catalunya Núm. 6323 – 26.2.2013. 2013.

36. Curtis L, Burns A. Unit Costs of Health and Social Care 2015: PSSRU; 2015.

37. L'Assurance Maladie-AMELI. Ambulances : les tarifs conventionnels 2019 [updated 11 Mar 2019. Available from: <https://www.ameli.fr/transporteur-sanitaire/exercice-professionnel/facturation/tarifs/ambulances-tarifs-conventionnels>.

38. Feuerwehr Frankfurt. Benutzungsentgelte im Rettungsdienstbereich Frankfurt am Main 2019 [Available from: <http://www.feuerwehr-frankfurt.de/index.php/mediathek/rettungsdiensttraeger?download=161:benutzungsentgelte-rettungsdienst> , <http://www.boerdekreis.de/bi/vo020.asp?VOLFDNR=1078>

39. Mapa-assurance. Tarif accupuncture 2019 [Available from: <https://www.mapa-assurances.fr/Actualites/remboursement-frais-sante/acupuncture-remboursement-secu-mutuelle>. 2. source: <https://acupuncture.ooreka.fr/comprendre/prix-acupuncture>. 3. source: <https://www.mutuelle-miltis.fr/guides/lacupuncture/>

40. Kostenblick.de. Was kostet eine Akupunktur? 2018 [updated 01 Aug 2018. Available from: <https://kostenblick.de/was-kostet-eine-akupunktur/>.

41. DÄGfA. Was kostet Akupunktur? 2019 [Available from: <https://www.daegfa.de/PatientenPortal/Akupunktur.Was_kostet_Akupunktur.aspx>

42. acupunctuurmaassluis. Prijzen 2019 [Available from: <https://www.acupunctuurmaassluis.nl/prijzen/>.

43. Clinicpoint. Sesion de acupunctura 2019 [Available from: 1. source: <https://www.clinicpoint.com/barcelona/medicina-tradicional/acupuntura/sesion-de-acupuntura>; 2. source: ; 3. source: <https://www.smartsalus.com/barcelona/consulta-de-osteopatia-en-barcelona-4051>.

44. Gozlan G, Lecardeur L, Monfort AS, Doz M, Ortiz I, Larroumets P, et al. Analyse coût-efficacité de l’aripiprazole injectable à libération prolongée (AILP) comparé au palmitate de palipéridone (PP) dans le traitement de la schizophrénie en France. L'Encéphale. 2018;44(6):496–503.

45. Bendeck M, Serrano-Blanco A, García-Alonso C, Bonet P, Jordà E, Sabes-Figuera R, et al. An integrative cross-design synthesis approach to estimate the cost of illness: An applied case to the cost of depression in Catalonia. Journal of Mental Health. 2013;22(2):135–54.

46. Krauth C, Hessel F, Hansmeier T, Wasem J, Seitz R, Schweikert B. Empirische Bewertungssätze in der gesundheitsökonomischen Evaluation-ein Vorschlag der AG Methoden der gesundheitsökonomischen Evaluation (AG MEG) - [Empirical standard costs for health economic evaluation in Germany -- a proposal by the working group methods in health economic evaluation]. Gesundheitswesen. 2005;67(10):736–46.

47. Office for National Statistics. Earnings and hours worked, care workers: ASHE Table 26. Annual Survey of Hours and Earnings time series of selected estimates2018.

48. Statistisches Bundesamt (Destatis). Verdienste und Arbeitskosten - Arbeitnehmerverdienste 2017 2018 [Available from: <https://www.destatis.de/DE/Publikationen/Thematisch/VerdiensteArbeitskosten/Arbeitnehmerverdienste/ArbeitnehmerverdiensteJ.html>.

49. INSEE-Institut national de la statistique et des études économiques. Les salaires dans le secteur privé et les entreprises publiques en 2015 - Déclaration annuelle de données sociales (DADS) - Insee Résultats 2018 [updated 08 Aug 2018. Available from: <https://www.insee.fr/fr/statistiques/3577665?sommaire=3578367#consulter-sommaire>.

50. INE- Instituto Nacional de Estadistica. Salario anual medio, mediano y modal. Salario por hora. Brecha salarial de género (no ajustada) en salarios por hora 2019 [Available from: <https://www.ine.es/dynt3/inebase/index.htm?padre=2129&capsel=2129>.

51. INSEE-Institut national de la statistique et des études économiques. Salaire minimum interprofessionnel de croissance (Smic) 2019 [Available from: <https://www.insee.fr/fr/statistiques/1375188>.

52. BOE - - Boletin Oficial del Estado. Boletin Oficial del Estado, núm. 317, de 30 de diciembre de 2017, Real Decreto 1077/2017, de 29 de diciembre, por el que se fija el salario mínimo interprofesional para 2018, páginas 130800 a 130803 2017 [Available from: <https://www.boe.es/eli/es/rd/2017/12/29/1077>

53. Office for National Statistics. Earnings and hours worked, all employees: ASHE Table 1. Annual Survey of Hours and Earnings time series of selected estimates2018.

54. CBS. Employment; jobs, wages, working hours; key figures 2019 [Available from: <https://opendata.cbs.nl/statline/#/CBS/en/dataset/81431ENG/table?ts=1552045002245>.

55. Eurostat. HICP (2015 = 100) - annual data (average index and rate of change) [prc_hicp_aind] 2019 [Available from: <https://appsso.eurostat.ec.europa.eu/nui/submitViewTableAction.do>.

56. EuroQoL. EQ-5D-5L Crosswalk Index Value Calculator 2019 [Available from: <https://euroqol.org/eq-5d-instruments/eq-5d-5l-about/valuation-standard-value-sets/crosswalk-index-value-calculator/>.

57. Van Hout B, Janssen M, Feng Y-S, Kohlmann T, Busschbach J, Golicki D, et al. Interim scoring for the EQ-5D-5L: mapping the EQ-5D-5L to EQ-5D-3L value sets. Value in health. 2012;15(5):708–15.

58. Chevalier J, de Pouvourville G. Valuing EQ-5D using time trade-off in France. The European Journal of Health Economics. 2013;14:57–66.

59. NICE - National Institute for Health Care Excellence. Position statement on use of the EQ-5D-5L valuation set for England (updated November 2018). 2019. London: National Institute for Health and Care Excellence (NICE); 2018.

60. Jones, Karen C. and Weatherly, Helen and Birch, Sarah and Castelli, Adriana and Chalkley, Martin and Dargan, Alan and Findlay, Douglas and Gao, Minyue and Hinde, Seb and Markham, Sarah and Smith, Deb and Teo, Hansel (2025) Unit Costs of Health and Social Care 2024 Manual. Technical report. Personal Social Services Research Unit (University of Kent) & Centre for Health Economics (University of York), Kent, UK 10.22024/UniKent/01.02.109563. (doi:10.22024/UniKent/01.02.109563)
